# Regional genetic diversity for NNV grouper viruses across the Indo-Asian region – implications for selecting virus resistance in farmed groupers

**DOI:** 10.1038/s41598-017-11263-4

**Published:** 2017-09-06

**Authors:** Wayne Knibb, Giang Luu, H. K. A. Premachandra, Ming-Wei Lu, Nguyen Hong Nguyen

**Affiliations:** 1 GeneCology Research Centre, Faculty of Science, Health, Education and Engineering, University of the Sunshine Coast, Maroochydore, QLD 4558 Australia; 2 Research Institute for Aquaculture No. 1, Dinh Bang, Tu Son, Bac Ninh Vietnam; 3 Department of Aquaculture, National Taiwan Ocean University, Keelung, Taiwan

**Keywords:** Evolution, Genetics

## Abstract

Grouper aquaculture around Asia is impacted by the nervous necrosis virus (NNV) and, in response, host resistance to this infection is being considered as a trait for selection. However efficient selection may be confounded if there are different genetic strains of NNV within and between regions and over years. This study uses statistical approaches and assessment of “characteristic attributes” (i.e. nucleotide positions that discriminate among strains) to assess whether published and new NNV RNA2 cds sequences show genetic differentiation over geography, host species and years. Rather clear evidence was found for regional strains of NNV. Interestingly, most of the geographic defining “characteristic attributes” were in codon position three, and not translated into differences for the protein capsid (i.e. they were synonymous variations), suggesting that while NNV strains were geographically isolated and had diverged in different regions for RNA sequences, selection had largely conserved the protein sequences among regions. The apparent selection constraint on the capsid protein may mitigate the risk that despite geographic subdivision, NNV strain variability will confound genetic selection for host resistance. The existence of regional Asian NNV strains may suggest that hatcheries are at risk from NNV not only from imported material but also from endemic reservoirs.

## Introduction

Recently new genetic selection and domestication programs have commenced for large marine fish^1^. Various groupers (subfamily *Epinephelinae* of the family *Serranidae*) including giant groupers (*Epinephelus lanceolatus*), tiger groupers (*E. fuscoguttatus*) and humpback groupers (*Cromileptes altivelis*), compose one important new group of large marine species for domestication and selection. However, a viral disease, “viral nervous necrosis (VNN)” has been reported across grouper production centers in Taiwan, Malaysia, Indonesia and elsewhere around Asia^2, 3^. The causative agent of VNN in groupers and other marine fish is the nervous necrosis virus (NNV) of the genus Betanodavirus, within the family Nodaviridae (International Committee on Taxonomy of Viruses) (Harikrishnan *et al*.^4^ for review).

Many authors believe NNV is a serious threat to future marine fish aquaculture (including groupers). Pakingking *et al*.^5^ concluded “Catastrophic mortalities inflicted by piscine betanodaviruses remain as a major deterrent in the sustainable aquaculture of several species of groupers reared in ponds and floating net-cages in the open sea”. In a similar vein, Harikrishnan *et al*.^4^ concluded “viral nervous necrosis (VNN) caused by nervous necrosis virus (NNV) is one of the most important viral diseases that cause mass mortality in more than 39 marine fish species in 10 families”. Likewise Munday *et al*.^6^ concluded “In the last decade betanodavirus infections have emerged as major constraints on the culture of marine fish in all parts of the world”. Several recent reviews recognize betanodavirus as a significant problem for marine fish farming almost world wide^7–9^.

Fish host NNV resistance could be a key trait for future selection and the estimated heritability for NNV resistance in Atlantic cod appears to be high^10–12^. Moreover QTLs for resistance to NNV in Asian Sea Bass were reported^13^. However, notwithstanding such promising genetic selection opportunities, there are substantial knowledge gaps about the genetic diversity of NNV among Asian grouper hosts: are there many strains within and between regions, perhaps with different virulence so that each region or NNV strain may then require different selection programs, or not? Moreover, is there evidence that NNV strains vary over time? Genetic programs are long term and expensive undertakings so selecting for resistance to the appropriate strain/s is an important consideration, but one with a relative absence of knowledge for Asian grouper NNV. Without some clarity, we may select for inappropriate NNV strains, or see new NNVs transported from a different region for which the grouper have no resistance.

On a global scale, and considering many fish host species, previous phylogenetic analyses have resolved certain geographic and other patterns for NNV. For example there appears to be clustering of VNN genotypes into four main groups that include the so called barfin flounder nervous necrosis virus (BFNNV), the tiger puffer nervous necrosis virus (TPNNV), the striped jack nervous necrosis virus (SJNNV) and the red spotted grouper nervous necrosis virus (RGNNV) based on molecular phylogenetic analysis and percent nucleotide identity^14^. These four clusters are somewhat associated with water temperatures (and also hosts), for example there is a tendency for BFNNV and TPNNV genotypes to occur in temperatures up to 20 °C, for SJNNV to occur from 20 to 25 °C and for RGNNV for occur from 25 up to 30 °C^15^.

Within the red spotted grouper nervous necrosis virus (RGNNV) strain, to which all Asian grouper NNV belong, however, no one so far has reported evidence of genetic subgrouping by region, species or year in a formal statistical manner, especially when we restrict hosts just to Asian grouper. Available Genbank data show all Asian grouper NNV RNA2 RNA sequences, from Japan to Indonesia, are very closely related, varying by just one or two percent; this closeness means there are challenges to determine phylogenetic relationships especially when using traditional DNA or RNA distance based methods. However, Lowenstein *et al*.^16^ have found for tuna that particular polymorphic nucleotide positions (characteristic attributes) may categorically discriminate groups/ species previous not previous distinguished using traditional analyses based on percent RNA or DNA similarity. Ruan *et al*.^17^ reported certain polymorphic nucleotide positions could discriminate among strains of the SARS virus while Zou *et al*.^18^ reported that “character based” bar coding methods outperformed other approaches in discriminating closely related sea snails. Within aquaculture, these approaches of identifying nucleotides that are present in one group but not others have yet to be considered to a large degree, although our group have applied these ideas of characteristic attributes to recently resolve a very long standing taxonomic issue in oysters, whether the very closely related *Crassostrea gigas* and *C. angulata* are one or two species, as a prelude to conducting selection on the now identified *C. angulata* in Vietnam^19^.

The goal of this report was to collate the most comprehensive data set to date on NNV RNA2 sequences for warm water Asian marine finfish, whether published and/or lodged in Genbank over the last 20 years, including some sequence data produced by our group for Vietnamese and Taiwanese grouper, to statistically test the data for evidence of NNV strain variation that associates with geography, host species and year and also to determine whether there are “characteristic attributes” that indicate regional (or host, year) specific differences among the strains. This knowledge will help guide future selection criteria/NNV strains to consider in future genetic selection programs.

## Materials and Methods

### New samples from Vietnam and Taiwan

As a prelude to sequencing, a large number of grouper samples (nearly 200) were collected as part of routine Veterinary surveillance, from different species and hybrids from the National Broodstock Centre for Mariculture Species, NBCFMS in Cat Ba, Haiphong, Northern Vietnam in September 2015 and from Qieding, Kaohsiung, Taiwan in July 2015 (Supplementary Fig. S1) and tested for the presence of NNV using primers from Nishizawa *et al*.^20^. Samples were taken, typically, at a time there were general mortalities of larvae, but other than being alive or dead, no other distinctive behaviours were noted; i.e. larval fish in this study looked normal. Note: the lack of symptoms for larval fish infected with NNV was also reported^21^.

### RNA extraction and sequencing

Total RNA was isolated from the tissue samples using Trisure reagent according to the manufacturer’s instructions (Bioline, AUS). cDNA synthesis was conducted from the total genomic RNA using the QuantiTect Reverse Transcription kit (Qiagen) following manufacturer’s instructions. Briefly, a genomic DNA elimination reaction was performed by mixing a solution of 2 µL of dissolved template RNA (500 ng/µl) with 2 µL g DNA Wipeout Buffer 7x and 10 µL RNase-free Water with an incubation at 42 °C for 2 minutes, then placed immediately on ice. The reverse transcription reaction was carried out for 30 minutes at 42 °C and then incubated at 95 °C for 3 minutes with the reaction mixture with final volume of 20 µL: 4 µL Quantiscript RT Buffer, 1 µL Quantiscript Reverse Transcriptase, 1 µL RT Primer Mix and 14 µL genomic DNA elimination.

The RNA2 segment of nervous necrosis virus was amplified using the set of primers described in Ransangan and Manin^3^. The forward and reverse PCR reactions were conducted separately in a 50 μL total volume consisting of 25 μL MyTaq™ Red Mix (Bioline, AUS), 19 μL Rnase-free water, 2 μL each primer (10 µM) (forward and reverse) and 2 μL cDNA. The amplifications were performed using an Eppendorf Mastercycler Pro Thermal Cycler with an initial denaturation at 94 °C for 3 minutes, followed by 30 cycles of denaturation at 94 °C for 30 seconds, annealing at 58 °C for 30 seconds and extension at 72 °C for 30 seconds and final extension of 72 °C for 5 minutes. PCR products from these amplifications were subsequently sent to Macrogen Inc. (Seoul, Korea) for sequencing. Sequencing reactions were conducted using the ABI PRISM Big Dye TM Terminator Cycle Sequencing Ready Reaction Kit (PE Biosystems, Foster City, CA, USA) and electrophoresis by the ABI PRISM 3730xl DNA Sequencer. Nucleotide sequences were analysed and edited using BioEdit v 7.0.5.

### Collation of published sequences

Genbank (the National Centre for Biotechnology Information (NCBI) (http://www.ncbi.nlm.nih.gov/)) was searched in various ways for NNV sequences. First, the complete coding RNA sequences for the NNV RNA2 gene from a giant grouper host (GB accession number AY721615) was blasted against all sequences in Genbank, and over 500 sequences recovered with at least 25% similarity. All complete coding RNA sequences from all groupers in the Indo-Asian region were retained along with those from any other species that had similarity (to AY721615) at least equal to the most distant NNV from a grouper host, these non grouper species included mostly barramundi (*Lates calcarifer*). Second, Genbank was searched for “Grouper” and “NNV” and “RNA2”, and all NNV complete coding RNA sequences from the Indo-Asian region collated. A total of 75 sequences were collated. To this we added (details following) a further four unpublished sequences reported for the first time here from Vietnamese humpback grouper (*C. altivelis*) and three from Taiwanese Giant x Tiger hybrids (*E. lanceolatus* ♂ x *E. fuscoguttatus* ♀) (Supplementary Table S2). Only the protein encoding region of the sequences was used for analyses. Full protein capsid encoding sequences of all 82 sequences, published and new, is given in Supplementary Table S2.

### Analyses of sequences

**Figure 1:**
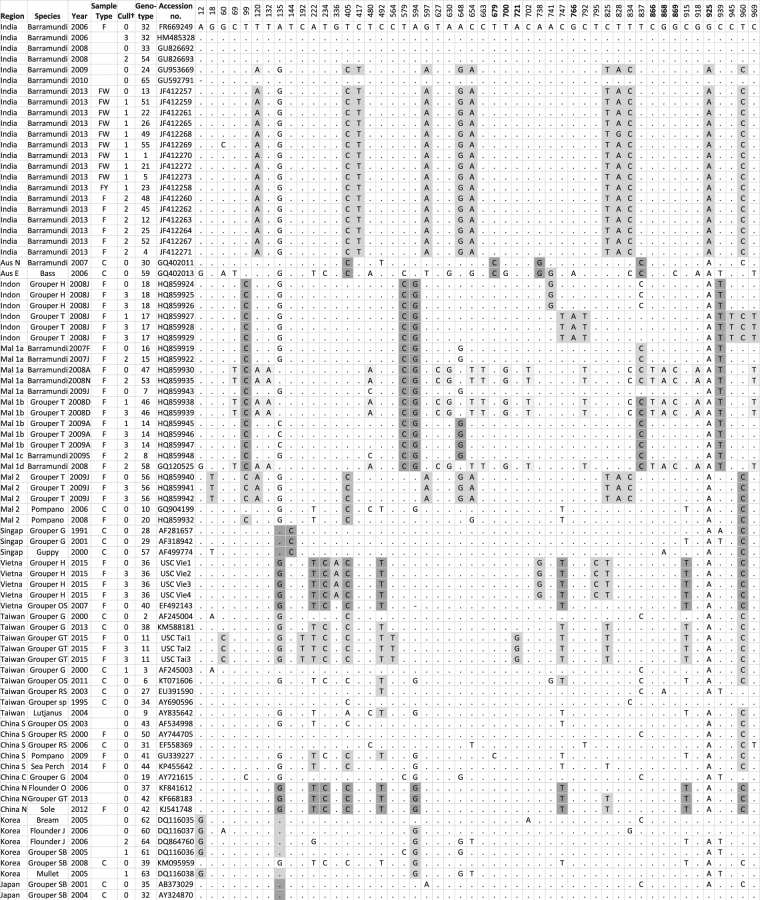
“Characteristic attributes” for RNA2 NNV gene. Abbreviations for column 1: Aus N = North Australia, Aus E = East Australia, Indon = Indonesia, Mal 1a = Kota Kinabalu, Sabah, Malaysia, Mal 1b = Tuaran, Sabah; Malaysia, Mal 1c = Sandakan, Sabah, Malaysia, Mal 1d = UMS hatchery, Sabah, Malaysia, Langkawi; Mal 2 = West Malaysia, Singap = Singapore, Vietna = Vietnam, Taiwan = Taiwan, China S = South China, China C = Central China, China N = North China, Korea = Korea, Japan = Japan.† Different groups that were culled to reduce impact of pseudo-replication, see details in following text. Abbreviations for column 4: F = fish, FW = fish wild, FY = fish wild yearling, C = cell culture. Additional tests for regional differences were conducted using Bayesian Tip-association Significance (BaTS) tests; the association index and the parsimony score statistic assessing regional differences among all samples were both statistically significant (P < 0.05) as were tests of monophyletic clade (MC) sizes for each region, except for Japan and South China.

Alignment of nucleotide sequences was performed using CLUSTAL W^22^. In the 1017 bp ORF encoding the coat protein there were 230 variable nucleotide sites which were considered, and referred to, as “loci” for the following tests. Analysis of molecular variance (GenAlEx^23^) assessed the probabilities for differences among regions over all loci, and for each locus separately. Each region group needed at least two cases for statistical testing, so for example, the testing for region differences would have had 13 regions considering the 82 samples (Fig. 1). A region typically corresponds to a country, such as Japan or Taiwan, but for large countries where sites were separated by significant geography, such as North vs South China and East (Borneo) vs West (Peninsular) Malaysia, regions within countries were considered.

To address the possibility of lack of independence of some samples arising from a short term spread and propagation of a new infection of one viral genotype in a given hatchery, the data were also analysed at each of various culling steps that attempt to remove potentially non independent samples within hatcheries. First, 15 samples that had the exact same sequence (genotype) as another case from the same location, same year and same species were removed; 67 samples were available after this culling. The second culling included given samples that came from the same location, year and host species as another sample (leaving only one sample per species in a given place and time); 55 samples were available after this round of culling. The intention was to remove cases that may represent propagation in a hatchery, so an exception to the rule is that wild fish samples even if from the same species and location were not culled at this stage. Last, the third culling was for samples that came from the same site and same year as other samples; 40 samples remained after this round of culling. At this stage, even different wild fish were culled if they came from the same site at the same year. Similar culling procedures applied when testing for “species” grouping, and for “year” grouping. When a group contained only one case after culling, the single case was pooled with a neighbouring group (for region and year) or included in a group termed “other” for the species analyses.

Construction of phylogenetic trees based on RNA2 nucleotide and amino acid sequences were conducted by MEGA 7^24^ using the maximum likelihood method.

### Statistical ANOVA model

The analysis of variance (ANOVA) model assessing jointly effects of location, species and year on “percent RNA sequence relatedness” to a consensus sequence take the following form:

*y*_*ijkl*_ = *µ* + *R*_*i*_ + *S*_*j*_ + *Y*_*k*_ + *e*_*ijkl*_

Where

y_*ijklmn*_ = observations, i.e. “percent RNA sequence relatedness” to a consensus sequence

*µ* = mean

*R*_*i*_ = region type

*S*_*j*_ = species

*Y*_*k*_ = year

*e*_*ijkl*_ = error/residual

Analyses were conducted using SPSS software.

### Relative synonymous codon usage analysis

Relative synonymous codon counts were calculated for each of the main host fish species groups, namely, grouper and barramundi, using MEGA 7^24^ and counts were compared for differences between groups using a Chi Square test.

### Bayesian Tip-association Significance testing (BaTS)

Phylogeny-trait (geographic location) associations were tested including 80 sequences (2 Australian samples were removed since they were appeared to be outliers) using Bayesian Tip-association Significance testing (BaTS) software^25^. Posterior sample of trees (PST) were obtained through Bayesian Markov-Chain Monte Carlo (MCMC) analysis using BEAST software^26^. BEAST was run for 10 million MCMC chain lengths (sampled every 1000 generations; burn-in 10%) with the Hasegawa-Kishino-Yano + Gamma (HKY + G) model, assuming coalescent constant population priors with relaxed lognormal clock^27^. The convergence for all parameters (effective sample size >200) was assessed using TRACER v1.6 (http://beast.bio.ed.ac.uk/Tracer). The resulting trees were used to test whether each region was a monophyletic clade (i.e. assessed for phylogeny-trait statistics considering 10 geographic regions - India, Malaysia 1, Malaysia 2, Singapore, Vietnam, Taiwan, China South, China North, Korea and Japan) using BaTS software^25^.

### Data availability

All data used in this report are available in the Supplementary Figures and Tables.

## Results

### New RNA2 sequenced from Vietnam and Taiwan in this study

Four *C. altivelis* Vietnamese larvae and three *E. lanceolatus* ♂ × *E. fuscoguttatus* ♀ larvae from Taiwan were sequenced for the RNA2 gene.

The 1338 bp RNA2 sequence from all four Vietnamese and three Taiwanese samples had a single 1017 bp ORF encoding the coat protein. The coding region of the coat protein is predicted to include 338 amino acids (protein molecular mass of 37.07 kilodaltons), starting at ATG codon (nucleotide 4) and terminating with stop codon TAA (nucleotide 1017). The NNV sequences obtained from Vietnamese Humpback grouper (*C. altivelis*) in this study are submitted to GenBank with the accession number KU705815.

### VNN sequences from Asian grouper used in the statistical analyses

The protein capsid encoding RNA sequences from the 82 collated NNV RNA2 sequences had high similarity considering the 1017 bp protein encoding regions (all sequences in Supplementary Table S2, similarity matrix in Supplementary Fig. S3), with an average similarity between pairs of 98.1%, and the most distant pair had 91.5% identity. This maximum distance involved Australia bass which has a rather atypical sequence from all other sequences. Removal of this one outlier showed the average similarity between pairs was 98.3%, and the most distant pair had 96.0% identity; comparable similarity percentages restricting the data set just to groupers (n = 39) were 98.5% and 96.5% respectively. There were 230 nucleotide positions of the 1017 that varied over the cds, and most analyses focused on these variable nucleotide positions (Supplementary Table S4).

### Characteristic attributes

#### Regional grouping

Regional differences were evident for a large number of nucleotides positions. Analysis of molecular variance tests (AMOVA^23^) recorded, out of 230 polymorphic sites, 38 nucleotide positions statistically significantly from zero considering region at P ≤ 0.05; an additional 36 at P ≤ 0.01; and the probability when testing all loci together was ≤0.01 (data showing significance levels for each nucleotide position of Fig. 1 are in Supplementary Fig. S5). While there were 1017 nucleotides in the complete coding sequences in each of the 82 samples considered, only 230 contained two or more nucleotide variants at a given nucleotide position and would qualify as candidates for statistical testing. However, of the 230, just, 102 contained three or more different alleles; every case of P ≤ 0.01 was restricted to nucleotide positions where there were three or more different bases at a given nucleotide position; this probably reflected the power of the statistical test and relationship with level of polymorphism. Thus we can say that of 102 moderately polymorphic nucleotide sites (with at least three variable nucleotides), more than half recorded statistically significant differences among regions.

Every Indonesian and East Malaysian (Borneo) sample invariably had the same (less common) nucleotides at positions 100, 581, 596 and 942 (although a smattering of samples from other regions also had the same nucleotides as some of these positions). These characteristic attributes, hereafter referred to as “CATS”, were present in two different grouper species as well as barramundi, were present in multiple sites within Borneo and evident in all (and different) years and collection times (2007 to 2009). Considered separately, each of the “CATS” in highly statistically significant (Fig. 1 and Supplementary Fig. S5), and *together*, with a single exception (AY721615), they comprise a diagnostic genotype unique for Borneo and Indonesia irrespective of host fish species.

“CATS” were also evident for Vietnam, and all samples share the same nucleotides at positions 136, 223, 235, 407, 494, 749, 917, 963. *Together* they comprise a diagnostic genotype almost unique to Vietnam but interestingly all samples from Norther China share, with one exception (596), the same genotype for these “CATS”, and the Chinese samples come from three different species (flounder, sole, grouper) and from three different years (2006, 2012, 2013). While four of the five Vietnamese samples came from the same species, same location and same year (Humpback grouper, North Vietnam, 2015), the fifth, with the same “CAT” genotype, came from a different species and different year (Orange Spotted Grouper, 2007). Together with the data from Northern China, this described genotype would indicate these samples are not randomly drawn from a general population, nor are they solely outcomes of propagation in a given hatchery (although the four Humpback Grouper samples may be).

Other “CATS”, where only one nucleotide is found in all samples in a given region, are highlighted in darker grey in Fig. 1; “CATS” where a given country mostly has one nucleotide at a given position is highlighted in medium grey, and signature genotypes, that characterise a region, composed of a number of “CATS” are given in light grey (see accession numbers HQ859930, HQ859935, HQ859938, HQ859939).

#### Culling to reduce impact of “pseudo-replication”

Removing all 15 samples that had identical sequences to other same species samples from the same site and year (cases designated by a “3”, column 5, Fig. 1) left 67 remaining sequences for analysis (cases designated by a “0”, “1” or “2”, column 5, Fig. 1); this made little change to the probabilities for regional differences recorded using all 82 samples (Supplementary Fig. S5).

Likewise, removing a further 12 hatchery samples which came from the same location, year and host species as other samples (cases designated by a “2”, column 5, Fig. 1), left 55 samples for analysis (cases designated by a “0” or “1”, column 5, Fig. 1), but did not substantially alter the pattern of significant cases among regions (Supplementary Fig. S5). Wild fish were except from culling at this step as the intention was to remove cases of pseudo-replication in the hatcheries.

The last culling was for a further 15 samples that came from the same site and same year as other samples (cases designated by a “1”, column 5, Fig. 1), leaving 40 samples for analysis (cases designated by a “0”, Fig. 1). Even wild fish meeting these criteria were culled at this point. With these 40 samples, the incidence of significant cases was reduced somewhat, but even in this most extreme vetting, AMOVA analyses showed there were still a total of eight cases (nucleotide positions) with statistically significant differences among regions at P ≤ 0.05, a total of eight cases (nucleotide positions) with statistically significant differences among regions at P ≤ 0.01 and the test over all loci (all nucleotide positions) was significant (P ≤ 0.01) (Supplementary Fig. S5). After culling, there were only 65 nucleotide (allele) positions with three or more different nucleotides (the rest of the 230 were now monomorphic or had one to two nucleotide differences among the 40 samples). So we can say that of 65 moderately polymorphic nucleotide sites (with at least three variable nucleotides of the 40 NNV samples), more than 10% (i.e. eight) recorded highly statistically significant nucleotide differences among regions even under extreme culling (which is about a ten fold higher rate of highly significant cases than that expected by chance alone).

#### Species grouping

Species differences at given nucleotide positions were also detected considering all samples, albeit at about half the incidence as that detected above for regional differences (AMOVA recorded 16 nucleotide positions statistically significantly from zero, P ≤ 0.05; 21 with P ≤ 0.01 and the overall probability when testing all loci together was P ≤ 0.01 (Supplementary Fig. S5). There appeared to be a high degree of coincidence in the probability levels for nucleotides considering regions and considering species (r = 0.637, P < 0.001, considering all 230 nucleotide positions). “CATS” that were predominately restricted for one fish host species were not typical, with some rare exceptions (e.g. nucleotide 193); within countries, “CATS” are shared across species, e.g. barramundi and grouper share common “CATS” in East Malaysia and Indonesia (Fig. 1). On the other hand, with one exception (Column 6, Fig. 1), different species did not have completely identical RNA2 sequences.

The various culling steps to reduce “pseudo-replication” culminated in the most extreme culling with 40 samples remaining (cases designated by a “0”, Fig. 1) and relative few cases of statistically significant differences among species, namely five cases of statistically significant nucleotide differences among species, only one at P < 0.01 from among 65 moderately polymorphic nucleotide sites (i.e. those with at least three variable nucleotides). Such an incidence of statistically significant results could be accounted for by chance or Type I error. The test over all loci was not statistically significant (P > 0.05).

#### Year grouping

Yearly differences were also detected considering all samples, again at about half the incidence as that detected above for regional differences (AMOVA recorded 14 statistically significantly from zero, P ≤ 0.05; 26 with P ≤ 0.01 and the overall probability when testing all loci together was P ≤ 0.01; Supplementary Fig. S5). Again, there appeared to be a high degree of coincidence in the probability levels for nucleotides considering regions and considering years (r = 0.642, P < 0.001, considering all 230 nucleotide positions).

The various culling steps to reduce “pseudo-replication” culminated in the most extreme culling with 40 samples remaining (cases designated by a “0”, Fig. 1) and then there was only one case of a statistically significant difference among species, namely one at P < 0.01 from among 65 moderately polymorphic nucleotide sites (i.e. those with at least three variable nucleotides). The test over all loci was not statistically significant (P > 0.05).

For some regions, the same “CATS” were recorded over years, for example, the “CATS” at nucleotides position 144 for Singapore were evident from 1991 through to 2000, and several “CATS” for North China (nucleotide positions 135, 222, 234, 405, etc.) were evident from 2006 to 2012. Analogous results were evident for Vietnam and Western Malaysia (Borneo) but not for India. On the other hand, with one exception (Column 6, Fig. 1), different years did not have completely identical RNA2 sequences.

### Analysis of variance using RNA percent similarity as the dependent variate

A consensus sequence for all the polymorphic nucleotide sequences was generated (Supplementary Table S4), then “percent RNA similarity” to the consensus was calculated for each sample (Supplementary Fig. S6); this variate ranged from 67.3% identity (for Australian bass) to 98.6% for Orange Spot grouper. The bass seemed to be an outlier as the next most divergent sample was Malaysian barramundi at 87.3% similarity, noting all these percentages refer to just the polymorphic nucleotide data, and not the whole coding region sequences. On one hand, the metric of “percent RNA similarity” contains less information than the preceding data on variable nucleotide positions, but on the other hand, “percent RNA similarity”, as a continuous variate, permits *simultaneous* partitioning of variance among the predictor variates (location, species, and year).

An ANOVA (Section 6.3.1 Materials and Methods), using all the samples and the three predictor variates (region, species, year), showed that all predictor factors accounted for a significant part of the variation in “percent RNA similarity” (for region, ANOVA F_11,47_ = 12.135, P < 0.001; for species, ANOVA F_11,47_ = 2.840, P < 0.01; for year, ANOVA F_12,47_ = 4.056, P < 0.001). Repeating these analyses, but after the most extreme culling (removing all samples that come from the same year and site as another) still indicated highly statistically significant differences only for region (for region, ANOVA F_10,6_ = 14.288, P < 0.01; for species, ANOVA F_10,6_ = 4.589, P < 0.05; for year, ANOVA F_11,6_ = 0.794, P > 0.05).

### Maximum Likelihood tree

**Figure 2:**
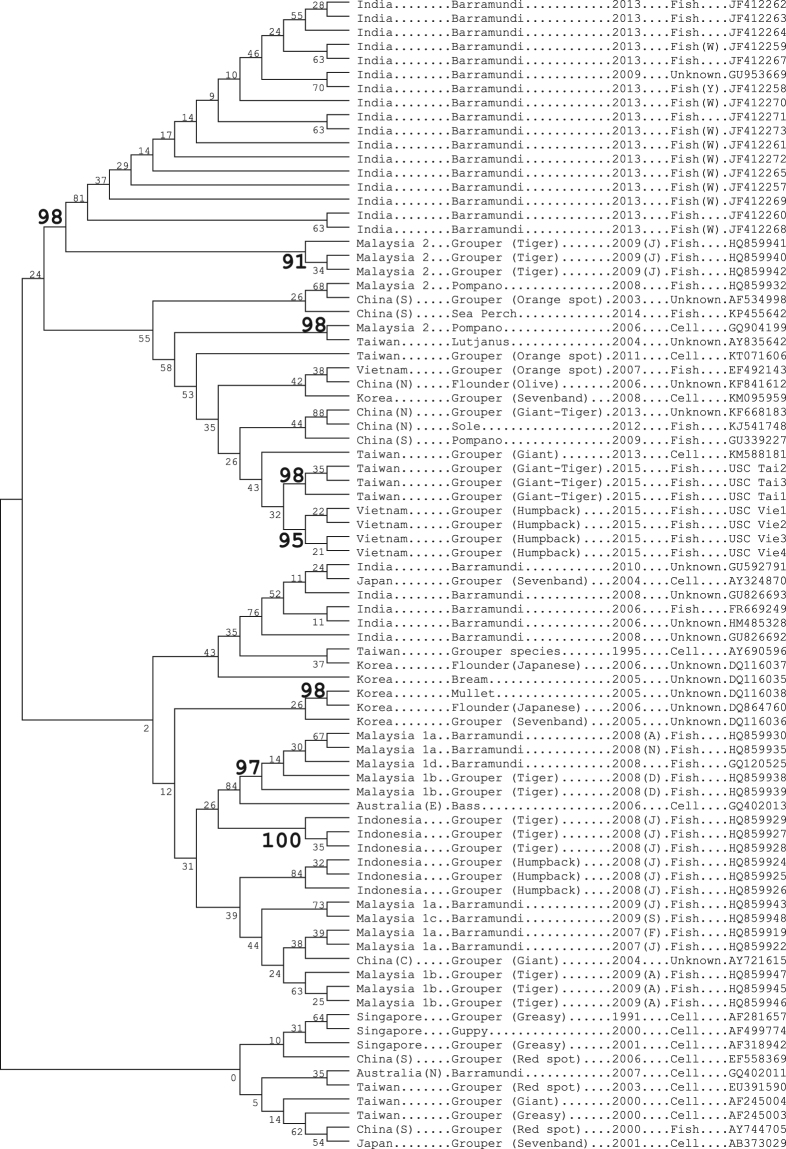
Maximum Likelihood tree for NNV RNA2 RNA sequences. Numbers at tree branches indicate the percentage of replicate trees in which the same clusters are found in the bootstrap tests of 1000 replicates. Letters in brackets after years refer to months of the year. (W) refers to wild fish; (Y) to wild yearling fish.

The maximum likelihood tree (Fig. 2) shows some concordance with the regionality of NNV strains detected by “CATS” (Fig. 1), however the tree analysis revealed far fewer groupings that were statistically significantly different (branch numbers 95 or greater) than previous revealed using individual nucleotides. Confidence of the tree branches (Fig. 2) can be inferred from the number labelled on branches which indicate the proportion of times out of 1000 trials the same node was formed (using Mega 7^24^).

### Potentially confounding factors

#### Effect of cells lines on the data

Considering all samples (Fig. 1) there were 51 samples taken from fish tissue that were sequenced, and 16 samples from cell lines with viruses originally derived from fish, and 15 samples of unknown origin. Because propagation in cell tissue culture may represent new selective environment/s and/or foster the retention of new mutations and cause greater genetic variance, samples from fish were compared with those from cell lines. Analyses of molecular variance showed there was only four percent variation among groups which was not statistically significant. Moreover, similar results were evident in the preceding analyses whether data from cell cultures was included or not.

The average pair wise RNA sequence percent similarity among all the 15 cell culture lines was 97.5% and the most dissimilar pair had 91.5% identity; respective values after removing the anomalous single Australian bass sample were 98.4% and 97.5%; respective values for the grouper only samples were 98.3% and 96.0% (see Supplementary Fig. S3), i.e. after discounting the anomalous Australian bass sample, there was a greater range in sequence variation among the fish samples than among the tissue culture samples considering the whole data set.

#### Sequence diversity within fish and in “outbreaks”

Considering the new RNA sequences generated in this report, each of the RNA2 sequences from three humpback grouper in Vietnam, sampled at the same time from the same hatchery, were all 100% identical. Similarly, all three Taiwanese giant grouper samples sampled at the same time and from the same hatchery were 100% identical (Supplementary Table S2). If there had been another strain, say at 0.5 frequency in both Vietnam and Taiwan, then chance of failing to detect it is less than 2% (even if another strain had been present in both stocks at a frequency of only 30%, the chance of failing to detect in among the six samples is about 10%). Thus it appears unlikely there were different NNV sequences in the infections at moderate frequencies. Apparently similar results, of identical RNA2 genotypes were reported in most other grouper data sets where there were multiple RNA2 sequences for the same species in the same hatchery at the same time, that is, these samples tended to have identical RNA2 genotypes and examples included Humpback grouper in Indonesia, Tiger grouper in Indonesia, Tiger Grouper in East Malaysia and in West Malaysia. Barramundi, however, tended to show slightly different genotypes in the same time/location (e.g. India, Malaysia).

#### Translated protein sequence

**Figure 3:**
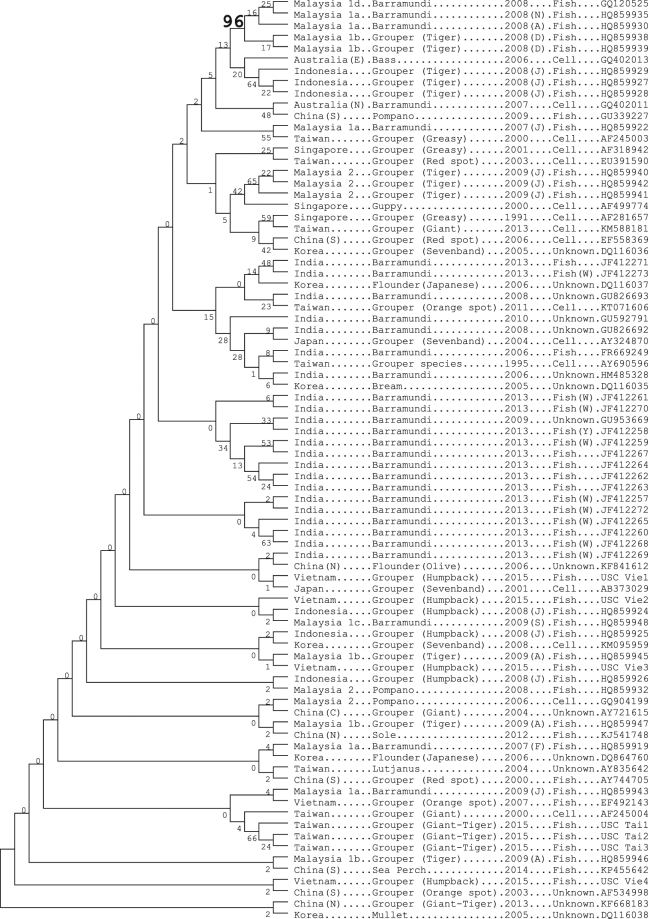
Maximum likelihood tree for NNV RNA2 translated protein sequences. Numbers at tree branches indicate the percentage of replicate trees in which the same clusters are found in the bootstrap tests of 1000 replicates. Letters in brackets after years refer to months of the year. (W) refers to wild fish; (Y) to wild yearling fish.

Of the 50 polymorphic RNA sites tabulated in Fig. 1, 42 were in codon position 3 (RNA polymorphic sites in codon 1 or 2 are in bold, top row, Fig. 1). The average divergence among pairs of samples for protein sequences, considering all 82 samples listed in Fig. 1 was just 1% (Supplementary Fig. S7), and there was little evidence for the pronounced protein “CATS”, as seen for the RNA, that associated with region, species or year. The maximum likelihood tree for the protein sequence (Fig. 3), notwithstanding some regional clustering, did not have significant nodes save for one exception. Codon-based test of purifying selection (MEGA 7^24^) indicated statistically significant (P < 0.001) excess of synonymous substitutions over nonsynonymous substitutions per site when considering all sequences, just those for grouper or just those for barramundi (average numbers of synonymous sites per codon for all samples, grouper and barramundi, respectively, were 1.152, 0.582, 0.317, and respective values for nonsynonymous sites were 0.152, 0.042, 0.089). On the other hand, relative synonymous codon counts for NNV were not statistically significantly different between two major host fish species groups, namely, grouper and barramundi.

## Discussion

### High level of RNA and protein similarity across large regions

From Japan to India, and considering various marine fish hosts, the average sample pair difference for NNV RNA2 cds was just 2% for percent RNA sequence similarity and 1% for the translated protein sequence. At face value, one could infer rather heavy evolutionary constraints on the virus throughout the region and among species, or perhaps co-transport with hosts among regions for commercial production is homogenizing the virus groups. These first impressions are reflected in the literature where previously no formal statistical analysis revealed genetic diversity of Asian RGNNV that significantly associated with region, host or time.

### Characteristic attributes identify regional strains

However, our analyses using “characteristic attributes” and statistical approaches such as AMOVA and n-way ANOVA, and Bayesian Tip-association testing (BaTS) and using the largest data set so far compiled for Asian grouper (and other marine Asian species), show clear regionality in RGNNV strains. This is the first clear evidence of regionality for RGNNV in the Indo Asian region, but interestingly a large survey in Southern Europe by Panzarin *et al*.^28^ also detected regionality (geographic subdivision) of mostly RGNNV strains. This understanding, of regionality for RGNNV strains, is a completely novel one for Asian marine species, and possibly has some implications for selective breeding programs for host resistance in different regions, and also possibly for general management and prevention of this disease. Far more cases of statistically significant regional differences were detected using “characteristic attributes” than using maximum likelihood tree analyses, lending support to the postulate that characteristic attributes can better discriminate closely related species than traditional phylogenetic analyses^16, 18^.

Our evidence for species specific and year specific “CATS” is somewhat less than for regional differences. Actually, there is evidence of sharing of “CAT” genotypes among species, such as grouper and barramundi in Eastern Malaysia and Indonesia, and flounder, grouper and sole in North China (Fig. 1) which questions whether NNV has yet evolved specificity for different marine finfish in Asia. Panzarin *et al*.^28^, in their large survey from Southern Europe, considered many local host species (sea bass, mullet, sea bream etc.) and observed no “species-specific” mutations in the RNA2 region. Our evidence for temporal stability of NNV strains over years is mixed, on one hand, the same “CATS” are evident over six or so years in Northern China, and in Eastern Malaysia over two years, in Vietnam over eight years but not in India over six years (Fig. 1); on balance we cannot conclude that each year brings a new wave of very different genotypes to a particular region, on the contrary, the overall picture is one of genotype stability over years in given regions.

### Potentially confounding effects

There is a possibility that the levels of RNA variation would be artificially increased after passage in cell cultures, because of the different selective pressures in tissue culture than those found in the fish host. Actually, we found no evidence of such a hypothetical effect, and indeed there was slightly more variation in sequences from fish hosts than from cell cultures; moreover AMOVA tests did not show significant differences between the sequences from fish hosts and from cell lines. Accordingly, there does not appear to be any evidence based reason not to include the RNA sequences from cell cultures in our analyses on the grounds that they are intrinsically more variable, or intrinsically more different, than sequences from fish.

Another potentially confounding matter was that of substantial diversity of NNV sequences within hatcheries. We did not find evidence of much if any diversity for the same species, same hatchery and same sampling time for grouper species, although some barramundi samples from India showed some slight differences from the same collection time and place; in any case such slight differences were clearly, *ipso facto*, insufficient to confound the analyses that assessed regional differences (i.e. regional differences were statistically significant).

### RNA genetic diversity is greater than the capsid protein diversity

The RNA sequence considered here was from the RNA2 gene, which encodes the virus protein capsid protein. Interestingly, the translated RNA2 sequences showed even less variation than the RNA with less evidence of region specific amino acid “CATS”; indeed most RNA “CATS” were found at codon position three which did not lead to changes in the capsid protein. Statistical tests indicated a very substantial excess of synonymous substitutions over nonsynonymous sites, indicating “purifying” selection. Every node in the maximum likelihood tree for the protein sequences was not significant for clusters save one. A parsimonious model to account for this interesting discrepancy between the RNA sequence and the protein is that strains to some degree are geographically isolated and free to diverge especially at RNA triplet positions not tightly constrained by selection (i. e. most codon 3 positions), but those sites (RNA codon positions one and two) where changes can lead to changes in the capsid protein, are more tightly constrained by selection. Balancing all the evidence, the null hypothesis is that the regional Asian strains of NNV are functionally equivalent which may give encouragement that selection for resistance in one region will lead to fish capable of resisting NNV in another. Indeed various have reported that all RGNNV strains that were tested in Asian and Europe were serologically related (within each region) and had the same serotype despite different RNA genotypes, further supporting the hypothesis that selection for one RGNNV RNA genotype will give rise to resistance for all within a region^29, 30^. If this hypothesis is confirmed, then variation evident in the RNA sequences and “CATs” may become less relevant to the issue of genetic selection, but still may inform about the epidemiology of the pathogen (see following).

### Implications for disease management

Munday *et al*.^6^ stated “Lack of knowledge of the epidemiology of the diseases caused by nodaviruses, except for vertical transmission of the pathogen in some species, has impeded the development of control measures”. By showing the existence of regional strains of RGNNV in Asian marine fish for the first time, our study points to the existence of regional, endemic sources of NNV that may recurrently infect local hatcheries. This understanding is consistent with reports of finding NNV in wild fish^31^. So in addition to taking precautions not to import NNV along with imported broodstock, grouper hatcheries may also now consider the possibility that NNV can enter the hatcheries from local reservoirs from local wild fish carrying NNV.

## Conclusions

- RNA sequence variation shows the existence of regional Asian RGNN strains
- Translated protein differences showed less regionality than the RNA data
- While gene flow appears largely restricted among regions, selection apparently constrains variability of capsid protein sequence
- The apparent selection constraint mitigates the risk that despite geographic subdivision, NNV strain variability will confound genetic selection for host resistance
- Regional Asian RGNN strains may suggest hatcheries are at risk from NNV not only from imported material but also from endemic reservoirs.

## Electronic supplementary material

Supplementary files and figures
