# Supplementary material for: Regional genetic diversity for NNV grouper viruses across the Indo-Asian region – implications for selecting virus resistance in farmed groupers
Source: Sci Rep. 2017 Sep 6;7:10658. doi: 10.1038/s41598-017-11263-4 (PMC5587679; doi:10.1038/s41598-017-11263-4)
Supplement: Supplementary file 1 — Supplementary files and figures [file 41598_2017_11263_MOESM1_ESM.doc]

**Regional genetic diversity for NNV grouper viruses across the Indo-Asian region – implications for selecting virus resistance in farmed groupers.**

Wayne Knibb^a^ , Giang Luu ^a,b^, H.K.A. Premachandra^a^, Ming-Wei Lu^c^, Nguyen Hong Nguyen^aa^ GeneCology Research Centre, Faculty of Science, Health, Education and Engineering, University of the Sunshine Coast, Maroochydore, QLD 4558, Australia

^b^ Research Institute for Aquaculture No. 1, Dinh Bang, Tu Son, Bac Ninh, Viet Nam

^c^ Department of Aquaculture, National Taiwan Ocean University

Correspondence: Wayne Knibb, Faculty of Science, Health, Education and Engineering, University of the Sunshine Coast, Maroochydore, QLD 4558, Australia (e-mail: [wayne.knibb@gmail.com](mailto:wayne.knibb@gmail.com)).

# Supplementary Figure S1. Testing for presence of NNV in Vietnam and Taiwan

# Supplementary Table S2. cds RNA 2 NNV sequences for 82 samples.

>India........Barramundi............2006....Fish....FR669249

ATGGTACGCAAAGGTGAGAAGAAATTGGCAAAACCCGCGACCACCAAGGCCGCGAATCCGCAACCCCGCCGACGTGCTAACAATCGTCGGCGTAGTAATCGCACTGACGCACCTGTGTCTAAGGCCTCGACTGTAACTGGATTTGGACGTGGGACCAATGACGTCCATCTCTCAGGTATGTCGAGAATCTCCCAGGCCGTCCTCCCAGCCGGGACAGGAACAGACGGATACGTTGTTGTTGACGCAACCATCGTCCCCGACCTCCTGCCACGACTGGGACACGCTGCTAGAATCTTCCAGCGATACGCTGTTGAAACACTGGAGTTTGAAATTCAGCCAATGTGCCCCGCAAACACGGGCGGTGGTTACGTTGCTGGCTTCCTGCCTGATCCAACTGACAACGATCACACCTTCGACGCGCTTCAAGCAACTCGTGGTGCAGTCGTTGCCAAATGGTGGGAAAGCAGAACAGTCCGACCTCAGTACACCCGCACGCTCCTCTGGACCTCGTCGGGAAAGGAGCAGCGTCTCACGTCACCTGGTCGGCTGATACTCCTGTGTGTCGGCAACAACACTGATGTGGTCAACGTGTCAGTGCTGTGTCGCTGGAGTGTTCGACTGAGCGTTCCATCTCTTGAGACACCTGAAGAGACCACCGCTCCCATCATGACACAAGGTTCCCTGTACAACGATTCCCTTTCCACAAATGACTTCAAGTCCATCCTCCTAGGATCCACACCACTGGACATTGCCCCTGATGGAGCAGTCTTCCAGCTGGACCGTCCGCTGTCCATTGACTACAGCCTTGGAACTGGAGATGTTGACCGTGCTGTTTATTGGCACCTCAAGAAGTTTGCTGGAAATGCTGGCACACCTGCAGGCTGGTTTCGCTGGGGCATCTGGGACAACTTCAACAAGACGTTCGCAGATGGCGTTGCCTACTACTCTGATGAGCAGCCTCGTCAAATCCTGCTGCCTGTTGGCACTGTCTGCACTAGGGTTGACTCGGAAAACTAA

>India........Barramundi............2010....Unknown.GU592791

ATGGTACGCAA-GGTGAGAAGAAATTGGCAAAACCCGCGACCACCAAGGCCGCGAATCCGCAACCCCGCCGACGTGCTAACAATCGTCGGCGTAGTAATCGCACTGACGCACCTGTGTCTAAGGCCTCGACTGTAACTGGATTTGGACGTGGGACCAATGACGTCCATCTCTCAGGTATGTCGAGAATCTCCCAGGCCGTCCTCCCAGCCGGGACAGGAACAGACGGATACGTTGTTGTTGACGCAACCATCGTCCCCGACCTCCTGCCACGACTGGGACACGCTGCTAGAATCTTCCAGCGATACGCTGTTGAAACACTGGAGTTTGAAATTCAGCCAATGTGCCCCGCAAACACGGGCGGTGGTTACGTTGCTGGCTTCCTGCCTGATCCAACTGACAACGATCACACCTTCGACGCGCTTCAAGCAACTCGTGGTGCAGTCGTTGCCAAATGGTGGGAAAGCAGAACAGTCCGACCTCAGTACACCCGCACGCTCCTCTGGACCTCGTCGGGAAAGGAGCAGCGTCTCACGTCACCTGGTCGGCTGATACTCCTGTGTGTCGGCAACAACACTGATGTGGTCAACGTGTCAGTGCTGTGTCGCTGGAGTGTTCGACTGAGCGTTCCATCTCTTGAGACACCTGAAGAGACCACCGCTCCCATCATGACACAAGGTTCCCTGTACAACGATTCCCTTTCCACAAATGACTTCAAGTCCATCCTCCTAGGATCCACACCACTGGACATTGCCCCTGATGGAGCAGTCTTCCAGCTGGACCGTCCGCTGTCCATTGACTACAGCCTTGGAACTGGAGATGTTGACCGTGCTGTTTATTGGCACCTCAAGAAGTTTGCTGGAAATGCTGGCACACCTGCAGGCTGGTTTCGCTGGGGCATCTGGGACA-CTTCAACAAGACGTTCGCAGATGCCGTTGGCTACTACTCTGATGCGCAGCCTCGTCAAATCCTGCTGCTGTTGGCCACTGTCTGCACTAGGGTTGACTCGGAAAACTAA

>India........Barramundi............2008....Unknown.GU826692

ATGGTACGCAAAGGTGAGAAGAAATTGGCAAAACCCGCGACCACCAAGGCCGCGAATCCGCAACCCCGCCGACGTGCTAACAATCGTCGGCGTAGTAATCGCACTGACGCACCTGTGTCTAAGGCCTCGACTGTAACTGGATTTGGACGTGGGACCAATGACGTCCATCTCTCAGGTATGTCGAGAATCTCCCAGGCCGTCCTCCCAGCCGGGACAGGAACAGACGGATACGTTGTTGTTGACGCAACCATCGTCCCCGACCTCCTGCCACGACTGGGACACGCTGCTAGAATCTTCCAGCGATACGCTGTTGAAACACTGGAGTTTGAAATTCAGCCAATGTGCCCCGCAAACACGGGCGGTGGTTACGTTGCTGGCTTCCTGCCTGATCCAACTGACAACGATCACACCTTCGACGCGCTTCAAGCAACTCGTGGTGCAGTCGTTGCCAAATGGTGGGAAAGCAGAACAGTCCGACCTCAGTACACTCGCACGCTCCTCTGGACCTCGTCGGGAAAGGAGCAGCGTCTCACGTCACCTGGTCGGCTGATACTCCTGTGTGTCGGCAACAACACTGATGTGGTCAACGTGTCAGTGCTGTGTCGCTGGAGTGTTCGACTGAGCGTTCCATCTCTTGAGACACCTGAAGAGACCACCGCTCCCATCATGACACAAGGTTCCCTGTACAACGATTCCCTTTCCACAAATGACTTCAAGTCCATCCTCCTAGGATCCACACCACTGGACATTGCCCCTGATGGAGCAGTCTTCCAGCTGGACCGTCCGCTGTCCATTGACTACAGCCTTGGAACTGGAGATGTTGACCGTGCTGTTTATTGGCACCTCAAGAAGTTTGCTGGAAATGCTGGCACACCTGCAGGCTGGTTTCGCTGGGGCATCTGGGACAACTTCAACAAGACGTTCGCAGATGGCGTTGCCTACTACTCTGATGAGCAGCCTCGTCAAATCCTGCTGCCTGTTGGCACTGTCTGCACTAGGGTTGACTCGGAAAACTAA

>India........Barramundi............2008....Unknown.GU826693

ATGGTACGCAAAGGTGAGAAGAAATTGGCAAAACTCGCGACCACCAAGGCCGCGAATCCGCAACCCCGCCGACGTGCTAACAATCGTCGGCGTAGTAATCGCACTGACGCACCTGTGTCTAAGGCCTCGACTGTAACTGGATTTGGACGTGGGACCAATGGCGTCCATCTCTCAGGTATGTCGAGAATCTCCCAGGCCGTCCTCCCAGCCGGGACAGGAACAGACGGATACGTTGTTGTTGACGCAACCATCGTCCCCGACCTCCTGCCACGACTGGGACACGCTGCTAGAATCTTCCAGCGATACGCTGTTGAAACACTGGAGTTTGAAATTCAGCCAATGTGCCCCGCAAACACGGGCGGTGGTTACGTTGCTGGCTTCCTGCCTGATCCAACTGACAACGATCACACCTTCGACGCGCTTCAAGCAACTCGTGGTGCAGTCGTTGCCAAATGGTGGGAAAGCAGAACAGTCCGACCTCAGTACACCCGCACGCTCCTCTGGACCTCGTCGGGAAAGGAGCAGCGTCTCACGTCACCTGGTCGGCTGATACTCCTGTGTGTCGGCAACAACACTGATGTGGTCAACGTGTCAGTGCTGTGTCGCTGGAGTGTTCGACTGAGCGTTCCATCCCTTGAGACACCTGAAGAGACCACCGCTCCCATCATGACACAAGGTTCCCTGTACAACGATTCCCTTTCCACAAATGACTTCAAGTCCATCCTCCTAGGATCCACACCACTGGACATTGCCCCTGATGGAGCAGTCTTCCAGCTGGACCGTCCGCTGTCCATTGACTACAGCCTTGGAACTGGAGATGTTGACCGTGCTGTTTATTGGCACCTCAAGAAGTTTGCTGGAAATGCTGGCACACCTGCAGGCTGGTTTCGCTGGGGCATCTGGGACAACTTCAACAAGACGTTCGCAGATGGCGTTGCCTACTACTCTGATGAGCAGCCTCGTCAAATCCTGCTGCCTGTTGGCACTGTCTGCACTAGGGTTGACTCGGAAAACTAA

>India........Barramundi............2009....Unknown.GU953669

ATGGTACGCAAAGGTGAGAAGAAATTGGCAAAACCCGCGACCACCAAGGCCGCGAATCCGCAACCCCGCCGACGTGCTAACAATCGTCGGCGTAGTAATCGCACTGACGCACCTGTGTCAAAGGCCTCGACTGTGACTGGATTTGGACGTGGGACCAATGACGTCCATCTCTCAGGTATGTCGAGAATCTCCCAGGCCGTCCTCCCAGCCGGGACAGGAACAGACGGATACGTTGTTGTTGACGCAACCATCGTCCCCGACCTCCTGCCACGACTGGGACACGCTGCTAGAATCTTCCAGCGATACGCTGTTGAAACACTGGAGTTTGAAATTCAGCCAATGTGCCCCGCAAACACAGGCGGTGGTTACGTTGTTGGCTTCCTGCCTGATCCAACTGACAACGACCACACCTTCGATGCGCTTCAAGCAACTCGTGGTGCAGTCGTTGCCAAATGGTGGGAAAGCAGAACAGTCCGACCTCAGTACACCCGCACGCTCCTCTGGACCTCGTCGGGAAAGGAGCAGCGTCTCACGTCACCTGGTCGGCTGATACTCCTGTGTGTCGGCAACAACACTGATGTGGTCAACGTGTCAGTACTGTGTCGCTGGAGTGTTCGACTGAGCGTTCCATCTCTTGAGACACCTGAGGAGACAACCGCTCCCATCATGACACAAGGTTCCCTGTACAACGATTCCCTTTCCACAAATGACTTCAAGTCCATCCTCCTAGGATCCACACCACTGGACATTGCCCCTGATGGAGCAGTCTTCCAGCTGGACCGTCCGCTGTCCATTGACTACAGCCTTGGAACTGGAGATGTTGATCGAGCTGTCTATTGGCACCTCAAGAAGTTTGCTGGAAATGCCGGCACACCTGCAGGCTGGTTTCGCTGGGGCATCTGGGACAACTTCAACAAGACGTTCACAGATGGCGTTGCCTACTACTCTGATGAGCAGCCCCGTCAAATCCTGCTGCCTGTTGGCACTGTCTGCACCAGGGTTGACTCGGAAAACTAA

>India........Barramundi............2006....Unknown.HM485328

ATGGTACGCAAAGGTGAGAAGAAATTGGCAAAACCCGCGACCACCAAGGCCGCGAATCCGCAACCCCGCCGACGTGCTAACAATCGTCGGCGTAGTAATCGCACTGACGCACCTGTGTCTAAGGCCTCGACTGTAACTGGATTTGGACGTGGGACCAATGACGTCCATCTCTCAGGTATGTCGAGAATCTCCCAGGCCGTCCTCCCAGCCGGGACAGGAACAGACGGATACGTTGTTGTTGACGCAACCATCGTCCCCGACCTCCTGCCACGACTGGGACACGCTGCTAGAATCTTCCAGCGATACGCTGTTGAAACACTGGAGTTTGAAATTCAGCCAATGTGCCCCGCAAACACGGGCGGTGGTTACGTTGCTGGCTTCCTGCCTGATCCAACTGACAACGATCACACCTTCGACGCGCTTCAAGCAACTCGTGGTGCAGTCGTTGCCAAATGGTGGGAAAGCAGAACAGTCCGACCTCAGTACACCCGCACGCTCCTCTGGACCTCGTCGGGAAAGGAGCAGCGTCTCACGTCACCTGGTCGGCTGATACTCCTGTGTGTCGGCAACAACACTGATGTGGTCAACGTGTCAGTGCTGTGTCGCTGGAGTGTTCGACTGAGCGTTCCATCTCTTGAGACACCTGAAGAGACCACCGCTCCCATCATGACACAAGGTTCCCTGTACAACGATTCCCTTTCCACAAATGACTTCAAGTCCATCCTCCTAGGATCCACACCACTGGACATTGCCCCTGATGGAGCAGTCTTCCAGCTGGACCGTCCGCTGTCCATTGACTACAGCCTTGGAACTGGAGATGTTGACCGTGCTGTTTATTGGCACCTCAAGAAGTTTGCTGGAAATGCTGGCACACCTGCAGGCTGGTTTCGCTGGGGCATCTGGGACAACTTCAACAAGACGTTCGCAGATGGCGTTGCCTACTACTCTGATGAGCAGCCTCGTCAAATCCTGCTGCCTGTTGGCACTGTCTGCACTAGGGTTGACTCGGAAAACTAA

>India........Barramundi............2013....Fish(W).JF412257

ATGGTACGCAAAGGTGAGAAGAAATTGGCAAAACCCGCGACCACCAAGGCCGCGAATCCGCAACCCCGCCGACGTGCTAACAATCGTCGGCATAGTAATCGCACTGACGCACCTGTGTCAAAGGCCTCGACTGTGACTGGATTTGGACGTGGGACCAATGACGTCCATCTCTCAGGTATGTCGAGAATCTCCCAGGCCGTCCTCCCAGCCGGGACAGGAACAGACGGATACGTTGTTGTTGACGCAACCATCGTCCCCGACCTCCTGCCACGACTGGGACACGCTGCTAGAATCTTCCAGCGATACGCTGTTGAAACACTGGAGTTTGAAATTCAGCCAATGTGCCCCGCAAACACGGGCGGTGGTTACGTTGCTGGCTTCCTGCCTGATCCAACTGACAACGACCACACCTTCGATGCGCTTCAAGCAACTCGTGGTGCAGTCGTTGCCAAATGGTGGGAAAGCAGAACAGTCCGACCTCAGTACACCCGCACGCTCCTCTGGACCTCGTCGGGAAAGGAGCAGCGTCTCACGTCACCTGGTCGGCTGATACTCCTGTGTGTCGGCAACAACACTGATGTGGTCAACGTGTCAGTACTGTGTCGCTGGAGTGTTCGACTGAGCGTTCCATCTCTTGAGACACCTGAGGAGACAACCGCTCCCATCATGACACAAGGTTCCCTGTACAACGATTCCCTTTCCACAAATGACTTCAAGTCCATCCTCCTAGGATCCACACCACTGGACATTGCCCCTGATGGAGCAGTCTTCCAGCTGGACCGTCCGCTGTCCATTGACTACAGCCTTGGAACTGGAGATGTTGATCGAGCTGTCTATTGGCACCTCAAGAAGTTTGCTGGAAATGCTGGCACACCTGCAGGCTGGTTTCGCTGGGGCATCTGGGACAACTTCAACAAGACGTTCACAGATGGCGTTGCCTACTACTCTGATGAGCAGCCCCGTCAAATCCTGCTGCCTGTTGGCACTGTCTGCACCAGGGTTGACTCGGAAAACTAA

>India........Barramundi............2013....Fish(Y).JF412258

ATGGTACGCAAAGGTGAGAAGAAATTGGCAAAACCCGCGACCACCAAGGCCGCGAATCCGCAACCCCGCCGACGTGCTAACAATCGTCGGCGTAGTAATCGCACTGACGCACCTGTGTCAAAGGCCTCGACTGTGACTGGATTTGGACGTGGGACCAATGACGTCCATCTCTCAGGTATGTCGAGAATCTCCCAGGCCGTCCTCCCAGCCGGGACAGGAACAGACGGATACGTTGTTGTTGACGCAACCATCGTCCCCGACCTCCTGCCACGACTGGGACACGCTGCTAGAATCTTCCAGCGATACGCTGTTGAAACACTGGAGTTTGAAATTCAGCCAATGTGCCCCGCAAACACAGGCGGTGGTTACGTTGTTGGCTTCCTGCCTGATCCAACTGACAACGACCACACCTTCGATGCGCTTCAAGCAACTCGTGGTGCAGTCGTTGCCAAATGGTGGGAAAGCAGAACAGTCCGACCTCAGTACACCCGCACGCTCCTCTGGACCTCGTCGGGAAAGGAGCAGCGTCTCACGTCACCTGGTCGGCTGATACTCCTGTGTGTCGGCAACAACACTGATGTGGTCAACGTGTCAGTACTGTGTCGCTGGAGTGTTCGACTGAGCGTTCCATCTCTTGAGACACCTGAGGAGACAACCGCTCCCATCATGACACAAGGTTCCCTGTACAACGATTCCCTTTCCACAAATGACTTCAAGTCCATCCTCCTAGGATCCACACCACTGGACATTGCCCCTGATGGAGCAGTCTTCCAGCTGGACCGTCCGCTGTCCATTGACTACAGCCTTGGAACTGGAGATGTTGATCGAGCTGTCTATGGGCACCTCAAGAAGTTTGCTGGAAATGCCGGCACACCTGCAGGCTGGTTTCGCTGGGCCATCTGGGACAACTTCAACAAGACGTTCACAGATGGCGTTGCCTACTACTCTGATGAGCAGCCCCGTCAAATCCTGCTGCCTGTTGGCACTGTCTGCACCAGGGTTGACTCGGAAAACTAA

>India........Barramundi............2013....Fish(W).JF412259

ATGGTACGCAAAGGTGAGAAGAAATTGGCAAAACCCGCGACCGCCAAGGCCGCGAATCCGCAACCCCGCCGACGTGCTAACAATCGTCGGCGTAGTAATCGCACTGACGCACCTGTGTCAAAGGCCTCGACTGTGACTGGATTTGGACGTGGGACCAATGACGTCCATCTCTCAGGTATGTCGAGAATCTCCCAGGACGTCCTCCCAGCCGGGACAGGAACAGACGGATACGTTGTTGTTGACGCAACCATCGTCCCCGACCTCCTGCCACGACTGGGACACGCTGCTAGAATCTTCCAGCGATACGCTGTTGAAACACTGGAGTTTGAAATTCAGCCAATGTGCCCCGCAAACACGGGCGGTGGTTACGTTGCTGGCTTCCTGCCTGATCCAACTGACAACGACCACACCTTCGATGCGCTTCAAGCAACTCGTGGTGCAGTCGTTGCCAAATGGTGGGAAAGCAGAACAGTCCGACCTCAGTACACCCGCACGCTCCTCTGGACCTCGTCGGGAAAGGAGCAGCGTCTCACGTCACCTGGTCGGCTGATACTCCTGTGTGTCGGCAACAACACTGATGTGGTCAACGTGTCAGTACTGTGTCGCTGGAGTGTTCGACTGAGCGTTCCATCTCTTGAGACACCTGAGGAGACAACCGCTCCCATCATGACACAAGGTTCCCTGTACAACGATTCCCTTTCCACAAATGACTTCAAGTCCATCCTCCTAGGATCCACACCACTGGACATTGCCCCTGATGGAGCAGTCTTCCAGCTGGACCGTCCGCTGTCCATTGACTACAGCCTTGGAACTGGAGATGTTGATCGAGCTGTCTATGGGCACCTCAAGAAGTTTGCTGGAAATGCTGGCACACCTGCAGGCTGGTTTCGCTGGGCCATCTGGGACAACTTCAACAAGACGTTCACAGATGGCGTTGCCTACTACTCTGATGAGCAGCCCCGTCAAATCCTGCTGCCTGTTGGCACTGTCTGCACCAGGGTTGACTCGGAAAACTAA

>India........Barramundi............2013....Fish....JF412260

ATGGTACGCAAAGGTGAGAAGAAATTGGCAAAACCCGCGACCACCAGGGCCGCGAATCCGCAACCCCGCCGACGTGCTAACAATCGTCGGCGTAGTAATCGCACTGACGCACCTGTGTCAAAGGCCTCGACTGTGACTGGATTTGGACGTGGGACCAATGACGTCCATCTCTCAGGTATGTCGAGAATCTCCCAGGCCGTCCTCCCAGCCGGGACAGGAACAGACGGATACGTTGTTGTTGACGCAACCATCGTCCCCGACCTCCTGCCACGACTGGGACACGCTGCTAGAATCTTCCAGCGATACGCTGTTGAAACACTGGAGTTTGAAATTCAGCCAATGTGCCCCGCAAACACGGGCGGTGGTTACGTTGCTGGCTTCCTGCCTGATCCAACTGACAACGACCACACCTTCGATGCGCTTCAAGCAACTCGTGGTGCAGTCGTTGCCAAATGGTGGGAAAGCAGAACAGTCCGACCTCAGTACACCCGCACGCTCCTCTGGACCTCGTCGGGAAAGGAGCAGCGTCTCACGTCACCTGGTCGGCTGATACTCCTGTGTGTCGGCAACAACACTGATGTGGTCAACGTGTCAGTACTGTGTCGCTGGAGTGTTCGACTGAGCGTTCCATCTCTTGAGACACCTGAGGAGACAACCGCTCCCATCATGACACAAGGTTCCCTGTACAACGATTCCCTTTCCACAAATGACTTCAAGTCCATCCTCCTAGGATCCACACCACTGGACATTGCCCCTGATGGAGCAGTCTTCCAGCTGGACCGTCCGCTGTCCATTGACTACAGCCTTGGAACTGGAGATGTTGATCGAGCTGTCTATTGGCACCTCAAGAAGTTTGCTGGAAATGCTGGCACACCTGCAGGCTGGTTTCGCTGGGGCATCTGGGACAACTTCAACAAGACGTTCACAGATGGCGTTGCCTACTACTCTGATGAGCAGCCCCGTCAAATCCTGCTGCCTGTTGGCACTGTCTGCACCAGGGTTGACTCGGAAAACTAA

>India........Barramundi............2013....Fish(W).JF412261

ATGGTACGCAAAGGTGAGAAGAAATTGGCAAAACCCGCGACCACCAAGGCCGCGAATCCGCAACCCCGCCGACGTGCTAACAATCGTCGGCGTAGTAATCGCACTGACGCACCTGTGTCAAAGGCCTCGACTGTGACTGGATTTGGACGTGGGACCAATGACGTCCATCTCTCAGGTATGTCGAGAATCTCCAAGGCCGTCCTCCCAGCCGGGACAGGAACAGACGGATACGTTGTTGTTGACGCAACCATCGTCCCCGACCTCCTGCCACGACTGGGACACGCTGCTAGAATCTTCCAGCGATACGCTGTTGAAACACTGGAGTTTGAAATTCAGCCAATGTGCCCCGCAAACACGGGCGGTGGTTACGTTGCTGGCTTCCTGCCTGATCCAACTGACAACGACCACACCTTCGATGCGCTTCAAGCAACTCGTGGTGCAGTCGTTGCCAAATGGTGGGAAAGCAGAACAGTCCGACCTCAGTACACCCGCACGCCCCTCTGGACCTCGTCGGGAAAGGAGCAGCGTCTCACGTCACCTGGTCGGCTGATACTCCTGTGTGTCGGCAACAACACTGATGTGGTCAACGTGTCAGTACTGTGTCGCTGGAGTGTTCGACTGAGCGTTCCATCTCTTGAGACACCTGAGGAGACAACCGCTCCCATCATGACACAAGGTTCCCTGTACAACGATTCCCTTTCCACAAATGACTTCAAGTCCATCCTCCTAGGATCCACACCACTGGACATCGCCCCTGATGGAGCAGTCTTCCAGCTGGACCGTCCGCTGTCCATTGACTACAGCCTTGGAACTGGAGATGTTGATCGAGCTGTCTATTGGCACCTCAAGAAGTTTGCTGGAAATGCTGGCACACCTGCAGGCTGGTTTCGCTGGGGCATCTGGGACAACTTCAACAAGACGTTCACAGATGGCGTTGCCTACTACTCTGATGAGCAGCCCCGTCAAATCCTGCTGCCTGTTGGCACTGTCTGCACCAGGGTTGACTCGGAAAACTAA

>India........Barramundi............2013....Fish....JF412262

ATGGTACGCAAAGGTGAGAAGAAATTGGCAAAACCCGCGACCACCAAGGCCGCGAATCCGCAACCCCGCCGACGTGCTAACAATCGTCGGCGTAGTAATCGCATTGACGCACCTGTGTCAAAGGCCTCGACTGTGACTGGATTTGGACGTGGGACCAATGACGTCCATCTCTCAGGTATGTCGAGAATCTCCCAAGCCGTCCTCCCAGCCGGGACAGGAACAGACGGATACGTTGTTGTTGACGCAACCATCGTCCCCGACCTCCTGCCACGACTGGGACACGCTGCTAGAATCTTCCAGCGATACGCTGTTGAAACACTGGAGTTTGAAATTCAGCCAATGTGCCCCGCAAACACGGGCGGTGGTTACGTTGCTGGCTTCCTGCCCGATCCAACTGACAACGACCACACCTTCGATGCGCTTCAAGCAACTCGTGGTGCAGTCGTTGCCAAATGGTGGGAAAGCAGAACAGTCCGACCTCAGTACACCCGCACGCTCCTCTGGACCTCGTCGGGAAAGGAGCAGCGTCTCACGTCACCTGGTCGGCTGATACTCCTGTGTGTCGGCAACAACACTGATGTGGTCAACGTGTCAGTACTGTGTCGCTGGAGTGTTCGACTGAGCGTTCCATCTCTTGAGACACCTGAGGAGACAACCGCTCCCATCATGACACAAGGTTCCCTGTACAACGATTCCCTTTCCACAAATGACTTCAAGTCCATCCTCCTAGGATCCACACCACTGGACATTGCCCCTGATGGAGCAGTCTTCCAGCTGGACCGTCCGCTGTCCATTGACTACAGCCTTGGAACTGGAGATGTTGATCGAGCTGTCTATGGGCACCTCAAGAAGTTTGCTGGAAATGCTGGCACACCTGCAGGCTGGTTTCGCTGGGGCATCTGGGACAACTTCAACAAGACGTTCACAGATGGCGTTGCCTACTACTCTGATGAGCAGCCCCGTCAAATCCTGCTGCCTGTTGGCACTGTCTGCACCAGGGTTGACTCGGAAAACTAA

>India........Barramundi............2013....Fish....JF412263

ATGGTACGCAAAGGTGAGAAGAAATTGGCAAAACCCGCGACCACCAAGGCCGCGAATCCGCAACCCCGCCGACGAGCTAACAATCGTCGGCGTAGTAATCGCACTGACGCACCTGTGTCAAGGGCCTCGACTGTGACTGGATTTGGACGTGGGACCAATGACGTCCATCTCTCAGGTATGTCGAGAATCCCCCAGGCCGTCCTCCCAGCCGGGACAGGAACAGACGGATACGTTGTTGTTGACGCAACCATCGTCCCCGACCTCCTGCCACGACTGGGACACGCCGCTAGAATCTTCCAGCGATACGCTGTTGAAACACTGGAGTTTGAAATCCAGCCAATGTGCCCCGCAAACACGGGCGGTGGTTACGTTGCTGGCTTCCTGCCTGATCCAACTGACAACGACCACACCTTCGATGCGCTTCAAGCAACTCGTGGTGCAGTCGTTGCCAAATGGTGGGAAAGCAGAACAGTCCGACCTCAGTACACCCGCACGCTCCTCTGGACCTCGTCGGGAAAGGAGCAGCGTCTCACGTCACCTGGTCGGCTGATACTCCTGTGTGTCGGCAACAACACTGATGTGGTCAACGTGTCAGTACTGTGTCGCTGGAGTGTTCGACTGAGCGTTCCATCTCTTGAGACACCTGAGGAGACAACCGCTCCCATCATGACACAAGGTTCCCTGTACAACGATTCCCTTTCCACAAATGACTTCAAGTCCATCCTCCTAGGATCCACACCACTGGACATTGCCCCTGATGGAGCAGTCTTCCAGCTGGACCGTCCGCTGTCCATTGACTACAGCCTTGGAACTGGAGATGTTGATCGAGCTGTCTATGGGCACCTCAAGAAGTTTGCTGGAAATGCTGGCACACCTGCAGGCTGGTTTCGCTGGGGCATCTGGGACAACTTCAACAAGACGTTCACAGATGGCGTTGCCTACTACTCTGATGAGCAGCCCCGTCAAATCCTGCTGCCTGTTGGCACTGTCTGCACCAGGGTTGACTCGGAAAACTAA

>India........Barramundi............2013....Fish....JF412264

ATGGTACGCAAAGGTGAGAAGAAATTGGCAAAACCCGCGACCACCAAGGCCGCGAATCCGCAACCCCGCCGACGTGCTAACAATCGTCGGCGTAGTAATCGCACTGACGCACCTGTGTCAAAGGCCTCGACTGTGACTGGATTTGGACGTGGGACCAATGACGTCCATCTCTCAGGTATGTCGAGAATCTCCCAGGCCGTCCTCCCAGCCGGGACAGGAACAGACGGATACGTTGTTGTTGACGCAACCATCGTCCCCGACCTCCTGCCACGACTGGGACACGCTGCTAGAATCTTCCAGCGATACGCTGTTGAAACACTGGAGTTTGAAATTCAGCCAATGTGCCCCGCAAACACGGGCGGTGGTTACGTTGCTGGCTTCCTGCCTGATCCAACTGACAACGACCACACCTTCGATGCGCTTCAAGCAACTCGGGGTGCAGTCGTTGCCAAATGGTTGGAAAGCAGAACAGTCCGACCTCAGTACACCCGCACGCTCCTCTGGACCTCGTCGGGAAAGGAGCAGCGTCTCACGTCACCTGGTCGGCTGATACTCCTGTGTGTCGGCAACAACACTGATGTGGTCAACGTGTCAGTACTGTGTCGCTGGAGTGTTCGACTGAGCGTTCCATCTCTTGAGACACCTGAGGAGACAACCGCTCCCATCATGACACAAGGTTCCCTGTACAACGATTCCCTTTCCACAAATGACTTCAAGTCCATCCTCCTAGGATCCACACCACTGGACATTGCCCCTGATGGAGCAGTCTTCCAGCTGGACCGTCCGCTGTCCATTGACTACAGCCTTGGAACTGGAGATGTTGATCGAGCTGTCTATGGGCACCTCAAGAAGTTTGCTGGAAATGCTGGCACACCTGCAGGCTGGTTTCGCTGGGGCATCTGGGACAACTTCAACAAGACGTTCACAGATGGCGTTGCCTACTACTCTGATGAGCAGCCCCGTCAAATCCTGCTGCCTGTTGGCACTGTCTGCACCAGGGTTGACTCGGAAAACTAA

>India........Barramundi............2013....Fish(W).JF412265

ATGGTACGCAAAGGTGAGAAGAAATTGGCAAAACCCGCGACCACCAAGGCCGCGAATCCGCAACCCCGCCGACGTGCTAACAATCGTCGGCGTAGTAATCGCACTGACGCACCTGTGTCAAAGGCCTCGACTGTGACTGGATTTGGACGTGGGACCAATGACGTCCATCTCTCAGGTATGTCGAGAATCTCCCAGGCCGTCCTCCCAGCCGGGACAGGAACAGACGGATACGTTGTTGTTGACGCAACCATCGTCCCCGACCTCCTGCCACGACTGGGACACGCTGCTAGAATCTTCCAGCGATACGCTGTTGAAACACTGGAGTTTGAAATTCGGCCAATGTGCCCCGCAAACACGGGCGGTGGTTACGTTGCTGGCTTCCTGCCTGATCCAACTGACAACGACCACACCTTCGATGCGCTTCAAGCAACTCGTGGTGCAGTCGTTGCCAAATGGTGGGAAAGCAGAACAGTCCGACCTCAGTACACCCGCACGCTCCTCTGGACCTCGTCGGGAAAGGAGCAGCGTCTCACGTCACCTGGTCGGCTGATACTCCTGTGTGTCGGCAACAACACTGATGTGGTCAACGTGTCAGTACTGTGTCGCTGGAGTGTTCGACTGAGCGTTCCATCTCTTGAGACACCTGAGGAGACAACCGCTCCCATCATGACACAAGGTTCCCTGTACAACGATTCCCTTTCCACAAATGACTTCAAGTCCATCCTCCTAGGATCCACACCACTGGACATTGCCCCTGATGGAGCAGTCTTCCAGCTGGACCGTCCGCTGTCCATTGACTACAGCCTTGGAACTGGAGATGTTGATCGAGCTGTCTATTGGCACCTCAAGAAGTTTGCTGGAAATGCTGGCACACCTGCAGGCTGGTTTCGCTGGGGCATCTGGGACAACTTCAACAAGACGTTCACAGATGGCGTTGCCTACTACTCTGATGAGCAGCCCCGTCAAATCCTGCTGCCTGTTGGCACTGTCTGCACCAGGGTTGACTCGGAAAACTAA

>India........Barramundi............2013....Fish....JF412267

ATGGTACGCAAAGGTGAGAAGAAATTGGCAAAACCCGCGACCGCCAAGGCCGCGAATCCGCAACCCCGCCGACGTGCTAACAATCGTCGGCGTAGTAATCGCACTGACGCACCTGTGTCAAAGGCCTCGACTGTGACTGGATTTGGACGTGGGACCAATGACGTCCATCTCTCAGGTATGTCGAGAATCTCCCAGGACGTCCTCCCAGCCGGGACAGGAACAGACGGATACGTTGTTGTTGACGCAACCATCGTCCCCGACCTCCTGCCACGACTGGGACACGCTGCTAGAATCTTCCAGCGATACGCTGTTGAAACACTGGAGTTTGAAATTCAGCCAATGTGCCCCGCAAACACGGGCGGTGGTTACGTTGCTGGCTTCCTGCCTGATCCAACTGACAACGACCACACCTTCGATGCGCTTCAAGCAACTCGTGGTGCAGTCGTTGCCAAATGGTGGGAAAGCAGAACAGTCGGACCTCAGTACACCCGCACGCTCCTCTGGACCTCGTCGAGAAAGAAGCAGCGTCTCACGTCACCTGGTCGGCTGATACTCCTGTGTGTCGGCAACAACACTGATGTGGTCAACGTGTCAGTACTGTGTCGCTGGAGTGTTCGACTGAGCGTTCCATCTCTTGAGACACCTGAGGAGACAACCGCTCCCATCATGACACAAGGTTCCCTGTACAACGATTCCCTTTCCACAAATGACTTCAAGTCCATCCTCCTAGGATCCACACCACTGGACATTGCCCCTGATGGAGCAGTCTTCCTGCTGGACCGTCCGCTGTCCATTGACTACAGCCTTGGAACTGGAGATGTTGATCGAGCTGTCTATTGGCACCTCAAGAAGTTTGCTGGAAATGCTGGCACACCTGCAGGCTGGTTTCGCTGGGGCATCTGGGACAACTTCAACAAGACGTTCACAGATGGCGTTGCCTACTACTCTGATGAGCAGCCCCGTCAAATCCTGCTGCCTGTTGGCACTGTCTGCACCAGGGTTGACTCGGAAAACTAA

>India........Barramundi............2013....Fish(W).JF412268

ATGGTACGCAAAGGTGAGAAGAAATTGGCAAAACCCGCGACCACCAGGGCCGCGAATCCGCAACCCCGCCGACGTGCTAACAATCGTCGGCGTAGTAATCGCACTGACGCACCTGTGTCAAAGGCCTCGACTGTGACTGGATTTGGACGTGGGACCAATGACGTCCATCTCTCAGGTATGTCGAGAATCTCCCAGGCCGTCCTCCCAGCCGGGACAGGAACAGACGGATACGTTGTTGTTGACGCAACCATCGTCCCCGACCTCCTGCCACGACTGGGACACGCTGCTAGAATCTTCCAGCGATACGCTGTTGAAACACTGGAGTTTGAAATTCAGCCAATGTGCCCCGCAAACACGGGCGGTGGTTACGTTGCTGGCTTCCTGCCTGATCCAACTGACAACGACCACACCTTCGATGCGCTTCAAGCAACTCGTGGTGCAGTCGTTGCCAAATGGTGGGAAAGCAGAACAGTCCGACCTCAGTACACCCGCACGCTCCTCTGGACCTCGTCGGGAAAGGAGCAGCGTCTCACGTCACCTGGTCGGCTGATACTCCTGTGTGTCGGCAACAACACTGATGTGGTCAACGTGTCAGTACTGTGTCGCTGGAGTGTTCGACTGAGCGTTCCATCTCTTGAGACACCTGAGGAGACAACCGCTCCCATCATGACACAAGGTTCCCTGTACAACGATTCCCTTTCCACAAATGACTTCAAGTCCATCCTCCTAGGATCCACACCACTGGACATTGCCCCTGATGGAGCAGTCTTCCAGCTGGACCGTCCGCTGTCCATTGACTACAGCCTTGGAACTGGAGATGTTGATCGGGCTGTCTATTGGCACCTCAGGAAGTTTGCTGGAAATGCTGGCACACCTGCAGGCTGGTTTCGCTGGGGCATCTGGGACAACTTCAACAAGACGTTCACAGATGGCGTTGCCTACTACTCTGATGAGCAGCCCCGTCAAATCCTGCTGCCTGTTGGCACTGTCTGCACCAGGGTTGACTCGGAAAACTAA

>India........Barramundi............2013....Fish(W).JF412269

ATGGTACGCAAAGGTGAGAAGAAGTTGGCAAAACCCGCGACCACCAAGGCCGCGAATCCCCAACCCCGCCGACGTGCTAACAATCGTCGGCGTAGTAATCGCACTGACGCACCTGTGTCAAAGGCCTCGACTGTGACTGGATTTGGACGTGGGACCAATGACGTCCATCTCTCAGGTATGTCGAGAATCTCCCAGGCCGTCCTCCCAGCCGGGACAGGAACAGACGGATACGTTGTTGTTGACGCAACCATCGTCCCCGACCTCCTGCCACGACTGGGACACGCTGCTAGAATCTTCCAGCGATACGCTGTTGAAACACTGGAGTTTGAAATTCAGCCAATGTGCCCCGCAAACACGGGCGGTGGTTACGTTGCTGGCTTCCTGCCTGATCCAACTGACAACGACCACACCTTCGATGCGCTTCAAGCAACTCGTGGTGCAGTCGTTGCCAAATGGTGGGAAAGCAGAACAGTCCGACCTCAGTACACCCGCACGCTCCTCTGGACCTCGTCGGGAAAGGAGCAGCGTCTCACGTCACCTGGTCGGCTGATACTCCTGTGTGTCGGCAACAACACTGATGTGGTCAACGTGTCAGTACTGTGTCGCTGGAGTGTTCGACTGAGCGTTCCATCTCTTGAGACACCTGAGGAGACAACCGCTCCCATCATGACACAAGGTTCCCTGTACAACGATTCCCTTTCCACAAATGACTTCAAGTCCATCCTCCTAGGATCCACACCACTGGACATTGCCCCTGATGGAGCAGTCTTCCAGCTGGACCGTCCGCTGTCCATTGACTACAGCCTTGGAACTGGAGATGTTGATCGAGCTGTCTATTGGCACCTCAAGAAGTTTGCTGGAAATGCTGGCACACCTGCAGGCTGGTTTCGCTGGGGCATCTGGGACAACTTCAACAAGACGTTCACAGATGGCGTTGCCTACTACTCTGATGAGCAGCCCCGTCAAATCCTGCTGCCTGTTGGCACTGTCTGCACCAGGGTTGACTCGGAAAACTAA

>India........Barramundi............2013....Fish(W).JF412270

ATGGTACACAAAGGTGAGAAGAAATTGGCAAAACCCGCGACCACCAAGGCCGCGAATCCGCAACCCCGCCGACGTGCTAACAATCGTCGGCGTAGTAATCGCACTGACGCACCTGTGTCAAAGGCCTCGACTGTGACTGGATTTGGACGTGGGACCAATGACGTCCATCTCTCAGGTATGTCGAGAATCTCCCAGGCCGTCCTCCCAGCCGGGACAGGAACAGACGGATACGTTGTTGTTGACGCAACCATCGTCCCCGACCTCCTGCCACGACTGGGACACGCTGCTAGAATCTTCCAGCGATACGCTGTTGAAACACTGGAGTTTGAAATTCAGCCAATGTGCCCCGCAAACACGGGCGGTGGTTACGTTGCTGGCTTCCTGCCTGATCCAACTGACAACGACCACACCTTCGATGCGCTTCAAGCAACTCGTGGTGCAGTCGTTGCCAAATGGTGGGAAAGCAGAACAGTCCGACCTCAGTACACCCGCACGCTCCTCTGGACCTCGTCGGGAAAGGAGCAGCGTCTCACGTCACCTGGTCGGCTGTTACTCCTGTGTGTCGGCAACAACACTGATGTGGTCAACGTGTCAGTACTGTGTCGCTGGAGTGTTCGACTGAGCGTTCCATCTCTTGAGACACCTGAGGAGACAACCGCTCCCATCATGACACAAGGTTCCCTGTACAACGATTCCCTTTCCACTAATGACTTCAAGTCCATCCTCCCAGGATCCACACCACTGGACATTGCCCCTGATGGAGCAGTCTTCCAGCTGGACCGTCCGCTGTCCATTGACTACAGCCTTGGAACTGGAGATGTTGATCGAGCTGTCTATTGGCACCTCAAGAAGTTTGCTGGAAATGCTGGCACACCTGCAGGCTGGTTTCGCTGGGGCATCTGGGACAACTTCAACAAGACGTTCACAGATGGCGTTGCCTACTACTCTGATGAGCAGCCCCGTCAAATCCTGCTGCCTGTTGGCACTGTCTGCACCAGGGTTGACTCGGAAAACTAA

>India........Barramundi............2013....Fish....JF412271

ATGGTACGCAAAGGTGAGAAGAAAAAGGCAAAACCCGCGACCACCAAGGCCGCGAATCCGCAACCCCGCCGACGTGCTAACAATCGTCGGCGTAGTAATCGCACTGACGCACCTGTGTCAAAGGCCTCGACTGTGACTGGATTTGGACGTGGGACCAATGACGTCCATCTCTCAGGTATGTCGAGAATCTCCCAGGCCGTCCTCCCAGCCGGGACAGGAACAGACGGATACGTTGTTGTTGACGCAACCATCGTCCCCGACCTCCTGCCACGACTGGGACACGCTGCTAGAATCTTCCAGCGATACGCTGTTGAAACACTGGAGTTTGAAATTCAGCCAATGTGCCCCGCAAACACGGGCGGTGGTTACGTTGCTGGCTTCCTGCCTGATCCAACTGACAACGACCACACCTTCGATGCGCTTCAAGCAACTCGTGGTGCAGTCGTTGCCAAATGGTGGGAAAGCAGAACAGTCCGACCTCAGTACACCCGCACGCTCCTCTGGACCTCGTCGGGAAAGGAGCAGCGTCTCACGTCACCTGGTCGGCTGATACTCCTGTGTGTCGGCAACAACACTGATGTGGTCAACGTGTCAGTACTGTGTCGCTGGAGTGTTCGACTGAGCGTTCCATCTCTTGAGACACCTGAGGAGACAACCGCTCCCATCATGACACAAGGTTCCCTGTACAACGATTCCCTTTCCACAAATGACTTCAAGTCCATCCTCCTAGGATCCACACCACTGGACATTGCCCCTGATGGAGCAGTCTTCCAGCTGGACCGTCCGCTGTCCATTGACTACAGCCTTGGAACTGGAGATGTTGATCGAGCTGTCTATTGGCACCTCAAGAAGTTTGCTGGAAATGCTGGCACACCTGCAGGCTGGTTTCGCTGGGGCATCTGGGACAACTTCAACAAGACGTTCACAGATGGCGTTGCCTACTACTCTGATGAGCAGCCCCGTCAAATCCTGCTGCCTGTTGGCACTGTCTGCACCAGGGTTGACTCGGAAAACTAA

>India........Barramundi............2013....Fish(W).JF412272

ATGGTACGCAAAGGTGAGAAGAAATTGGCAAAACCCGCGACCACCAAGGCCGCGAATCCGCAACCCCGCCGACGTGCTAACAATCGTCGGCGTAGTAATCGCACTGACGCACCCGTGTCAAAGGCCTCGACTGTGACTGGATTTGGACGTGGGACCAATGACGTCCATCTCTCAGGTATGTCGAGAATCTCCCAGGCCGTCCTCCCAGCCGGGACAGGAACAGACGGATACGTTGTTGTTGACGCAACCATCGTCCCCGACCTCCTGCCACGACTGGGACACGCTGCTAGAATCTTCCAGCGATACGCTGTTGAAACACTGGAGTTTGAAATTCAGCCAATGTGCCCCGCAAACACGGGCGGTGGTTACGTTGCTGGCTTCCTGCCTGATCCAACTGACAACGACCACACCTTCGATGCGCTTCAAGCAACTCGTGGTGCAGTCGTTGCCAAATGGTGGGAAAGCAGAACAGTCCGACCTCAGTACACCCGCACGCTCCTCTGGACCTCGTCGGGAAAGGAGCAGCGTCTCACGTCACCTGGTCGGCTGATACTCCTGTGTGTCGGCAACAACACTGATGTGGTCAACGTGTCAGTACTGTGTCGCTGGAGTGTTCGACTGAGCGTTCCATCTCTTGAGACACCTGAGGAGACAACCGCTCCCATCATGACACAAGGTTCCCTGTACAACGATTCCCTTTCCACAAATGACTTCAAGTCCATCCTCCTAGGATCCACACCACTGGACATTGCCCCTGATGGAGCAGTCTTCCAGCTGGACCGTCCGCTGTCCATTGACTACAGCCTTGGAACTGGAGATGTTGATCGAGCTGTCTATTGGCACCTCAAGAAGTTTGCTGGAAATGCTGGCACACCTGCAGGCTGGTTTCGCTGGGGCATCTGGGACAACTTCAACAAGACGTTCACAGATGGCGTTGCCTACTTCTCTGATGAGCAGCCCCGTCAAATCCTGCTGCCTGTTGGCACTGTCTGCACCAGGGTTGACTCGGAAAACTAA

>India........Barramundi............2013....Fish(W).JF412273

ATGGTACGCAAAGGTGAGAAGAAAATGACAAAACCCGCGACCACCAAGGCCGCGAATCCGCAATCCCGCCGACGTGCTAACAATCGTCGGCGTAGTAATCGCACTGACGCACCTGTGTCAAAGGCCTCGACTGTGACTGGATTTGGACGTGGGACCAATGACGTCCATCTCTCAGGTATGTCGAGAATCTCCCAGGCCGTCCTCCCAGCCGGGACAGGAACAGACGGATACGTTGTTGTTGACGCAACCATCGTCCCCGACCTCCTGCCACGACTGGGACACGCTGCTAGAATCTTCCAGCGATACGCTGTTGAAACACTGGAGTTTGAAATTCAGCCAATGTGCCCCGCAAACACGGGCGGTGGTTACGTTGCTGGCTTCCTGCCTGATCCAACTGACAACGACCACACCTTTGATGCGCTTCAAGCAACTCGTGGTGCAGTCGTTGCCAAATGGTGGGAAAGCAGAACAGTCCGACCTCAGTACACCCGCACGCTCCTCTGGACCTCGTCGGGAAAGGAGCAGCGTCTCACGTCACCTGGTCGGCTGATACTCCTGTGTGTCGGCAACAACACTGATGTGGTCAACGTGTCAGTACTGTGTCGCAGGAGTGTTCGACTGAGCGTTCCATCTCTTGAGACACCTGAGGAGACAACCGCTCCCATCATGACACAAGGTTCCCTGTACAACGATTCCCTTTCCACAAATGACTTCAAGTCCATCCTCCTAGGATCCACACCACTGGACATTGCCCCTGATGGAGCAGTCTTCCAGCTGGACCGTCCGCTGTCCATTGACTACAGCCTTGGAACTGGAGATGTTGATCGAGCTGTCTATTGGCACCTCAAGAAGTTTGCTGGAAATGCTGGCACACCTGCAGGCTGGTTTCGCTGGGGCATCTGGGACAACTTCAACAAGACGTTCACAGATGGCGTTGCCTACTACTCTGATGAGCAGCCCCGTCAAATCCTGCTGCCTGTTGGCACTGTCTGCACCAGGGTTGACTCGGAAAACTAA

>Australia(N).Barramundi............2007....Cell....GQ402011

ATGGTACGCAAAGGTGAGAAGAAATTGGCAAAACCCGCGACCACCAAGGCCGCGAATCCGCAACCCCGCCGACGTGCTAACAATCGTCGGCGTAGTAATCGCACTGACGCACCTGTGTCTAAGGCCTCGACTGTAACTGGATTTGGACGTGGGACCAATGACGTCCATCTCTCAGGTATGTCGAGAATCTCCCAGGCCGTCCTCCCAGCCGGGACAGGAACAGACGGATACGTTGTTGTTGACGCAACCATCGTCCCCGACCTCCTGCCACGACTGGGACACGCTGCTAGAATCTTCCAGCGATACGCTGTTGAAACACTGGAGTTTGAAATTCAGCCAATGTGCCCCGCAAACACGGGCGGTGGTTACGTTGCTGGCTTCCTGCCTGATCCAACTGACAACGACCACACCTTCGACGCGCTTCAAGCAACTCGTGGTGCAGTCGTTGCCAAATGGTGGGAAAGCAGAACAGTCCGACCTCAGTACACCCGTACGCTCCTCTGGACCTCGTCGGGAAAGGAGCAGCGTCTCACGTCACCTGGTCGGCTGATACTCCTGTGTGTCGGCAACAATACTGATGTTGTCAACGTGTCAGTGCTGTGTCGCTGGAGTGTTCGACTGAGCGTTCCATCTCTTGAGACACCTGAAGAGACCACCGCTCCCATCATGACACAAGGTCCCCTGTACAACGATTCCCTTTCCACAAATGACTTCAAGTCCATCCTCCTAGGATCCACGCCACTGGACATTGCCCCTGATGGAGCAGTCTTCCAGCTGGACCGTCCGCTGTCCATTGACTACAGCCTTGTAACTGGAGATGTTGACCGTGCTGTTTACTGGCACCTCAAGAAGTTTGCTGGAAATGCTGGCACACCTGCAGGCTGGTTTCGCTGGGGCATCTGGGACAACTTCAACAAGACGTTCACAGATGGCGTTGCCTACTACTCTGATGAGCAGCCCCGTCAAATCCTGCTGCCTGTTGGCACTGTCTGCACCAGGGTTGACTCGGGAAACTAA

>Australia(E).Bass..................2006....Cell....GQ402013

ATGGTACGCAAGGGTGAGAAGAAATTGGCAAAACCCGCGACCACCAAGGCCGCAAACCCACAACCGCGTCGACGTGCCAACAATCGCCGGCGTAGTAATCGCACTGATGCGCCTGTGTCTAAGGCCTCGACTGTGACTGGATTTGGACGCGGGACCAATGATGTCCATCTCTCAGGTATGTCGAGAATCTCCCAGGCCGTCCTCCCAGCCGGGACAGGAACTGACGGATACGTCGTTGTTGATGCAACTATCGTCCCCGACCTCCTGCCACGACTGGGACACGCTGCTAGAATCTTCCAGCGATACGCTGTTGAAACACTGGAGTTCGAAATTCAGCCAATGTGCCCCGCAAACACGGGCGGTGGTTACGTTGCTGGCTTCCTGCCTGATCCAACTGACAACGACCACACCTTCGACGCACTTCAAGCAACTCGTGGTGCGGTCGTGGCCAAGTGGTGGGAAAGCAGAACAGTCCGACCACAGTACACCCGCACGCTCCTCTGGACCTCGACGGGAAAGGAGCAGCGCCTCACATCACCTGGTCGGCTGATACTCCTGTGTGTCGGCAACAACACTGACGTGGTCAACGTGTCAGTTCTGTGTCGCTGGAGTGTTCGGCTGAGCGTTCCGTCTCTTGAAACACCTGAAGAGACGACCGCTCCCATCATGACACAAGGTCCCCTTTACAACGATTCCCTCGCCACCAGCGACTTCAAGTCCATCCTCCTGGGGTCCACGCAGTTGGACATTGCTCCTGATGGGGCAATCTTTCAATTGGACCGACCATTGTCCATTGATTACAGCCTAGGAACTGGTGATGTTGACCGTGCCGTCTACTGGCACCTTAAGAAGTTCGCTGGCACATCTGCCACACCTGCAGGCTGGTTTCGCTGGGGCATCTGGGACAACTTCAACAAAACGTTCACAGATGGCGTGGCTTACTACTCTGACGCGCAGCCTCGTCAAATTCTCCTGCCCGTTGGCACTGTCTGCACCAGGGTTGACTCGGAAAACTAA

>Indonesia....Grouper (Humpback)....2008(J).Fish....HQ859924

ATGGTACGCAAAGGTGAGAAGAAATTGGCAAAACCCGCGACCACCAAGGCCGCGAATCCGCAACCCCGCCGACGTGCTAACAATCGTCGGCGTAGTAACCGCACTGACGCACCTGTGTCTAAGGCCTCGACTGTGACTGGATTTGGACGTGGGACCAATGACGTCCATCTCTCAGGTATGTCGAGAATCTCCCAGGCCGTCCTCCCAGCCGGGACAGGAACAGACGGATACGTTGTTGTTGACGCAACCATCGTCCCCGACCTCCTGCCACGACTGGGACACGCTGCTAGAATCTTCCAGCGATACGCTGTTGAAACACTGGAGTTTGAAATTCAGCCAATGTGCCCCGCAAACACGGGCGGTGGTTACGTTGCTGGCTTCCTGCCTGATCCAACTGACAACGATCACACCTTCGACGCGCTTCAAGCAACTCGTGGTGCAGTCGTTGCCAAATGGTGGGAAAGCAGAACAGTCCGACCTCAGTACACCCGCACGCTCCTCTGGACCTCGTCGGGAAAGGAGCAGCGTCTCACGTCACCTGGTCGGCTGATACTCCTGTGTGTCGGCAACAACACTGACGTGGTCAACGTGTCGGTGCTGTGTCGCTGGAGTGTTCGACTGAGCGTTCCATCTCTTGAGACACCTGAAGAGACCACCGCTCCCATCATGACACAAGGTTCCCTGTACAACGATTCCCTTTCCACAAATGACTTCAAGTCCATCCTCCTAGGATCCACACCGCTGGACATTGCCCCTGATGGAGCAGTCTTCCAGCTGGACCGTCCGCTGTCCATTGACTACAGCCTTGGAACTGGAGATGTTGACCGTGCTGTTTACTGGCACCTCAAGAAGTTTGCTGGAAATGCTGGCACACCTGCAGGCTGGTTTCGCTGGGGCATCTGGGACAACTTCAACAAGACGTTCACAGATGGCGTTGCTTACTACTCTGATGAGCAGCCTCGTCAAATCCTGCTGCCTGTTGGCACTGTCTGCACCAGGGTTGACTCGGAAAACTAA

>Indonesia....Grouper (Humpback)....2008(J).Fish....HQ859925

ATGGTACGCAAAGGTGAGAAGAAATTGGCAAAACCCGCGACCACCAAGGCCGCGAATCCGCAACCCCGCCGACGTGCTAACAATCGTCGGCGTAGTAACCGCACTGACGCACCTGTGTCTAAGGCCTCGACTGTGACTGGATTTGGACGTGGGACCAATGACGTCCATCTCTCAGGTATGTCGAGAATCTCCCAGGCCGTCCTCCCAGCCGGGACAGGAACAGACGGATACGTTGTTGTTGACGCAACCATCGTCCCCGACCTCCTGCCACGACTGGGACACGCTGCTAGAATCTTCCAGCGATACGCTGTTGAAACACTGGAGTTTGAAATTCAGCCAATGTGCCCCGCAAACACGGGCGGTGGTTACGTTGCTGGCTTCCTGCCTGATCCAACTGACAACGATCACACCTTCGACGCGCTTCAAGCAACTCGTGGTGCAGTCGTTGCCAAATGGTGGGAAAGCAGAACAGTCCGACCTCAGTACACCCGCACGCTCCTCTGGACCTCGTCGGGAAAGGAGCAGCGTCTCACGTCACCTGGTCGGCTGATACTCCTGTGTGTCGGCAACAACACTGACGTGGTCAACGTGTCGGTGCTGTGTCGCTGGAGTGTTCGACTGAGCGTTCCATCTCTTGAGACACCTGAAGAGACCACCGCTCCCATCATGACACAAGGTTCCCTGTACAACGATTCCCTTTCCACAAATGACTTCAAGTCCATCCTCCTAGGATCCACACCGCTGGACATTGCCCCTGATGGAGCAGTCTTCCAGCTGGACCGTCCGCTGTCCATTGACTACAGCCTTGGAACTGGAGATGTTGACCGTGCTGTTTACTGGCACCTCAAGAAGTTTGCTGGAAATGCTGGCACACCTGCAGGCTGGTTTCGCTGGGGCATCTGGGACAACTTCAACAAGACGTTCACAGATGGCGTTGCTTACTACTCTGATGAGCAGCCTCGTCAAATCCTGCTGCCTGTTGGCACTGTCTGCACCAGGGTTGACTCGGAAAACTAA

>Indonesia....Grouper (Humpback)....2008(J).Fish....HQ859926

ATGGTACGCAAAGGTGAGAAGAAATTGGCAAAACCCGCGACCACCAAGGCCGCGAATCCGCAACCCCGCCGACGTGCTAACAATCGTCGGCGTAGTAACCGCACTGACGCACCTGTGTCTAAGGCCTCGACTGTGACTGGATTTGGACGTGGGACCAATGACGTCCATCTCTCAGGTATGTCGAGAATCTCCCAGGCCGTCCTCCCAGCCGGGACAGGAACAGACGGATACGTTGTTGTTGACGCAACCATCGTCCCCGACCTCCTGCCACGACTGGGACACGCTGCTAGAATCTTCCAGCGATACGCTGTTGAAACACTGGAGTTTGAAATTCAGCCAATGTGCCCCGCAAACACGGGCGGTGGTTACGTTGCTGGCTTCCTGCCTGATCCAACTGACAACGATCACACCTTCGACGCGCTTCAAGCAACTCGTGGTGCAGTCGTTGCCAAATGGTGGGAAAGCAGAACAGTCCGACCTCAGTACACCCGCACGCTCCTCTGGACCTCGTCGGGAAAGGAGCAGCGTCTCACGTCACCTGGTCGGCTGATACTCCTGTGTGTCGGCAACAACACTGACGTGGTCAACGTGTCGGTGCTGTGTCGCTGGAGTGTTCGACTGAGCGTTCCATCTCTTGAGACACCTGAAGAGACCACCGCTCCCATCATGACACAAGGTTCCCTGTACAACGATTCCCTTTCCACAAATGACTTCAAGTCCATCCTCCTAGGATCCACACCGCTGGACATTGCCCCTGATGGAGCAGTCTTCCAGCTGGACCGTCCGCTGTCCATTGACTACAGCCTTGGAACTGGAGATGTTGACCGTGCTGTTTACTGGCACCTCAAGAAGTTTGCTGGAAATGCTGGCACACCTGCAGGCTGGTTTCGCTGGGGCATCTGGGACAACTTCAACAAGACGTTCACAGATGGCGTTGCTTACTACTCTGATGAGCAGCCTCGTCAAATCCTGCTGCCTGTTGGCACTGTCTGCACCAGGGTTGACTCGGAAAACTAA

>Indonesia....Grouper (Tiger).......2008(J).Fish....HQ859927

ATGGTACGCAAAGGTGAGAAGAAATTGGCAAAACCCGCGACCACCAAGGCCGCGAATCCGCAACCCCGCCGACGTGCTAACAATCGTCGGCGTAGTAACCGCACTGACGCACCTGTGTCTAAGGCCTCGACTGTGACTGGATTTGGACGTGGGACCAATGACGTCCATCTCTCAGGTATGTCGAGAATCTCCCAGGCCGTCCTCCCAGCCGGGACAGGAACAGACGGATACGTTGTTGTTGACGCAACCATCGTCCCCGACCTCCTGCCACGACTGGGACACGCTGCTAGAATCTTCCAGCGATACGCTGTTGAAACACTGGAGTTTGAAATTCAGCCAATGTGCCCCGCAAACACGGGCGGTGGTTACGTTGCTGGCTTCCTGCCTGATCCAACTGACAACGATCACACCTTCGACGCGCTTCAAGCAACTCGTGGTGCAGTCGTTGCCAAATGGTGGGAAAGCAGAACAGTCCGACCTCAGTACACCCGCACGCTCCTCTGGACCTCGTCGGGAAAGGAGCAGCGTCTCACGTCACCTGGTCGGCTGATACTCCTGTGTGTCGGCAACAACACTGACGTGGTCAACGTGTCGGTGCTGTGTCGCTGGAGTGTTCGACTGAGCGTTCCATCTCTTGAGACACCTGAAGAGACCACCGCTCCCATCATGACACAAGGTTCCCTGTACAACGATTCCCTTTCCACAAATGACTTCAAGTCCATCCTCCTAGGATCCACACCACTGGATATTGCCCCTGATGGAGCAATCTTCCAGCTGGACCGTCCGCTGTCTATTGACTACAGCCTTGGAACTGGAGATGTTGACCGTGCTGTTTATTGGCACCTCAAGAAGTTTGCTGGAAATGCTGGCACACCTGCAGGCTGGTTTCGCTGGGGCATCTGGGACAACTTCAACAAGACGTTCACAGATGGCGTTGCTTACTATTCTGATGAGCAGCCCCGTCAAATTCTGCTGCCTGTTGGCACTGTCTGCACCAGGGTTGACTCGGAAAACTAA

>Indonesia....Grouper (Tiger).......2008(J).Fish....HQ859928

ATGGTACGCAAAGGTGAGAAGAAATTGGCAAAACCCGCGACCACCAAGGCCGCGAATCCGCAACCCCGCCGACGTGCTAACAATCGTCGGCGTAGTAACCGCACTGACGCACCTGTGTCTAAGGCCTCGACTGTGACTGGATTTGGACGTGGGACCAATGACGTCCATCTCTCAGGTATGTCGAGAATCTCCCAGGCCGTCCTCCCAGCCGGGACAGGAACAGACGGATACGTTGTTGTTGACGCAACCATCGTCCCCGACCTCCTGCCACGACTGGGACACGCTGCTAGAATCTTCCAGCGATACGCTGTTGAAACACTGGAGTTTGAAATTCAGCCAATGTGCCCCGCAAACACGGGCGGTGGTTACGTTGCTGGCTTCCTGCCTGATCCAACTGACAACGATCACACCTTCGACGCGCTTCAAGCAACTCGTGGTGCAGTCGTTGCCAAATGGTGGGAAAGCAGAACAGTCCGACCTCAGTACACCCGCACGCTCCTCTGGACCTCGTCGGGAAAGGAGCAGCGTCTCACGTCACCTGGTCGGCTGATACTCCTGTGTGTCGGCAACAACACTGACGTGGTCAACGTGTCGGTGCTGTGTCGCTGGAGTGTTCGACTGAGCGTTCCATCTCTTGAGACACCTGAAGAGACCACCGCTCCCATCATGACACAAGGTTCCCTGTACAACGATTCCCTTTCCACAAATGACTTCAAGTCCATCCTCCTAGGATCCACACCACTGGATATTGCCCCTGATGGAGCAATCTTCCAGCTGGACCGTCCGCTGTCTATTGACTACAGCCTTGGAACTGGAGATGTTGACCGTGCTGTTTATTGGCACCTCAAGAAGTTTGCTGGAAATGCTGGCACACCTGCAGGCTGGTTTCGCTGGGGCATCTGGGACAACTTCAACAAGACGTTCACAGATGGCGTTGCTTACTATTCTGATGAGCAGCCCCGTCAAATTCTGCTGCCTGTTGGCACTGTCTGCACCAGGGTTGACTCGGAAAACTAA

>Indonesia....Grouper (Tiger).......2008(J).Fish....HQ859929

ATGGTACGCAAAGGTGAGAAGAAATTGGCAAAACCCGCGACCACCAAGGCCGCGAATCCGCAACCCCGCCGACGTGCTAACAATCGTCGGCGTAGTAACCGCACTGACGCACCTGTGTCTAAGGCCTCGACTGTGACTGGATTTGGACGTGGGACCAATGACGTCCATCTCTCAGGTATGTCGAGAATCTCCCAGGCCGTCCTCCCAGCCGGGACAGGAACAGACGGATACGTTGTTGTTGACGCAACCATCGTCCCCGACCTCCTGCCACGACTGGGACACGCTGCTAGAATCTTCCAGCGATACGCTGTTGAAACACTGGAGTTTGAAATTCAGCCAATGTGCCCCGCAAACACGGGCGGTGGTTACGTTGCTGGCTTCCTGCCTGATCCAACTGACAACGATCACACCTTCGACGCGCTTCAAGCAACTCGTGGTGCAGTCGTTGCCAAATGGTGGGAAAGCAGAACAGTCCGACCTCAGTACACCCGCACGCTCCTCTGGACCTCGTCGGGAAAGGAGCAGCGTCTCACGTCACCTGGTCGGCTGATACTCCTGTGTGTCGGCAACAACACTGACGTGGTCAACGTGTCGGTGCTGTGTCGCTGGAGTGTTCGACTGAGCGTTCCATCTCTTGAGACACCTGAAGAGACCACCGCTCCCATCATGACACAAGGTTCCCTGTACAACGATTCCCTTTCCACAAATGACTTCAAGTCCATCCTCCTAGGATCCACACCACTGGATATTGCCCCTGATGGAGCAATCTTCCAGCTGGACCGTCCGCTGTCTATTGACTACAGCCTTGGAACTGGAGATGTTGACCGTGCTGTTTATTGGCACCTCAAGAAGTTTGCTGGAAATGCTGGCACACCTGCAGGCTGGTTTCGCTGGGGCATCTGGGACAACTTCAACAAGACGTTCACAGATGGCGTTGCTTACTATTCTGATGAGCAGCCCCGTCAAATTCTGCTGCCTGTTGGCACTGTCTGCACCAGGGTTGACTCGGAAAACTAA

>Malaysia 2...Pompano...............2006....Cell....GQ904199

ATGGTACGCAAAGGTGAGAAGAAATTGGCAAAACCCGCGACCACCAAGGCCGCAAATCCGCAACCCCGCCGACGTGCTAACAATCGTCGGCGTAGTAATCGCACTGACGCACCTGTGTCTAAGGCCTCGACTGTGACTGGATTTGGACGTGGGACCAATGACGTCCATCTCTCAGGTATGTCGAGAATCTCCCAGGCCGTCCTCCCAGCCGGGACAGGAACTGACGGATACGTTGTTGTTGACGCAACCATCGTCCCCGACCTCCTGCCACGACTGGGACACGCTGCTAGAATCTTCCAGCGATACGCTGTTGAAACACTGGAGTTTGAAATTCAGCCAATGTGCCCCGCAAACACGGGCGGTGGTTACGTTGCTGGCTTCCTGCCTGATCCAACTGACAACGACCACACCTTCGACGCGCTTCAAGCAACTCGTGGTGCAGTCGTTGCCAAATGGTGGGAAAGCAGAACAGTCCGACCCCAGTACACCCGTACGCTCCTCTGGACCTCGTCGGGAAAGGAGCAGCGTCTCACGTCACCTGGTCGGCTGATACTCCTGTGTGTCGGCAACAACACTGATGTGGTCAACGTGTCGGTGCTGTGTCGCTGGAGTGTTCGATTGAGCGTTCCATCTCTTGAGACACCTGAAGAGACCACCGCTCCCATCATGACACAAGGTTCCCTGTACAACGATTCCCTTTCCACAAATGACTTCAAATCCATCCTCCTAGGATCCACACCACTGGATATTGCCCCTGATGGAGCAGTCTTCCAGCTGGACCGTCCGCTGTCCATTGACTACAGCCTTGGAACTGGAGATGTTGACCGTGCTGTTTATTGGCACCTCAAGAAGTTTGCTGGAAATGCTGGCACACCTGCAGGCTGGTTTCGCTGGGGCATCTGGGACAACTTCAATAAGACGTTCACAGATGGCGTTGCCTACTACTCTGATGAGCAGCCCCGCCAAATCCTGCTGCCTGTTGGCACTGTCTGCACCAGGGTTGACTCGGAAAACTAA

>Malaysia 2...Pompano...............2008....Fish....HQ859932

ATGGTACGCAAAGGTGAGAAGAAATTGGCAAAACCCGCGACCACCAAGGCCGCGAATCCGCAACCCCGCCGACGTGCTAACAATCGTCGGCGTAGTAACCGCACTGACGCACCTGTGTCTAAGGCCTCGACTGTGACTGGATTTGGACGTGGGACCAATGACGTCCATCTCTCAGGTATGTCGAGAATCTCCCAGGCCGTCCTCCCAGCCGGGACAGGAACTGACGGATACGTTGTCGTTGACGCAACCATCGTCCCCGACCTCCTGCCACGACTGGGACACGCTGCTAGAATCTTCCAGCGATACGCTGTTGAAACACTGGAGTTTGAAATTCAGCCAATGTGCCCCGCAAACACGGGCGGTGGTTACGTTGCTGGCTTCCTGCCTGATCCAACTGACAACGACCACACCTTCGACGCGCTTCAAGCAACTCGTGGTGCAGTCGTTGCCAAATGGTGGGAAAGCAGAACAGTCCGACCTCAGTACACCCGCACGCTCCTCTGGACCTCGTCGGGAAAGGAGCAGCGTCTCACGTCACCTGGTCGGCTGATACTCCTGTGTGTCGGCAACAACACTGATGTGGTCAACGTGTCAGTGCTGTGTCGCTGGAGTGTTCGACTGAGCGTTCCATCTCTTGAGACACCTGAAGAGACGACCGCTCCCATCATGACACAAGGTTCCCTGTACAACGATTCCCTTTCCACAAATGACTTCAAGTCCATCCTCCTAGGATCCACACCACTGGATATTGCCCCTGATGGAGCAGTCTTCCAGCTGGACCGTCCGCTGTCCATTGACTACAGCCTTGGAACTGGAGATGTTGACCGTGCTGTTTATTGGCACCTCAAGAAGTTTGCTGGAAATGCTGGCACACCTGCAGGCTGGTTTCGCTGGGGCATCTGGGACAACTTCAACAAGACGTTCACAGATGGCGTTGCCTACTACTCTGATGAGCAGCCCCGTCAAATCCTGCTGCCTGTTGGCACTGTCTGCACCAGGGTTGACTCGGAAAACTAA

>Malaysia 2...Grouper (Tiger).......2009(J).Fish....HQ859940

ATGGTACGCAAAGGTGATAAGAAATTGGCAAAACCCGCGACCACCAAGGCCGCGAATCCGCAACCCCGCCGACGTGCTAACAATCGTCGGCGTAGTAACCGCACTGACGCACCTGTGTCAAAGGCCTCGACTGTGACTGGATTTGGACGTGGGACCAATGACGTCCATCTCTCAGGTATGTCGAGAATCTCCCAGGCCGTCCTCCCAGCCGGGACAGGAACAGACGGATACGTTGTTGTTGACGCAACCATCGTCCCCGACCTCCTGCCACGACTGGGACACGCTGCTAGAATCTTCCAGCGATACGCTGTTGAAACACTGGAGTTTGAAATTCAGCCAATGTGCCCCGCAAACACGGGCGGTGGTTACGTTGCTGGCTTCCTGCCTGATCCAACTGACAACGACCACACCTTCGACGCGCTTCAAGCAACTCGTGGTGCAGTCGTTGCCAAATGGTGGGAAAGCAGAACAGTCCGACCTCAGTACACCCGCACGCTCCTCTGGACCTCGTCGGGAAAGGAGCAGCGTCTCACGTCACCTGGTCGGCTGATACTCCTGTGTGTCGGCAACAACACTGATGTGGTCAACGTGTCAGTACTGTGTCGCTGGAGTGTTCGACTGAGCGTTCCATCTCTTGAGACACCTGAGGAGACAACCGCTCCCATCATGACACAAGGTTCCCTGTACAACGATTCCCTTTCCACAAATGACTTCAAGTCCATCCTCCTAGGATCCACACCACTGGACATTGCCCCTGATGGAGCAGTCTTCCAGCTGGACCGTCCGCTGTCCATTGACTACAGCCTTGGAACTGGAGATGTTGATCGAGCTGTCTATTGGCACCTCAAGAAGTTTGCTGGAAATGCTGGCACACCTGCAGGCTGGTTTCGCTGGGGCATCTGGGACAACTTCAACAAGACGTTCACAGATGGCGTTGCCTACTACTCTGATGAGCAGCCCCGTCAAATCCTGCTGCCTGTTGGCACTGTCTGCACCAGGGTTGACTCGGAAAACTAA

>Malaysia 2...Grouper (Tiger).......2009(J).Fish....HQ859941

ATGGTACGCAAAGGTGATAAGAAATTGGCAAAACCCGCGACCACCAAGGCCGCGAATCCGCAACCCCGCCGACGTGCTAACAATCGTCGGCGTAGTAACCGCACTGACGCACCTGTGTCAAAGGCCTCGACTGTGACTGGATTTGGACGTGGGACCAATGACGTCCATCTCTCAGGTATGTCGAGAATCTCCCAGGCCGTCCTCCCAGCCGGGACAGGAACAGACGGATACGTTGTTGTTGACGCAACCATCGTCCCCGACCTCCTGCCACGACTGGGACACGCTGCTAGAATCTTCCAGCGATACGCTGTTGAAACACTGGAGTTTGAAATTCAGCCAATGTGCCCCGCAAACACGGGCGGTGGTTACGTTGCTGGCTTCCTGCCTGATCCAACTGACAACGACCACACCTTCGACGCGCTTCAAGCAACTCGTGGTGCAGTCGTTGCCAAATGGTGGGAAAGCAGAACAGTCCGACCTCAGTACACCCGCACGCTCCTCTGGACCTCGTCGGGAAAGGAGCAGCGTCTCACGTCACCTGGTCGGCTGATACTCCTGTGTGTCGGCAACAACACTGATGTGGTCAACGTGTCAGTACTGTGTCGCTGGAGTGTTCGACTGAGCGTTCCATCTCTTGAGACACCTGAGGAGACAACCGCTCCCATCATGACACAAGGTTCCCTGTACAACGATTCCCTTTCCACAAATGACTTCAAGTCCATCCTCCTAGGATCCACACCACTGGACATTGCCCCTGATGGAGCAGTCTTCCAGCTGGACCGTCCGCTGTCCATTGACTACAGCCTTGGAACTGGAGATGTTGATCGAGCTGTCTATTGGCACCTCAAGAAGTTTGCTGGAAATGCTGGCACACCTGCAGGCTGGTTTCGCTGGGGCATCTGGGACAACTTCAACAAGACGTTCACAGATGGCGTTGCCTACTACTCTGATGAGCAGCCCCGTCAAATCCTGCTGCCTGTTGGCACTGTCTGCACCAGGGTTGACTCGGAAAACTAA

>Malaysia 2...Grouper (Tiger).......2009(J).Fish....HQ859942

ATGGTACGCAAAGGTGATAAGAAATTGGCAAAACCCGCGACCACCAAGGCCGCGAATCCGCAACCCCGCCGACGTGCTAACAATCGTCGGCGTAGTAACCGCACTGACGCACCTGTGTCAAAGGCCTCGACTGTGACTGGATTTGGACGTGGGACCAATGACGTCCATCTCTCAGGTATGTCGAGAATCTCCCAGGCCGTCCTCCCAGCCGGGACAGGAACAGACGGATACGTTGTTGTTGACGCAACCATCGTCCCCGACCTCCTGCCACGACTGGGACACGCTGCTAGAATCTTCCAGCGATACGCTGTTGAAACACTGGAGTTTGAAATTCAGCCAATGTGCCCCGCAAACACGGGCGGTGGTTACGTTGCTGGCTTCCTGCCTGATCCAACTGACAACGACCACACCTTCGACGCGCTTCAAGCAACTCGTGGTGCAGTCGTTGCCAAATGGTGGGAAAGCAGAACAGTCCGACCTCAGTACACCCGCACGCTCCTCTGGACCTCGTCGGGAAAGGAGCAGCGTCTCACGTCACCTGGTCGGCTGATACTCCTGTGTGTCGGCAACAACACTGATGTGGTCAACGTGTCAGTACTGTGTCGCTGGAGTGTTCGACTGAGCGTTCCATCTCTTGAGACACCTGAGGAGACAACCGCTCCCATCATGACACAAGGTTCCCTGTACAACGATTCCCTTTCCACAAATGACTTCAAGTCCATCCTCCTAGGATCCACACCACTGGACATTGCCCCTGATGGAGCAGTCTTCCAGCTGGACCGTCCGCTGTCCATTGACTACAGCCTTGGAACTGGAGATGTTGATCGAGCTGTCTATTGGCACCTCAAGAAGTTTGCTGGAAATGCTGGCACACCTGCAGGCTGGTTTCGCTGGGGCATCTGGGACAACTTCAACAAGACGTTCACAGATGGCGTTGCCTACTACTCTGATGAGCAGCCCCGTCAAATCCTGCTGCCTGTTGGCACTGTCTGCACCAGGGTTGACTCGGAAAACTAA

>Malaysia 1d..Barramundi............2008....Fish....GQ120525

ATGGTACGCAAGGGTGAGAAGAAATTGGCAAAACCCGCGACCACAAAGGCCGCGAATCCGCAACCCCGTCGACGTGCTAACAATCGTCGGCGTAGTAACCGCACTGACGCACCTGTGTCAAAGGCCTCGACAGTAACTGGATTTGGACGTGGGACCAATGACGTCCATCTCTCAGGTATGTCGAGAATCTCCCAGGCCGTCCTCCCAGCCGGGACAGGAACAGACGGATACGTTGTTGTTGACGCAACCATCGTCCCCGACCTCCTGCCACGACTGGGACACGCTGCTAGAATCTTCCAGCGATACGCTGTTGAAACACTGGAGTTTGAAATTCAGCCAATGTGCCCCGCAAACACGGGCGGTGGTTACGTTGCTGGCTTCCTGCCTGATCCAACTGACAACGATCACACCTTCGACGCGTTTCAAGCAACTCGTGGTGCAGTCGTTGCCAAATGGTGGGAAAGCAGAACAGTCCGACCACAGTACACCCGCACGCTCCTCTGGACCTCGTCGGGAAAGGAGCAGCGTCTCACGTCACCTGGTCGGCTGATACTCCTGTGTGTCGGCAACAACACTGGCGTGGTCAACGTGTCGGTGCTGTGTCGCTGGAGTGTTCGACTGAGCGTCCCGTCTCTTGAGACACCTGAAGAGACTACCGCTCCTATCATGACACAAGGTTCCCTGTACAACGATTCCCTTGCTACAAATGACTTCAAGTCCATCCTCCTAGGATCCACACCACTGGACATTGCCCCTGATGGAGCAGTCTTCCAGCTGGACCGTCCGCTGTCTATTGACTACAGCCTTGGAACTGGGGATGTTGACCGTGCTGTTTACTGGCACCTCAAGAAGTTTGCTGGAAATGTTACCACACCTGCAGGCTGGTTTCGCTGGGGCATCTGGGACAACTTCAACAAAACGTTCACAGATGGCGTTGCTTACTACTCTGATGAGCAGCCTCGTCAAATTCTGCTGCCTGTTGGCACTGTCTGCACCAGGGTTGACTCGGAAAACTAA

>Malaysia 1a..Barramundi............2007(F).Fish....HQ859919

ATGGTACGCAAAGGTGAGAAGAAATTGGCAAAACCCGCGACCACCAAGGCCGCGAATCCGCAACCCCGCCGACGTGCTAACAATCGTCGGCGTAGTAACCGCACTGACGCACCTGTGTCTAAGGCCTCGACTGTGACTGGATTTGGACGTGGGACCAATGACGTCCATCTCTCAGGTATGTCGAGAATCTCCCAGGCCGTCCTCCCAGCCGGGACAGGAACAGACGGATACGTTGTTGTTGACGCAACCATCGTCCCCGACCTCCTGCCACGACTGGGACACGCTGCTAGAATCTTCCAGCGATACGCTGTTGAAACACTGGAGTTTGAAATTCAGCCAATGTGCCCCGCAAACACGGGCGGTGGTTACGTTGCTGGCTTCCTGCCTGATCCAACTGACAACGATCACACCTTCGACGCGCTTCAAGCAACTCGTGGTGCAGTCGTTGCCAAAAGGTGGGAAAGCAGAACAGTCCGACCTCAGTACACCCGCACGCTCCTCTGGACCTCGTCGGGAAAGGAGCAGCGTCTCACGTCACCTGGTCGGCTGATACTCCTGTGTGTCGGCAACAACACTGACGTGGTCAACGTGTCGGTGCTGTGTCGCTGGAGTGTTCGACTGAGCGTTCCATCTCTTGAGACACCTGAGGAGACCACCGCTCCCATCATGACACAAGGTTCCCTGTACAACGATTCCCTTTCCACAAATGACTTCAAGTCCATCCTCCTAGGATCCACACCACTGGACATTGCCCCTGATGGAGCAGTCTTCCAGCTGGACCGTCCGCTGTCCATTGACTACAGCCTTGGAGCTGGAGATGTTGACCGTGCTGTTTACTGGCACCTCAAGAAGTTTGCTGGAAATGCTGGCACACCTGCAGGCTGGTTTCGCTGGGGCATCTGGGACAACTTCAACAAGACGTTCACAGATGGCGTTGCTTACTACTCTGATGAGCAGCCTCGTCAAATCCTGCTGCCTGTTGGCACTGTCTGCACCAGGGTTGACTCGGAAAACTAA

>Malaysia 1a..Barramundi............2007(J).Fish....HQ859922

ATGGTACGCAAAGGTGAGAAGAAATTGGCAAAACCCGCGACCACCAAGGCCGCGAATCCGCAACCCCGCCGACGTGCTAACAATCGTCGGCGTAGTAACCGCACTGACGCACCTGTGTCTAAGGCCTCGACTGTGACTGGATTTGGACGTGGGACCAATGACGTCCATCTCTCAGGTATGTCGAGAATCTCCCAGGCCGTCCTCCCAGCCGAGACAGGAACAGACGGATACGTTGTTGTTGACGCAACCATCGTCCCCGACCTCCTGCCACGACTGGGACACGCTGCTAGAATCTTCCAGCGATACGCTGTTGAAGCACTGGAGTTTGAAATTCAGCCAATGTGCCCCGCAAACACGGGCGGTGGTTACGTTGCTGGCTTCCTGCCTGATCCAACTGACAACGATCACACCTTCGACGCGCTTCAAGCAACTCGTGGTGCAGTCGTTGCCAAATGGTGGGAAAGCAGAACAGTCCGACCTCAGTACACCCGCACGCTCCTCTGGACCTCGTCGGGAAAGGAGCAGCGTCTCACGTCACCTGGTCGGCTGATACTCCTGTGTGTCGGCAACAACACTGACGTGGTCAACGTGTCGGTGCTGTGTCGCTGGAGTGTTCGACTGAGCGTTCCATCTCTTGAGACACCTGAGGAGACCACCGCTCCCATCATGACACAAGGTTCCCTGTACAACGATTCCCTTTCCACAAATGACTTCAAGTCCATCCTCCTAGGATCCACACCACTGGACATTGCCCCTGATGGAGCAGTCTTCCAGCTGGACCGTCCGCTGTCCATTGACTACAGCCTTGGAACTGGAGATGTTGACCGTGCTGTTTACTGGCACCTCAAGAAGTTTGCTGGAAATGCTGGCACACCTGCAGGCTGGTTTCGCTGGGGCATCTGGGACAACTTCAACAAGACGTTCACAGATGGCGTTGCTTACTACTGTGATGAGCAGCCTCGTCAAATCCTGGGACCTGTTGGCACGGTATGCACCAGGGTTGACTCGGAAAACTAA

>Malaysia 1a..Barramundi............2008(A).Fish....HQ859930

ATGGTACGCAAAGGTGAGAAGAAATTGGCAAAACCCGCGACCACCAAGGCCGCGAATCCGCAACCCCGTCGACGTGCTAACAATCGTCGGCGTAGTAACCGCACTGACGCACCTGTGTCAAAGGCCTCGACAGTAACTGGATTTGGACGTGGGACCAATGACGTCCATCTCTCAGGTATGTCGAGAATCTCCCAGGCCGTCCTCCCAGCCGGGACAGGAACAGACGGATACGTTGTTGTTGACGCAACCATCGTCCCCGACCTCCTGCCACGACTGGGACACGCTGCTAGAATCTTCCAGCGATACGCTGTTGAAACACTGGAGTTTGAAATTCAGCCAATGTGCCCCGCAAACACGGGCGGTGGTTACGTTGCTGGCTTCCTGCCTGATCCAACTGACAACGATCACACCTTCGACGCGCTTCAAGCAACTCGTGGTGCAGTCGTTGCCAAATGGTGGGAAAGCAGAACCAGTCGACCACAGTACACCCGCACGCTCCTCTGGACCTCGTCGGGAAAGGAGCAGCGTCTCACGTCACCTGGTCGGCTGATACTCCTGTGTGTCGGCAACAACACTGACGTGGTCAACGTGTCGGTGCTGTGTCGCTGGAGTGTTCGACTGAGCGTCCCGTCTCTTGAGACACCTGAAGAGACTACCGCTCCTATCATGACACAAGGTTCCCTGTACAACGATTCCCTTGCTACAAATGACTTCAAGTCCATCCTCCTAGGATCCACACCACTGGACATTGCCCCTGATGGAGCAGTCTTCCAGCTGGACCGTCCGCTGTCTATTGACTACAGCCTTGGAACTGGAGATGTTGACCGTGCTGTCTACTGGCACCTCAAGAAGTTTGCTGGAAATGTTACCACACCTGCAGGCTGGTTTCGCTGGGGCATCTGGGACAACTTCAACAAAACGTTCACAGATGGCGTTGCTTACTACTCTGATGAGCAGCCTCGTCAAATTCTGCTGCCTGTTGGCACTGTCTGCACCAGGGTTGACTCGGAAAACTAA

>Malaysia 1a..Barramundi............2008(N).Fish....HQ859935

ATGGTACGCAAAGGTGAGAAGAAATTGGCAAAACCCGCGCCCACCAAGGCCGCGAATCCGCAACCCCGTCGACGTGCTAACAATCGTCGGCGTAGTAACCGCACTGACGCACCTGCGTCAAAGGCCTCGACAGTAACTGGATTTGGACGTGGGACCAATGACGTCCATCTCTCAGGTATGTCGAGAATCTCCCAGGCCGTCCTCCCAGCCGGGACAGGAACAGACGGATACGTTGTTGTTGACGCAACCATCGTCCCCGACCTCCTGCCACGACTGGGACACGCTGCTAGAATCTTCCAGCGATACGCTGTTGAAACACTGGAGTTTGAAATTCAGCCAATGTGCCCCGCAAACACGGGCGGTGGTTACGTTGCTGGCTTCCTGCCTGATCCAACTGACAACGATCACACCTTCGACGCGCTTCAAGCAACTCGTGGTGCAGTCGTTGCCAAATGGTGGGAAAGCAGAACAGTCCGACCACAGTACACCCGCACGCTCCTCTGGACCTCGTCGGGAAAGGAGCAGCGTCTCACGTCACCTGGTCGGCTGATACTCCTGTGTGTCGGCAACAACACTGACGTGGTCAACGTGTCGGTGCTGTGTCGCTGGAGTGTTCGACTGAGCGTCCCGTCTCTTGAGACACCTGAAGAGACTACCGCTCCTATCATGACACAAGGTTCCCTGTACAACGATTCCCTTGCTACAAATGACTTCAAGTCCATCCTCCTAGGATCCACACCACTGGACATTGCCCCTGATGGAGCAGTCTTCCAGCTGGACCGTCCGCTGTCTATTGACTACAGCCTTGGAACTGGAGATGTTGACCGTGCTGTCTACTGGCACCTCAAGAAGTTTGCTGGAAATGTTACCACACCTGCAGGCTGGTTTCGCTGGGGCATCTGGGACAACTTCAACAAAACGTTCACAGATGGCGTTGCTTACTACTCTGATGAGCAGCCTCGTCAAATTCTGCTGCCTGTTGGCACTGTCTGCACCAGGGTTGACTCGGAAAACTAA

>Malaysia 1b..Grouper (Tiger).......2008(D).Fish....HQ859938

ATGGTACGCAAAGGTGAGAAGAAATTGGCAAAACCCGCGACCACCAAGGCCGCGAATCCGCAACCCCGTCGACGTGCTAACAATCGTCGGCGTAGTAACCGCACTGACGCACCTGTGTCAAAGGCCTCGACAGTAACTGGATTTGGACGTGGGACCAATGACGTCCATCTCTCAGGTATGTCGAGAATCTCCCAGGCCGTCCTCCCAGCCGGGACAGGAACAGACGGATACGTTGTTGTTGACGCAACCATCGTCCCCGACCTCCTGCCACGACTGGGACACGCTGCTAGAATCTTCCAGCGATACGCTGTTGAAACACTGGAGTTTGAAATTCAGCCAATGTGCCCCGCAAACACGGGCGGTGGTTACGTTGCTGGCTTCCTGCCTGATCCAACTGACAACGATCACACCTTCGACGCGCTTCAAGCAACTCGTGGTGCAGTCGTTGCCAAATGGTGGGAAAGCAGAACAGTCCGACCACAGTACACCCGCACGCTCCTCTGGACCTCGTCGGGAAAGGAGCAGCGTCTCACGTCACCTGGTCGGCTGATACTCCTGTGTGTCGGCAACAACACTGACGTGGTCAACGTGTCGGTGCTGTGTCGCTGGAGTGTTCGACTGAGCGTCCCGTCTCTTGAGACACCTGAAGAGACTACCGCTCCTATCATGACACAAGGTTCCCTGTACAACGATTCCCTTGCTACAAATGACTTCAAGTCCATCCTCCTAGGATCCACACCACTGGACATTGCCCCTGATGGAGCAGTCTTCCAGCTGGACCGTCCGCTGTCTATTGACTACAGCCTTGGAACTGGAGATGTTGACCGTGCTGTCTACTGGCACCTCAAGAAGTTTGCTGGAAATGTTACCACACCTGCAGGCTGGTTTCGCTGGGGCATCTGGGACAACTTCAACAAAACGTTCACAGATGGCGTTGCTTACTACTCTGATGAGCAGCCTCGTCAAATTCTGCTGCCTGTTGGCACTGTCTGCACCAGGGTTGACTCGGAAAACTAA

>Malaysia 1b..Grouper (Tiger).......2008(D).Fish....HQ859939

ATGGTACGCAAAGGTGAGAAGAAATTGGCAAAACCCGCGACCACCAAGGCCGCGAATCCGCAACCCCGTCGACGTGCTAACAATCGTCGGCGTAGTAACCGCACTGACGCACCTGTGTCAAAGGCCTCGACAGTAACTGGATTTGGACGTGGGACCAATGACGTCCATCTCTCAGGTATGTCGAGAATCTCCCAGGCCGTCCTCCCAGCCGGGACAGGAACAGACGGATACGTTGTTGTTGACGCAACCATCGTCCCCGACCTCCTGCCACGACTGGGACACGCTGCTAGAATCTTCCAGCGATACGCTGTTGAAACACTGGAGTTTGAAATTCAGCCAATGTGCCCCGCAAACACGGGCGGTGGTTACGTTGCTGGCTTCCTGCCTGATCCAACTGACAACGATCACACCTTCGACGCGCTTCAAGCAACTCGTGGTGCAGTCGTTGCCAAATGGTGGGAAAGCAGAACAGTCCGACCACAGTACACCCGCACGCTCCTCTGGACCTCGTCGGGAAAGGAGCAGCGTCTCACGTCACCTGGTCGGCTGATACTCCTGTGTGTCGGCAACAACACTGACGTGGTCAACGTGTCGGTGCTGTGTCGCTGGAGTGTTCGACTGAGCGTCCCGTCTCTTGAGACACCTGAAGAGACTACCGCTCCTATCATGACACAAGGTTCCCTGTACAACGATTCCCTTGCTACAAATGACTTCAAGTCCATCCTCCTAGGATCCACACCACTGGACATTGCCCCTGATGGAGCAGTCTTCCAGCTGGACCGTCCGCTGTCTATTGACTACAGCCTTGGAACTGGAGATGTTGACCGTGCTGTCTACTGGCACCTCAAGAAGTTTGCTGGAAATGTTACCACACCTGCAGGCTGGTTTCGCTGGGGCATCTGGGACAACTTCAACAAAACGTTCACAGATGGCGTTGCTTACTACTCTGATGAGCAGCCTCGTCAAATTCTGCTGCCTGTTGGCACTGTCTGCACCAGGGTTGACTCGGAAAACTAA

>Malaysia 1a..Barramundi............2009(J).Fish....HQ859943

ATGGTACGCAAAGGTGAGAAGAAATTGGCAAAACCCGCGACCACCAAGGCCGCAAATCCGCAACCCCGCCGACGTGCTAACAATCGTCGGCGTAGTAACCGCACTGACGCACCTGTGTCTAAGGCCTCGACTGTGACTGGATTTGGACGTGGGACCAATGACGTCCATCTCTCAGGCATGTCGAGAATCTCCCAGGCCGTCCTCCCAGCCGGGACAGGAACAGACGGATACGTTGTTGTTGACGCAACCATCGTCCCCGACCTCCTGCCACGACTGGGACACGCTGCTAGAATCTTCCAGCGATACGCTGTTGAAACACTGGAGTTTGAAATTCAGCCAATGTGCCCCGCAAACACGGGCGGTGGTTACGTTGCTGGCTTCCTGCCTGATCCAACTGACAACGATCACACCTTCGACGCGCTTCAAGCAACTCGTGGTGCAGTCGTTGCCAAATGGTGGGAAAGCAGAACAGTCCGACCCCAGTACACCCGCACGCTCCTCTGGACCTCGTCGGGAAAGGAGCAGCGTCTCACGTCACCTGGTCGGCTGATACTCCTGTGTGTCGGCAACAACACTGACGTGGTCAACGTGTCGGTGCTGTGTCGCTGGAGTGTTCGACTGAGCGTTCCATCTCTTGAGACACCTGAGGAGACCACCGCGCCCATCACGACACAAGGTTCCCTGTACAACGATTCCCTTTCCACAAATGACTTCAAGTCCATCCTCCTAGGATCCACACCACTGGACATTGCCCCTGATGGGGCAGTCTTCCAGCTGGACCGTCCGCTGTCCATTGACTACAGCCTTGGAACTGGAGATGTTGACCGTGCTGTTTATTGGCACCTCAAGAAGTTTGCTGGAAATGCTGGCACACCTGCAGGCTGGTTTCGCTGGGGCATCTGGGACAACTTCAACAAGACGTTCACAGATGGCGTTGCTTACTACTCTGATGAGCAGCCTCGTCAAATCCTGCTGCCTGTTGGCACCGTCTGCACCAGGGTTGACTCGGAAAACTAA

>Malaysia 1b..Grouper (Tiger).......2009(A).Fish....HQ859945

ATGGTACGCAAAGGTGAGAAGAAATTGGCAAAACCCGCGACCACCAAGGCCGCGAATCCGCAACCCCGCCGACGTGCTAACAATCGTCGGCGTAGTAACCGCACTGACGCACCTGTGTCTAAGGCCTCGACTGTCACTGGATTTGGACGTGGGACCAATGACGTCCATCTCTCAGGTATGTCGAGAATCTCCCAGGCCGTCCTCCCAGCCGGGACAGGAACAGACGGATACGTTGTTGTTGACGCAACCATCGTCCCCGACCTCCTGCCACGACTGGGACACGCTGCTAGAATCTTCCAGCGATACGCTGTTGAAACACTGGAGTTTGAAATTCAGCCAATGTGCCCCGCAAACACGGGCGGTGGTTACGTTGCTGGCTTCCTGCCTGATCCAACTGACAACGATCACACCTTCGACGCGCTTCAAGCAACTCGTGGTGCAGTCGTTGCCAAATGGTGGGAAAGCAGAACAGTCCGACCTCAGTACACCCGCACGCTCCTCTGGACCTCGTCGGGAAAGGAGCAGCGTCTCACGTCACCTGGTCGGCTGATACTCCTGTGTGTCGGCAACAACACTGACGTGGTCAACGTGTCGGTGCTGTGTCGCTGGAGTGTTCGACTGAGCGTTCCATCTCTTGAGACACCTGAGGAGACCACCGCTCCCATCATGACACAAGGTTCCCTGTACAACGATTCCCTTTCCACAAATGACTTCAAGTCCATCCTCCTAGGATCCACACCACTGGACATTGCCCCTGATGGAGCAGTCTTCCAGCTGGACCGTCCGCTGTCCATTGACTACAGCCTTGGAACTGGAGATGTTGACCGTGCTGTTTACTGGCACCTCAAGAAGTTTGCTGGAAATGCTGGCACACCTGCAGGCTGGTTTCGCTGGGGCATCTGGGACAACTTCAACAAGACGTTCACAGATGGCGTTGCTTACTACTCTGATGAGCAGCCTCGTCAAATCCTGCTGCCTGTTGGCACTGTCTGCACCAGGGTTGACTCGGAAAACTAA

>Malaysia 1b..Grouper (Tiger).......2009(A).Fish....HQ859946

ATGGTACGCAAAGGTGAGAAGAAATTGGCAAAACCCGCGACCACCAAGGCCGCGAATCCGCAACCCCGCCGACGTGCTAACAATCGTCGGCGTAGTAACCGCACTGACGCACCTGTGTCTAAGGCCTCGACTGTCACTGGATTTGGACGTGGGACCAATGACGTCCATCTCTCAGGTATGTCGAGAATCTCCCAGGCCGTCCTCCCAGCCGGGACAGGAACAGACGGATACGTTGTTGTTGACGCAACCATCGTCCCCGACCTCCTGCCACGACTGGGACACGCTGCTAGAATCTTCCAGCGATACGCTGTTGAAACACTGGAGTTTGAAATTCAGCCAATGTGCCCCGCAAACACGGGCGGTGGTTACGTTGCTGGCTTCCTGCCTGATCCAACTGACAACGATCACACCTTCGACGCGCTTCAAGCAACTCGTGGTGCAGTCGTTGCCAAATGGTGGGAAAGCAGAACAGTCCGACCTCAGTACACCCGCACGCTCCTCTGGACCTCGTCGGGAAAGGAGCAGCGTCTCACGTCACCTGGTCGGCTGATACTCCTGTGTGTCGGCAACAACACTGACGTGGTCAACGTGTCGGTGCTGTGTCGCTGGAGTGTTCGACTGAGCGTTCCATCTCTTGAGACACCTGAGGAGACCACCGCTCCCATCATGACACAAGGTTCCCTGTACAACGATTCCCTTTCCACAAATGACTTCAAGTCCATCCTCCTAGGATCCACACCACTGGACATTGCCCCTGATGGAGCAGTCTTCCAGCTGGACCGTCCGCTGTCCATTGACTACAGCCTTGGAACTGGAGATGTTGACCGTGCTGTTTACTGGCACCTCAAGAAGTTTGCTGGAAATGCTGGCACACCTGCAGGCTGGTTTCGCTGGGGCATCTGGGACAACTTCAACAAGACGTTCACAGATGGCGTTGCTTACTACTCTGATGAGCAGCCTCGTCAAATCCTGCTGCCTGTTGGCACTGTCTGCACCAGGGTTGACTCGGAAAACTAA

>Malaysia 1b..Grouper (Tiger).......2009(A).Fish....HQ859947

ATGGTACGCAAAGGTGAGAAGAAATTGGCAAAACCCGCGACCACCAAGGCCGCGAATCCGCAACCCCGCCGACGTGCTAACAATCGTCGGCGTAGTAACCGCACTGACGCACCTGTGTCTAAGGCCTCGACTGTCACTGGATTTGGACGTGGGACCAATGACGTCCATCTCTCAGGTATGTCGAGAATCTCCCAGGCCGTCCTCCCAGCCGGGACAGGAACAGACGGATACGTTGTTGTTGACGCAACCATCGTCCCCGACCTCCTGCCACGACTGGGACACGCTGCTAGAATCTTCCAGCGATACGCTGTTGAAACACTGGAGTTTGAAATTCAGCCAATGTGCCCCGCAAACACGGGCGGTGGTTACGTTGCTGGCTTCCTGCCTGATCCAACTGACAACGATCACACCTTCGACGCGCTTCAAGCAACTCGTGGTGCAGTCGTTGCCAAATGGTGGGAAAGCAGAACAGTCCGACCTCAGTACACCCGCACGCTCCTCTGGACCTCGTCGGGAAAGGAGCAGCGTCTCACGTCACCTGGTCGGCTGATACTCCTGTGTGTCGGCAACAACACTGACGTGGTCAACGTGTCGGTGCTGTGTCGCTGGAGTGTTCGACTGAGCGTTCCATCTCTTGAGACACCTGAGGAGACCACCGCTCCCATCATGACACAAGGTTCCCTGTACAACGATTCCCTTTCCACAAATGACTTCAAGTCCATCCTCCTAGGATCCACACCACTGGACATTGCCCCTGATGGAGCAGTCTTCCAGCTGGACCGTCCGCTGTCCATTGACTACAGCCTTGGAACTGGAGATGTTGACCGTGCTGTTTACTGGCACCTCAAGAAGTTTGCTGGAAATGCTGGCACACCTGCAGGCTGGTTTCGCTGGGGCATCTGGGACAACTTCAACAAGACGTTCACAGATGGCGTTGCTTACTACTCTGATGAGCAGCCTCGTCAAATCCTGCTGCCTGTTGGCACTGTCTGCACCAGGGTTGACTCGGAAAACTAA

>Malaysia 1c..Barramundi............2009(S).Fish....HQ859948

ATGGTACGCAAAGGTGAGAAGAAATTGGCAAAACCCGCGACCACCAAGGCCGCAAATCCGCAACCCCGCCGACGTGCTAACAATCGTCGGCGTAGTAACCGCACTGACGCACCTGTGTCTAAGGCCTCGACTGTGACTGGATTTGGACGTGGGACCAATGACGTCCATCTCTCAGGTATGTCGAGAATCTCCCAGGCCGTCCTCCCAGCCGGGACAGGAACAGACGGATACGTTGTTGTTGACGCAACCATCGTCCCCGACCTCCTGCCACGACTGGGACACGCTGCTAGAATCTTCCAGCGATACGCTGTTGAAACACTGGAGTTTGAAATTCAGCCAATGTGCCCCGCAAACACGGGCGGTGGTTACGTTGCTGGCTTCCTGCCTGATCCAACTGACAACGATCACACCTTCGACGCGCTTCAAGCAACTCGTGGTGCAGTCGTTGCCAAATGGTGGGAAAGCAGAACAGTCCGACCCCAGTACACCCGCACGCTCCTCTGGACCTCGTCGGGAAAGGAGCAGCGTCTCACGTCACCTGGTCGGCTGATACTCCTGTGTGTCGGCAACAACACTGACGTGGTCAACGTGTCGGTGCTGTGCCGCTGGAGTGTTCGACTGAGCGTTCCATCTCTTGAGACACCTGAGGAGACCACCGCTCCCATCATGACACAAGGTTCCCTGTACAACGATTCCCTTTCCACAAATGACTTCAAGTCCATCCTCCTAGGATCCACACCACTGGACATTGCCCCTGATGGAGCAGTCTTCCAGCTGGACCGTCCGCTGTCCATTGACTACAGCCTTGGAACCGGAGATGTTGACCGTGCTGTTTACTGGCACCTCAAGAAGTTTGCTGGAAATGCTGGCACACCTGCAGGCTGGTTTCGCTGGGGCATCTGGGACAACTTCAACAAGACGTTCACAGATGGCGTTGCTTACTACTCTGATGAGCAGCCTCGTCAAATCCTGCTGCCTGTTGGCACTGTCTGCACCAGGGTTGACTCGGAAAACTAA

>Singapore....Grouper (Greasy)......1991....Cell....AF281657

ATGGTACGCAAAGGTGAGAAGAAATTGGCAAAACCCGCGACCACCAAGGCCGCGAATCCGCAACCCCGCCGACGTGCTAACAATCGTCGGCGTAGTAATCGCACTGACGCACCTGTGTCTAAGGCCTCGACTGTAACTGGATTCGGACGTGGGACCAATGACGTCCATCTCTCAGGTATGTCGAGAATCTCCCAGGCCGTCCTCCCAGCCGGGACAGGAACAGACGGATACGTTGTTGTTGACGCAACCATCGTCCCCGACCTCCTGCCACGACTGGGACACGCTGCTAGAATCTTCCAGCGATACGCTGTTGAAACACTGGAGTTTGAAATTCAGCCAATGTGCCCCGCAAACACGGGCGGTGGTTACGTTGCTGGCTTCCTGCCTGATCCAACTGACAACGATCACACCTTCGACGCGCTTCAAGCAACTCGTGGTGCAGTCGTTGCCAAATGGTGGGAAAGCAGAACAGTCCGACCTCAGTACATTCGCACGCTCCTCTGGACCTCGTCGGGAAAGGAGCAGCGTCTCACGTCGCCTGGTCGGCTGATACTCCTGTGTGTCGGCAACAACACTGATGTGGTCAACGTGTCAGTGCTGTGTCGCTGGAGTGTTCGACTGAGCGTTCCATCTCTTGAGACACCTGAAGAGACCACCGCTCCCATCATGACACAAGGTTCCCTGTACAACGATTCCCTTTCCACAACTGACTTCAAGTCCATCCTCCTAGGATCCACACCACTGGACATTGCCCCTGATGGAGCAGTCTTCCAGCTGGACCGTCCGCTGTCCATTGACTACAGCCTTGGAACTGGAGATGTTGACCGTGCTGTTTATTGGCACCTCAAGAAGTTTGCTGGAAATGCTGGCACACCTGCAGGCTGGTTTCGCTGGGGCATCTGGGACAACTTCAACAAGACGTTCACAGATGGCGTTGCATACTACTCTGATGAGCAGCCCCGTCAAATCCTGCTGCCTGTTGGCACTGTCTGCACCAGGGTTGACTCGGAAAACTAA

>Singapore....Grouper (Greasy)......2001....Cell....AF318942

ATGGTACGCAAAGGTGAGAAGAAATTGGCAAAACCCGCGACCACCAAGGCCGCGAATCCGCAACCCCGCCGACGTGCTAACAATCGTCGGCGTAGTAATCGCACTGACGCACCTGTGTCTAAGGCCTCGACTGTAACTGGATTCGGACGTGGGACCAATGACGTCCATCTCTCAGGTATGTCGAGAATCTCCCAGGCCGTCCTCCCAGCCGGGACAGGAACAGACGGATACGTTGTTGTTGACGCAACCATCGTCCCCGACCTCCTGCCACGACTGGGACACGCTGCTAGAATCTTCCAGCGATACGCTGTTGAAACACTGGAGTTTGAAATTCAGCCAATGTGCCCCGCAAACACGGGCGGTGGTTACGTTGCTGGCTTCCTGCCTGATTCAACTGACAACGATCACACCTTCGACGCGCTTCAAGCAACTCGTGGTGCAGTCGTTGCCAAATGGTGGGAAAGCAGAACAGTCCGACCTCAGTACACCCGCACGCTCCTCTGGACCTCGTCGGGAAAGGAGCAGCGTCTCACGTCACCTGGTCGGCTGATACTCCTGTGTGTCGGCAACAACACTGATGTGGTCAACGTGTCAGTGCTGTGTCGCTGGAGTGTTCGACTGAGCGTTCCATCTCTGGAGACACCTGAAGAGACCACCGCTCCCATCATGACACAAGGTTCCCTGTACAACGATTCCCTTTCCACAAATGACTTCAAGTCCATCCTCCTAGGATCCACACCACTGGACATTGCCCCTGATGGAGCAGTCTTCCAGCTGGACCGTCCGCTGTCCATTGACTACATCCTTGGAACTGGAGATGTTGACCGTGCTGTTTATTGGCACCTTAAGAAGTTTGCTGGAAATGCTGGCACACCTGCAGGCTGGTTTCGCTGGGGCATCTGGGACAACTTTAATAAGACGTTCACAGATGGCGTTGCTTACTACTCTGATGAGCAGCCCCGTCAAATCCTGCTGCCTGTTGGCACTGTCTGCACCAGGGTTGACTCGGGAAACTAA

>Singapore....Guppy.................2000....Cell....AF499774

ATGGTACGCAAAGGTGATAAGAAATTGGCAAAACCCGCGACCACCAAGGCCGCGAATCCGCATCCCCGCCGACGTGCTAACAATCGTCGGCGTAGTAATCGCACTGACGCACCTGTGTCTAAGGCCTCGACTGTAACTGGATTCGGACGTGGGACCAATGAAGTCTATCTCTCAGGTATGTCGAGAATCTCCCAGGCCGTCCTCCCAGCCGGGACAGGAACAGACGGATACGTTGTTGTTGACGCAACCATCGTCCCCGACCTCCTGCCACGACTGGGACACGCTGCTAGAATCTTCCAGCGATACGCTGTTGAAACACTGGAGTTTGAAATTCAGCCAATGTGCCCCGCAAACACGGGCGGTGGTTACGTTGCTGGCTTCCTGCCTGATCCAACTGACAACGATCACACCTTCGACGCGCTTCAAGCAACTCGTGGTGCAGTCGTTGCCAAATGGTGGGAAAGCAGAACAGTCCGACCTCAGTACACCCGCACGCTCCTCTGGACCTCGTCGGGAAAGGAGCAGCGTCTCATGTCACCTGGTCGGCTGATACTCCTGTGTGTCGGCAACAACACTGATGTGGTCAACGTGTCAGTGCTGTGTCGCTGGAGTGTTCGACTGAGCGTTCCATCTCTTGAGACACCTGAAGAGACCACCGCTCCCATCATGACACAAGGTTCCCTGTACAACGATTCCCTTTCCACAACTGACTTCAAGTCCATCCTTCTAGGATCCACACCACTGGACATTGCCCCTGATGGAGCAGTCTTCCAGCTGGACCGTCCGCTGTCCATTGACTACACCCTTGGAACTGGAGATGTTGACCGTGCTGTTTATTGGCACCTCAAGAAGTTTGCTGGAAATGCTAGCACACCTGCAGGCTGGTTTCGCTGGGGCATCTGGGACAACTTCAACAAGACGTTCACAGATGGCGTTGCCTACTACTCTGATGAGCAGCCCCGTCAAATCCTGCTGCCTGTTGGCACTGTCTGCATCAGGGTTGACTCGGAAAACTAA

>Vietnam......Grouper (Orange spot).2007....Fish....EF492143

ATGGTACGCAAAGGTGAGAAGAAATTGGCAAAACCCGCGACCACCAAGGCCGCGAATCCGCAACCCCGCCGACGTGCTAACAATCGTCGGCGTAGTAATCGCACTGACGCACCTGTGTCTAAGGCCTCGACTGTGACTGGATTTGGACGTGGGACCAATGACGTCCATCTCTCAGGTATGTCGAGAATCTCCCAGGCCGTCCTCCCAGCCGGGACAGGAACTGACGGATACGTCGTTGTTGACGCAACCATCGTCCCCGACCTCCTGCCACGACTGGGACACGCTGCTAGAATCTTCCAGCGATACGCTGTTGAAACACTGGAGTTTGAAATTCAGCCAATGTGCCCCGCAAACACGGGCGGTGGTTACGTTGCTGGCTTCCTGCCTGATCCAACTGACAACGACCACACCTTCGACGCGCTTCAAGCAACTCGTGGTGCAGTCGTTGCCAAATGGTGGGAAAGCAGAGCAGTCCGACCTCAGTACACCCGTACGCTCCTCTGGACCTCGTCGGGAAAGGAGCAGCGTCTCACGTCACCTGGTCGGCTGATACTCCTGTGTGTCGGCAACAACACTGATGTGGTCAACGTGTC-GTGCTGTGTCGCTGGAGTGTTCGACTGAGCGTTCCATCTCTTGAGACACCTGAAGAGACCACCGCTCCCATCATGACACAAGGTTCCCTGTACAACGATTCCCTTTCCACAAATGACTTCAAGTCCATCCTCCTAGGATCCACACCACTGGATATTGCCCCTGATGGAGCAGTCTTCCAGCTGGACCGTCCGCTGTCCATTGACTACAGCCTTGGAACTGGAGATGTTGACCGTGCTGTTTATTGGCACCTCAAGAAGTTTGCTGGAAATGCTGGCACACCTGCAGGCTGGTTTCGCTGGGGCATCTGGGACAACTTCAATAAGACGTTCACAGATGGCGTTGCCTACTACTCTGATGAGCAGCCCCGTCAAATCCTGCTGCCTGTTGGCACTGTCTGCACCAGGGTTGACTCGGAAAACTAA

>Vietnam......Grouper (Humpback)....2015....Fish....USC Vie1

ATGGTACGCAAAGGTGAGAAGAAATTGGCAAAACCCGCGACCACCAAGGCCGCGAATCCGCAACCCCGCCGACGTGCTAACAATCGTCGGCGTAGTAATCGCACTGACGCACCTGTGTCTAAGGCCTCGACTGTGACTGGATTTGGACGTGGGACCAATGACGTCCATCTCTCAGGTATGTCGAGAATCTCCCAGGCCGTCCTCCCAGCCGGGACAGGAACTGACGGATACGTCGTTGTTGACGCAACCATCGTCCCCGACCTCCTGCCACGACTGGGACACGCTGCTAGAATCTTCCAGCGATACGCTGTTGAAACACTGGAGTTTGAAATTCAACCAATGTGCCCCGCAAACACGGGCGGTGGTTACGTTGCTGGCTTCCTGCCTGATCCAACTGACAACGACCACACCTTCGACGCGCTTCAAGCAACTCGTGGTGCAGTCGTTGCCAAATGGTGGGAAAGCAGAACAGTCCGACCTCAGTACACCCGTACGCTCCTCTGGACCTCGTCGGGAAAGGAGCAGCGTCTCACGTCACCTGGTCGGCTGATACTCCTGTGTGTCGGCAACAACACTGATGTGGTCAACGTGTCAGTGCTGTGTCGCTGGAGTGTTCGACTGAGCGTTCCATCTCTTGAGACACCTGAAGAGACCACCGCTCCCATCATGACACAAGGTTCCCTGTACAACGATTCCCTTTCCACAAATGACTTCAAGTCCATCCTCCTAGGATCCACGCCACTGGATATTGCCCCTGATGGAGCAGTCTTCCAGCTGGACCGTCCGCTGTCCATCGACTACAGCCTTGGAACTGGAGATGTTGATCGTGCTGTTTATTGGCACCTCAAGAAGTTTGCTGGAAATGCTGGCACACCTGCAGGCTGGTTTCGCTGGGGCATCTGGGACAACTTCAATAAGACGTTCACAGATGGCGTTGCCTACTACTCTGATGAGCAGCCCCGTCAAATCCTGCTGCCTGTTGGCACTGTCTGCACCAGGGTTGACTCGGAAAACTAA

>Vietnam......Grouper (Humpback)....2015....Fish....USC Vie2

ATGGTACGCAAAGGTGAGAAGAAATTGGCAAAACCCGCGACCACCAAGGCCGCGAATCCGCAACCCCGCCGACGTGCTAACAATCGTCGGCGTAGTAATCGCACTGACGCACCTGTGTCTAAGGCCTCGACTGTGACTGGATTTGGACGTGGGACCAATGACGTCCATCTCTCAGGTATGTCGAGAATCTCCCAGGCCGTCCTCCCAGCCGGGACAGGAACTGACGGATACGTCGTTGTTGACGCAACCATCGTCCCCGACCTCCTGCCACGACTGGGACACGCTGCTAGAATCTTCCAGCGATACGCTGTTGAAACACTGGAGTTTGAAATTCAACCAATGTGCCCCGCAAACACGGGCGGTGGTTACGTTGCTGGCTTCCTGCCTGATCCAACTGACAACGACCACACCTTCGACGCGCTTCAAGCAACTCGTGGTGCAGTCGTTGCCAAATGGTGGGAAAGCAGAACAGTCCGACCTCAGTACACCCGTACGCTCCTCTGGACCTCGTCGGGAAAGGAGCAGCGTCTCACGTCACCTGGTCGGCTGATACTCCTGTGTGTCGGCAACAACACTGATGTGGTCAACGTGTCAGTGCTGTGTCGCTGGAGTGTTCGACTGAGCGTTCCATCTCTTGAGACACCTGAAGAGACCACCGCTCCCATCATGACACAAGGTTCCCTGTACAACGATTCCCTTTCCACAAATGACTTCAAGTCCATCCTCCTAGGATCCACGCCACTGGATATTGCCCCTGATGGAGCAGTCTTCCAGCTGGACCGTCCGCTGTCCATCGACTACAGCCTTGGAACTGGAGATGTTGATCGTGCTGTTTATTGGCACCTCAAGAAGTTTGCTGGAAATGCTGGCACACCTGCAGGCTGGTTTCGCTGGGGCATCTGGGACAACTTCAATAAGACGTTCACAGATGGCGTTGCCTACTACTCTGATGAGCAGCCCCGTCAAATCCTGCTGCCTGTTGGCACTGTCTGCACCAGGGTTGACTCGGAAAACTAA

>Vietnam......Grouper (Humpback)....2015....Fish....USC Vie3

ATGGTACGCAAAGGTGAGAAGAAATTGGCAAAACCCGCGACCACCAAGGCCGCGAATCCGCAACCCCGCCGACGTGCTAACAATCGTCGGCGTAGTAATCGCACTGACGCACCTGTGTCTAAGGCCTCGACTGTGACTGGATTTGGACGTGGGACCAATGACGTCCATCTCTCAGGTATGTCGAGAATCTCCCAGGCCGTCCTCCCAGCCGGGACAGGAACTGACGGATACGTCGTTGTTGACGCAACCATCGTCCCCGACCTCCTGCCACGACTGGGACACGCTGCTAGAATCTTCCAGCGATACGCTGTTGAAACACTGGAGTTTGAAATTCAACCAATGTGCCCCGCAAACACGGGCGGTGGTTACGTTGCTGGCTTCCTGCCTGATCCAACTGACAACGACCACACCTTCGACGCGCTTCAAGCAACTCGTGGTGCAGTCGTTGCCAAATGGTGGGAAAGCAGAACAGTCCGACCTCAGTACACCCGTACGCTCCTCTGGACCTCGTCGGGAAAGGAGCAGCGTCTCACGTCACCTGGTCGGCTGATACTCCTGTGTGTCGGCAACAACACTGATGTGGTCAACGTGTCAGTGCTGTGTCGCTGGAGTGTTCGACTGAGCGTTCCATCTCTTGAGACACCTGAAGAGACCACCGCTCCCATCATGACACAAGGTTCCCTGTACAACGATTCCCTTTCCACAAATGACTTCAAGTCCATCCTCCTAGGATCCACGCCACTGGATATTGCCCCTGATGGAGCAGTCTTCCAGCTGGACCGTCCGCTGTCCATCGACTACAGCCTTGGAACTGGAGATGTTGATCGTGCTGTTTATTGGCACCTCAAGAAGTTTGCTGGAAATGCTGGCACACCTGCAGGCTGGTTTCGCTGGGGCATCTGGGACAACTTCAATAAGACGTTCACAGATGGCGTTGCCTACTACTCTGATGAGCAGCCCCGTCAAATCCTGCTGCCTGTTGGCACTGTCTGCACCAGGGTTGACTCGGAAAACTAA

>Vietnam......Grouper (Humpback)....2015....Fish....USC Vie4

ATGGTACGCAAAGGTGAGAAGAAATTGGCAAAACCCGCGACCACCAAGGCCGCGAATCCGCAACCCCGCCGACGTGCTAACAATCGTCGGCGTAGTAATCGCACTGACGCACCTGTGTCTAAGGCCTCGACTGTGACTGGATTTGGACGTGGGACCAATGACGTCCATCTCTCAGGTATGTCGAGAATCTCCCAGGCCGTCCTCCCAGCCGGGACAGGAACTGACGGATACGTCGTTGTTGACGCAACCATCGTCCCCGACCTCCTGCCACGACTGGGACACGCTGCTAGAATCTTCCAGCGATACGCTGTTGAAACACTGGAGTTTGAAATTCAACCAATGTGCCCCGCAAACACGGGCGGTGGTTACGTTGCTGGCTTCCTGCCTGATCCAACTGACAACGACCACACCTTCGACGCGCTTCAAGCAACTCGTGGTGCAGTCGTTGCCAAATGGTGGGAAAGCAGAACAGTCCGACCTCAGTACACCCGTACGCTCCTCTGGACCTCGTCGGGAAAGGAGCAGCGTCTCACGTCACCTGGTCGGCTGATACTCCTGTGTGTCGGCAACAACACTGATGTGGTCAACGTGTCAGTGCTGTGTCGCTGGAGTGTTCGACTGAGCGTTCCATCTCTTGAGACACCTGAAGAGACCACCGCTCCCATCATGACACAAGGTTCCCTGTACAACGATTCCCTTTCCACAAATGACTTCAAGTCCATCCTCCTAGGATCCACGCCACTGGATATTGCCCCTGATGGAGCAGTCTTCCAGCTGGACCGTCCGCTGTCCATCGACTACAGCCTTGGAACTGGAGATGTTGATCGTGCTGTTTATTGGCACCTCAAGAAGTTTGCTGGAAATGCTGGCACACCTGCAGGCTGGTTTCGCTGGGGCATCTGGGACAACTTCAATAAGACGTTCACAGATGGCGTTGCCTACTACTCTGATGAGCAGCCCCGTCAAATCCTGCTGCCTGTTGGCACTGTCTGCACCAGGGTTGACTCGGAAAACTAA

>Taiwan.......Grouper (Greasy)......2000....Cell....AF245003

ATGGTACGCAAAGGTGAAAAGAAATTGGCAAAACCCGCGACCACCAAGGCCGCGAATCCGCAACCCCGCCGACGTGCCAACAATCGTCGGCGTAGCAATCGCACTGACGCACCTGTGTCTAAGGCCTCGACTGTAACTGGATTTGGACGTGGGACCAATGACGTCCATCTCTCAGGTATGTCGAGAATCTCCCAGGCCGTCCTCCCAGCCGGGACAGGAACAGACGGATACGTTGTTGTTGATGCAACCATCGTCCCCGACCTCCTGCCACGACTGGGACACGCTGCTAGAATCTTCCAGCGATACGCTGTTGAAGCACTGGAGTTTGAAATTCAGCCAATGTGCCCCGCAAACACGGGCGGTGGTTACGTTGCTGGCTTCCTGCCTGATCCAACTGACAACGATCACACCTTCGACGCGCTTCAAGCAACTCGTGGTGCAGTCGTTGCCAAATGGTGGGAAAGCAGAACAGTCCGACCTCAGTACACCCGCACGCTCCTCTGGACCTCGACGGGAAAGGAGCAGCGTCTCACGTCACCTGGTCGGCTGATACTCCTGTGTGTCGGCAACAACACTGATGTGGTCAACGTGTCAGTGCTGTGTCGCTGGAGTGTTCGACTGAGCGTTCCATCTCTTGAGACACCTGAAGAGACCACCGCTCCCATCATGACACAAGGTTCCCTGTACAACGATTCCCTTTCCACAAATGACTTCAAATCCATCCTCCTAGGATCCACACCACTGGACATTGCCCCTGATGGAGCAGTCTTCCAGCTGGACCGTCCGCTGTCCATTGACTACAGCCTTGGAACTGGAGATGTTGACCGTGCTGTTTACTGGCACCTCAAGAAGTTTGCTGGAAATGCTGGCACACCTGCAGGCTGGTTTCGCTGGGGCATCTGGGACAACTTCAACAAGACGTTCACAGATGGCGTTGCCTACTACTCTGATGAGCAGCCCCGTCAAATCCTGCTGCCTGTTGGCACTGTCTGCACCAGGGTTGACTCGGAAAACTAA

>Taiwan.......Grouper (Giant).......2000....Cell....AF245004

ATGGTACGCAAAGGTGAAAAGAAATTGGCAAAACCCCCGACCACCAAGGCCGCGAATCCGCAACCCCGCCGACGTGCTAACAATCGTCGGCGTAGTAATCGCACTGACGCGCCTGTGTCTAAGGCCTCGACTGTGACTGGATTTGGACGTGGGACCAATGACGTCCATCTCTCAGGTATGTCGAGAATCTCCCAGGCCGTCCTCCCAGCCGGGACAGGAACAGACGGATACGTTGTTGTTGACGCAACCATCGTCCCCGACCTCCTGCCACGACTGGGACACGCTGCTAGAATCTTCCAGCGATACGCTGTTGAAACACTGGAGTTTGAAATTCAGCCAATGTGCCCCGCAAACACGGGCGGTGGTTACGTTGCTGGCTTCCTGCCTGATCCAACTGACAACGACCACACCTTCGACGCGCTTCAAGCAACTCGTGGTGCAGTCGTTGCCAAATGGTGGGAAAGCAGAACAGTCCGACCTCAGTACACCCGCACGCTCCTCTGGACCTCGTCGGGAAAGGAGCAGCGTCTCACGTCACCTGGTCGGCTGATACTCCTGTGTGTCGGCAACAACACTGATGTGGTCAACGTGTCAGTGCTGTGTCGCTGGAGTGTTCGACTGAGCGTTCCATCTCTTGAGACACCTGAAGAGACCACCGCTCCCATCATGACACAAGGTTCCCTGTACAACGATTCCCTTTCCACAAATGACTTTAAGTCCATCCTCCTAGGATCCACACCACTGGACATTGCCCCTGATGGAGCAGTCTTCCAGCTGGATCGACCGCTGTCCATTGACTACAGCCTTGGAACTGGAGATGTTGACCGTGCTGTTTACTGGCACCTCAAGAAGTTTGCTGGAAATGCTGGCACACCTGCAGGCTGGTTTCGCTGGGGCATCTGGGACAACTTCAACAAGACGTTCACAGATGGCGTTGCCTACTACTCTGATGAGCAGCCCCGTCAAATCCTGCTGCCTGTTGGCACTGTCTGCACCAGGGTTGACTCGGAAAACTAA

>Taiwan.......Grouper species.......1995....Cell....AY690596

ATGGTACGCAAAGGTGAGAAGAAATTGGCAAAACCCGCGACCACCAAGGCCGCGAATCCGCAACCCCGCCGACGTGCTAACAATCGTCGGCGTAGTAATCGCACTGACGCACCTGTGTCTAAGGCCTCGACTGTAACTGGATTTGGACGTGGGACCAATGACGTCCATCTCTCAGGTATGTCGAGAATCTCCCAGGCCGTCCTCCCAGCCGGGACAGGAACAGACGGATACGTTGTTGTTGACGCAACCATCGTTCCCGACCTCCTGCCACGACTGGGACACGCTGCTAGAATCTTCCAGCGATACGCTGTTGAAACACTGGAGTTTGAAATTCAGCCAATGTGCCCCGCAAACACGGGCGGTGGTTACGTTGCTGGCTTCCTGCCTGATCCAACTGACAACGACCACACCTTCGACGCGCTTCAAGCAACTCGTGGTGCAGTCGTTGCCAAATGGTGGGAAAGCAGAACAGTCCGACCTCAGTACACCCGCACGCTCCTCTGGACCTCGTCGGGAAAGGAGCAGCGTCTCACGTCACCTGGTCGGCTGATACTCCTGTGTGTCGGCAACAACACTGATGTGGTCAACGTGTCAGTGCTGTGCCGCTGGAGTGTTCGACTGAGCGTTCCATCTCTTGAGACACCTGAAGAGACCACCGCTCCCATCATGACACAAGGTTCCCTGTACAACGATTCCCTTTCCACAAATGACTTCAAGTCCATCCTCCTAGGATCCACACCACTGGACATTGCCCCTGATGGAGCAGTCTTCCAGCTGGACCGTCCGCTGTCCATTGACTACAGCCTTGGAACTGGAGATGTTGACCGTGCTGTCTATTGGCACCTCAAGAAGTTTGCTGGAAATGCTGGCACACCTGCAGGCTGGTTTCGCTGGGGCATCTGGGACAACTTCAACAAGACGTTCGCAGATGGCGTTGCCTACTACTCTGATGAGCAGCCTCGTCAAATCCTGCTGCCTGTTGGCACTGTCTGCACCAGGGTTGACTCGGAAAACTAA

>Taiwan.......Lutjanus..............2004....Unknown.AY835642

ATGGTACGCAAAGGTGAGAAGAAATTGGCAAAACCCGCGACCACCAAGGCCGCAAATCCGCAACCCCGCCGACGTGCTAACAATCGTCGGCGTAGTAATCGCACTGACGCACCTGTGTCTAAGGCCTCGACTGTGACTGGATTTGGACGTGGGACCAATGACGTCCATCTCTCAGGTATGTCGAGAATCTCCCAGGCCGTCCTCCCAGCCGGGACAGGAACTGACGGATACGTTGTTGTTGACGCAACCATCGTCCCCGACCTCCTGCCACGACTGGGACACGCTGCTAGAATCTTCCAGCGATACGCTGTTGAAACACTGGAGTTTGAAATTCAGCCAATGTGCCCCGCAAACACGGGCGGTGGTTACGTTGCTGGCTTCCTGCCTGATCCAACTGACAACGAACACACCTTCGACGCGCTTCAAGCAACTCGTGGTGCAGTCGTTGCCAAATGGTGGGAAAGCAGAACAGTCCGACCCCAGTACACCCGTACGCTCCTCTGGACCTCGTCGGGAAAGGAGCAGCGTCTCACGTCACCTGGTCGGCTGATACTCCTGTGTGTCGGCAACAACACTGATGTGGTCAACGTGTCGGTGCTGTGTCGCTGGAGTGTTCGATTGAGCGTTCCATCTCTTGAGACACCTGAAGAGACCACCGCTCCCATCATGACACAAGGTTCCCTGTACAACGATTCCCTTTCCACAAATGACTTCAAGTCCATCCTCCTAGGATCCACACCACTGGATATTGCCCCTGATGGAGCAGTCTTCCAGCTGGACCGTCCGCTGTCCATTGACTACAGCCTTGGAACTGGAGATGTTGACCGTGCTGTTTATTGGCACCTCAAGAAGTTTGCTGGAAATGCTGGCACACCTGCAGGCTGGTTTCGCTGGGGCATCTGGGACAACTTCAATAAGACGTTCACAGATGGCGTTGCCTACTACTCTGATGAGCAGCCCCGCCAAATCCTGCTGCCTGTTGGCACTGTCTGCACCAGGGTTGACTCGGAAAACTAA

>Taiwan.......Grouper (Red spot)....2003....Cell....EU391590

ATGGTACGCAAAGGTGAGAAGAAATTGGCAAAACCCGCGACCACCAAGGCCGCGAATCCGCAACCCCGCCGACGTGCTAACAATCGTCGGCGTAGTAATCGCACTGACGCACCTGTGTCTAAAGCCTCGACTGTAACTGGGTTTGGACGTGGGACCAATGACGTCCATCTCTCAGGTATGTCGAGAATCTCCCAGGCCGTCCTCCCAGCCGGGACAGGAACAGACGGATACGTTGTTGTTGACGCAACCATCGTCCCCGACCTCCTGCCACGACTGGGACACGCTGCTAGAATCTTCCAGCGATACGCTGTTGAAACACTGGAGTTTGAAATTCAGCCAATGTGCCCCGCAAACACGGGCGGTGGTTACGCTGCTGGCTTCTTGCCTGATCCAACTGACAACGATCACACCTTCGACGCGCTTCAAGCAACTCGTGGTGCAGTCGTTGCCAAATGGTGGGAAAGCAGAACAGTCCGACCTCAGTACACCCGTACGCTCCTCTGGACCTCGTCGGGAAAGGAGCAGCGTCTCACGTCACCTGGTCGGCTGATACTCCTGTGTGTCGGCAACAACACTGATGTGGTCAACGTGTCAGTGCTGTGTCGCTGGAGTGTTCGACTGAGCGTTCCATCTCTTGAGACACCTGAAGAGACCACCGCTCCCATCATGACACAAGGTTCCCTGTACAACGATTCCCTTTCCACAAATGACTTCAAGTCCATCCTCCTAGGATCCATACCACTGGACATTGCCCCTGATGGATCAGTCTTCCAGCTGGACCGCCCGCTGTCCATTGACTACAGCCTTGGAACTGGAGATGTTGACCGTGCTGTTTACTGGCACCTCAAGAAGTTTGCTGGAAATGCTAGCACACCTGCAGGCTGGTTTCGCTGGGGCATCTGGGACAATTTCAACAAGACGTTTACAGATGGCGTTGCTTACTACTCTGATGAGCAGCCTCGTCAAATCCTGCTGCCTGTTGGCACTGTCTGCACCAGGGTTGACTCGGGAAACTAA

>Taiwan.......Grouper (Giant-Tiger).2015....Fish....USC Tai1

ATGGTACGCAAAGGTGAGAAGAAATTGGCAAAACCCGCGACCACCAAGGCCGCGAATCCCCAACCCCGCCGACGTGCTAACAATCGTCGGCGTAGTAATCGCACTGACGCACCTGTGTCTAAGGCCTCGACTGTGACTGGATTTGGACGTGGGACCAATGACGTCCATCTCTCAGGTATGTCGAGAATCTCTCAGGCCGTCCTCCCAGCCGGGACAGGAACTGACGGATACGTCGTTGTTGACGCAACCATCGTCCCCGACCTCCTGCCACGACTGGGACACGCTGCTAGAATCTTCCAGCGATACGCTGTTGAAACACTGGAGTTTGAAATTCAGCCAATGTGCCCCGCAAACACGGGCGGTGGTTACGTTGCTGGCTTCCTGCCTGATCCAACTGACAACGACCACACCTTCGACGCGCTTCAAGCAACTCGTGGTGCAGTCGTTGCCAAATGGTGGGAAAGCAGAACAGTCCGACCTCAGTACACCCGTACGCTCCTCTGGACCTCGTCGGGAAAGGAGCAGCGTCTCACGTCACCTGGTCGGCTGATACTCCTGTGTGTTGGCAACAACACTGATGTGGTCAACGTGTCAGTGCTGTGTCGCTGGAGTGTTCGACTGAGCGTTCCATCTCTTGAGACACCTGAAGAGACCACCGCTCCCATCATGACACAAGGTTCCCTGTACAACGATTCCCTTTCCACAAATGACTTCAAGTCCGTCCTCCTAGGATCCACACCACTGGATATTGCCCCTGATGGAGCAGTCTTCCAGCTGGACCGTCCGCTGTCCATTGACTACAGCCTTGGAACTGGAGATGTTGATCGTGCTGTTTATTGGCACCTCAAGAAGTTTGCTGGAAATGCTGGCACACCTGCAGGCTGGTTTCGCTGGGGCATCTGGGACAACTTCAATAAGACGTTCACAGATGGCGTTGCCTACTACTCTGATGAGCAGCCCCGTCAAATCCTGCTGCCTGTTGGCACTGTCTGCACCAGGGTTGACTCGGAAAACTAA

>Taiwan.......Grouper (Giant-Tiger).2015....Fish....USC Tai2

ATGGTACGCAAAGGTGAGAAGAAATTGGCAAAACCCGCGACCACCAAGGCCGCGAATCCCCAACCCCGCCGACGTGCTAACAATCGTCGGCGTAGTAATCGCACTGACGCACCTGTGTCTAAGGCCTCGACTGTGACTGGATTTGGACGTGGGACCAATGACGTCCATCTCTCAGGTATGTCGAGAATCTCTCAGGCCGTCCTCCCAGCCGGGACAGGAACTGACGGATACGTCGTTGTTGACGCAACCATCGTCCCCGACCTCCTGCCACGACTGGGACACGCTGCTAGAATCTTCCAGCGATACGCTGTTGAAACACTGGAGTTTGAAATTCAGCCAATGTGCCCCGCAAACACGGGCGGTGGTTACGTTGCTGGCTTCCTGCCTGATCCAACTGACAACGACCACACCTTCGACGCGCTTCAAGCAACTCGTGGTGCAGTCGTTGCCAAATGGTGGGAAAGCAGAACAGTCCGACCTCAGTACACCCGTACGCTCCTCTGGACCTCGTCGGGAAAGGAGCAGCGTCTCACGTCACCTGGTCGGCTGATACTCCTGTGTGTTGGCAACAACACTGATGTGGTCAACGTGTCAGTGCTGTGTCGCTGGAGTGTTCGACTGAGCGTTCCATCTCTTGAGACACCTGAAGAGACCACCGCTCCCATCATGACACAAGGTTCCCTGTACAACGATTCCCTTTCCACAAATGACTTCAAGTCCGTCCTCCTAGGATCCACACCACTGGATATTGCCCCTGATGGAGCAGTCTTCCAGCTGGACCGTCCGCTGTCCATTGACTACAGCCTTGGAACTGGAGATGTTGATCGTGCTGTTTATTGGCACCTCAAGAAGTTTGCTGGAAATGCTGGCACACCTGCAGGCTGGTTTCGCTGGGGCATCTGGGACAACTTCAATAAGACGTTCACAGATGGCGTTGCCTACTACTCTGATGAGCAGCCCCGTCAAATCCTGCTGCCTGTTGGCACTGTCTGCACCAGGGTTGACTCGGAAAACTAA

>Taiwan.......Grouper (Giant-Tiger).2015....Fish....USC Tai3

ATGGTACGCAAAGGTGAGAAGAAATTGGCAAAACCCGCGACCACCAAGGCCGCGAATCCCCAACCCCGCCGACGTGCTAACAATCGTCGGCGTAGTAATCGCACTGACGCACCTGTGTCTAAGGCCTCGACTGTGACTGGATTTGGACGTGGGACCAATGACGTCCATCTCTCAGGTATGTCGAGAATCTCTCAGGCCGTCCTCCCAGCCGGGACAGGAACTGACGGATACGTCGTTGTTGACGCAACCATCGTCCCCGACCTCCTGCCACGACTGGGACACGCTGCTAGAATCTTCCAGCGATACGCTGTTGAAACACTGGAGTTTGAAATTCAGCCAATGTGCCCCGCAAACACGGGCGGTGGTTACGTTGCTGGCTTCCTGCCTGATCCAACTGACAACGACCACACCTTCGACGCGCTTCAAGCAACTCGTGGTGCAGTCGTTGCCAAATGGTGGGAAAGCAGAACAGTCCGACCTCAGTACACCCGTACGCTCCTCTGGACCTCGTCGGGAAAGGAGCAGCGTCTCACGTCACCTGGTCGGCTGATACTCCTGTGTGTTGGCAACAACACTGATGTGGTCAACGTGTCAGTGCTGTGTCGCTGGAGTGTTCGACTGAGCGTTCCATCTCTTGAGACACCTGAAGAGACCACCGCTCCCATCATGACACAAGGTTCCCTGTACAACGATTCCCTTTCCACAAATGACTTCAAGTCCGTCCTCCTAGGATCCACACCACTGGATATTGCCCCTGATGGAGCAGTCTTCCAGCTGGACCGTCCGCTGTCCATTGACTACAGCCTTGGAACTGGAGATGTTGATCGTGCTGTTTATTGGCACCTCAAGAAGTTTGCTGGAAATGCTGGCACACCTGCAGGCTGGTTTCGCTGGGGCATCTGGGACAACTTCAATAAGACGTTCACAGATGGCGTTGCCTACTACTCTGATGAGCAGCCCCGTCAAATCCTGCTGCCTGTTGGCACTGTCTGCACCAGGGTTGACTCGGAAAACTAA

>Taiwan.......Grouper (Giant).......2013....Cell....KM588181

ATGGTACGCAAAGGTGAGAAGAAATTGGCAAAACCCGCGACCACCAAGGCCGCGAATCCGCAACCCCGCCGACGTGCTAACAATCGTCGGCGTAGTAATCGCACTGACGCACCTGTGTCTAAGGCCTCGACTGTGACTGGATTTGGACGTGGGACCAATGACGTCCATCTCTCAGGTATGTCGAGAATCTCCCAGGCCGTCCTCCCAGCCGGGACAGGAACTGACGGATACGTCGTTGTTGACGCAACCATCGTCCCCGACCTCCTGCCACGACTGGGACACGCTGCTAGAATCTTCCAGCGATACGCTGTTGAAACACTGGAGTTTGAAATTCAGCCAATGTGCCCCGCAAACACGGGCGGTGGTTACGTTGCTGGCTTCCTGCCTGATCCAACTGACAACGACCACACCTTCGACGCGCTTCAAGCAACTCGTGGTGCAGTCGTTGCCAAATGGTGGGAAAGCAGAACAGTCCGACCTCAGTACACCCGTACGCTCCTCTGGACCTCGTCGGGAAAGGAGCAGCGTCTCACGTCACCTGGTCGGCTGATACTCCTGTGTGTCGGCAACAACACTGATGTGGTCAACGTGTCAGTGCTGTGTCGCTGGAGTGTTCGACTGAGCGTTCCATCTATTGAGACACCTGAAGAGACCACCGCACCCATCATGACACAAGGTTCCCTGTACAACGATTCCCTTTCCACAACTGACTTCAAGTCCATCCTCCTAGGATCCACACCACTGGATATTGCCCCTGATGGAGCAGTCTTCCAGCTGGACCGTCCGCTGTCCATTGACTACAGCCTTGGAACTGGAGATGTTGATCGTGCTGTTTATTGGCACCTCAAGAAGTTTGCTGGAAATGCTGGCACACCTGCAGGCTGGTTTCGCTGGGGCATCTGGGACAACTTCAATAAGACGTTCACAGATGGCGTTGCCTACTACTCTGATGAGCAGCCCCGTCAAATCCTGCTGCCTGTTGGCACTGTCTGCACCAGGGTTGACTCGGAAAACTAA

>Taiwan.......Grouper (Orange spot).2011....Cell....KT071606

ATGGTACGCAAAGGTGAGAAGAAATTGGCAAAACACGCGACCACCAAGGCCGCGAATCCGCAACCCCGCCGACGTGCTAACAATCGTCGGCGTAGTAATCGCACTGACGCACCTGTGTCTAAGGCCTCGACTGTGACTGGATTTGGACGTGGGACCAATGACGTCCATCTCTCAGGTATGTCGAGAATCTCCCAGGCCGTCCTCCCAGCCGGGACAGGAACTGACGGATACGTCGTTGTTGACGCAACGATCGTTCCCGACCTCCTGCCACGACTGGGACACGCTGCTAGAATCTTCCAGCGATACGCTGTTGAAACACTGGAGTTTGAAATTCAGCCAATGTGCCCCGCAAACACGGGCGGTGGTTACGTTGCTGGCTTCCTGCCTGATCCAACTGACAACGACCACACCTTCGACGCGCTTCAAGCAACTCGTGGTGCAGTCGTTGCCAAATGGTGGGAAAGCAGAACAGTCCGACCTCAGTACACCCGTACGCTCCTCTGGACCTCGTCGGGAAAGGAGCAGCGTCTCACGTCACCTGGTCGGCTGATACTCCTATGTGTCGGCAACAACACTGATGTGGTCAACGTGTCGGTGCTGTGTCGCTGGAGTGTTCGACTGAGCGTTCCATCTCTTGAGACACCTGAAGAGACCACCGCTCCCATCATGACACAAGGTTCCCTGTACAACGATTCCCTTTCCACAAATGACTTCAAGTCCATCCTCCTAGGATCCACACCGCTGGATATTGCCCCTGATGGAGCAGTCTTCCAGCTGGACCGTCCGCTGTCCATTGACTACAGCCTTGGAACTGGAGATGTTGACCGTGCTGTTTACTGGCACCTCAAGAAGTTTGCTGGAAATGCTGGCACACCTGCAGGCTGGTTTCGCTGGGGCATCTGGGACAACTTCAATAAGACGTTCACAGATGGCGTTGCCTACTACTCTGATGAGCAGCCCCGTCAAATCCTGCTGCCTGTTGGCACTGTCTGCACCAGGGTTGACTCGGAAAACTAA

>China(S).....Grouper (Orange spot).2003....Unknown.AF534998

ATGGTACGCAAAGGTGAGAAGAAATTGGCAAAACCCGCGACCACCAAGGCCGCGAATCCGCAACCCCGCCGACGTGCTAACAATCGTCGGCGTAGTAATCGCACTGACGCACCTGTGTCTAAGGCCTCGACTGTGACTGGATTTGGACGTGGGACCAATGACGTCCATCTCTCAGGTATGTCGAGAATCTCCCAGGCCGTCCTCCCAGCCGGGACAGGAACTGACGGATACGTTGTCGTTGACGCAACCATCGTCCCCGACCTCCTGCCACGACTGGGACACGCTGCTAGAATCTTCCAGCGATACGCTGTTGAAACACTGGAGTTTGAAATTCAGCCAATGTGCCCCGCAAACACGGGCGGTGGTTACGTTGCTGGCTTCCTGCCTGATCCAACTGACAACGACCACACCTTCGACGCGCTTCAAGCAACTCGTGGTGCAGTCGTTGCCAAATGGTGGGAAAGCAGAACAGTCCGACCTCAGTACACCCGCACGCTCCTCTGGACCTCGTCGGGAAAGGAGCAGCGTCTCACGTCACCTGGTCGGCTGATACTCCTGTGTGTCGGCAACAACACTGATGTGGTCAACGTGTCAGTGCTGTGTCGCTGGAGTGTTCGACTGAGCGTTCCATCTCTTGAGACACCTGAAGAGACCACCGCTCCCATCATGACACAAGGTTCCCTGTACAACGATTCCCTTTCCACAAATGACTTCAAGTCCATCCTCCTAGGATCCACACCACTGGATATTGCCCCTGATGGAGCAGTCTTCCAGCTGGACCGTCCGCTGTCCATTGACTACAGCCTTGGAACTGGAGATGTTGACCGTGCTGTTTATTGGCACCTCAAGAAGTTTGCTGGAAATGCTGGCACACCTGCAGGCTGGTTTCGCTGGGGCATCTGGGACAACTTCAACAAGACGTTCACAGATGGCGTTGCCTACTACTCTGATGAGCAGCCCCGTCAAATCCTGCTGCCTGTTGGCACTGTCTGCACCAGGGTTGACTCGGAAAACTAA

>China(C).....Grouper (Giant).......2004....Unknown.AY721615

ATGGTACGCAAAGGTGAGAAGAAATTGGCAAAACCCGCGACCACCAAGGCCGCGAATCCGCAACCCCGCCGACGTGCTAACAATCGTCGGCGTAGTAACCGCACTGACGCACCTGTGTCTAAGGCCTCGACTGTGACTGGATTTGGACGTGGGACCAATGACGTCCATCTCTCAGGTATGTCGAGAATCTCCCAGGCCGTCCTCCCAGCCGGGACAGGAACAGACGGATACGTTGTTGTTGACGCAACCATCGTCCCCGACCTCCTGCCACGACTGGGACACGCTGCTAGAATCTTCCAGCGATACGCTGTTGAAACACTGGAGTTTGAAATTCAGCCAATGTGCCCCGCAAACACGGGCGGTGGTTACGTTGCTGGCTTCCTGCCTGATCCAACTGACAACGATCACACCTTCGACGCGCTTCAAGCAACTCGTGGTGCAGTCGTTGCCAAATGGTGGGAAAGCAGAACAGTCCGACCTCAGTACACCCGCACGCTCCTCTGGACCTCGTCGGGAAAGGAGCAGCGTCTCACGTCACCTGGTCGGCTGATACTCCTGTGTGTCGGCAACAACACTGACGTGGTCAACGTGTCGGTGCTGTGTCGCTGGAGTGTTCGACTGAGCGTTCCATCTCTTGAGACACCTGAGGAGACCACCGCTCCCATCATGACACAAGGTTCCCTGTACAACGATTCCCTTTCCACAAATGACTTCAAGTCCATCCTCCTAGGATCCACACCACTGGACATTGCCCCTGATGGAGCAGTCTTCCAGCTGGACCGTCCGCTGTCCATTGATTACAGCCTTGGAACTGGAGATGTTGACCGTGCTGTTTACTGGCACCTCAAGAAGTTTGCTGGGAATGCTGGCACACCTGCAGGCTGGTTTCGCTGGGGCATCTGGGACAACTTCAACAAGACGTTCACAGATGGCGTTGCTTACTACTCTGATGAGCAGCCTCGTCAAATCCTGCTGCCTGTTGGCACTGTCTGCACCAGGGTTGACTCGGAAAACTAA

>China(S).....Grouper (Red spot)....2000....Fish....AY744705

ATGGTACGCAAAGGTGAGAAGAAATTGGCAAAACCCGCGACCACTAAGGCCGCGAATCCGCAACCCCGCCGACGTGCTAACAATCGTCGGCGTAGTAATCGCACTGACGCACCTGTGTCTAAGGCCTCGACTGTAACTGGATTTGGACGTGGGACCAATGACGTCCATCTCTCAGGTATGTCGAGAGTCTCCCAGGCCGTCCTCCCAGCCGGGACAGGAACAGACGGATACGTTGTTGTTGATGCAACCATCGTCCCCGACCTCCTGCCACGACTGGGACACGCTGCTAGAATCTTCCAGCGATACGCTGTTGAAACACTGGAGTTTGAAATTCAGCCAATGTGCCCCGCAAACACGGGCGGTGGTTACGTTGCTGGCTTCCTGCCTGATCCAACTGACAACGATCACACCTTCGACGCGCTTCAAGCAACTCGTGGTGCAGTCGTTGCCAAATGGTGGGAAAGCAGAACAGTCCGACCTCAGTACACCCGCACGCTCCTCTGGACCTCGTCGGGAAAGGAGCAGCGTCTCACGTCACCTGGTCGGCTGATACTCCTGTGTGTCGGCAACAACACTGATGTGGTCAACGTGTCAGTGCTGTGTCGCTGGAGTGTTCGACTGAGCGTTCCATCTCTTGAGACACCTGAAGAGACCACCGCTCCCATCATGACACAAGGTTCCCTGTACAACGATTCCCTTTCCACAAATGACTTCAAGTCCATCCTCCTAGGATCCACACCACTGGACATTGCCCCTGATGGAGCAGTCTTCCAGCTGGACCGTCCGCTGTCCATTGACTACAGCCTTGGAACTGGAGATGTTGACCGTGCTGTTTACTGGCACCTCAAGAAGTTTGCTGGAAATGCTGGCACACCTGCAGGCTGGTTTCGCTGGGGCATCTGGGACAACTTCAACAAGACGTTCACAGATGGCGTTGCCTACTACTCTGATGAGCAGCCCCGTCAAATCCTGCTGCCTGTTGGCACTGTCTGCACCAGGGTTGACTCGGAAAACTAA

>China(S).....Grouper (Red spot)....2006....Cell....EF558369

ATGGTACGCAAAGGTGAGAAGAAATTGGCAAAACCCGCGACCACCAAGGCCGCGAATCCGCAACCCCGCCGACGTGCTAACAATCGTCGGCGTAGTAATCGCACTGACGCACCTGTGTCTAAGGCCTCGACTGTAACTGGATTTGGACGTGGGACCAATGACGTCCATCTCTCAGGTATGTCGAGAATCTCCCAGGCCGTCCTCCCAGCCGGGACAGGAACAGACGGATACGTTGTTGTTGACGCAACCATCGTCCCCGACCTCCTGCCACGACTGGGACACGCTGCTAGAATCTTCCAGCGATACGCTGTTGAAACACTGGAGTTTGAAATTCAGCCAATGTGCCCCGCAAACACGGGCGGTGGTTACGTTGCTGGCTTCCTGCCTGATCCAACTGACAACGATCACACCTTCGACGCGCTTCAAGCAACTCGTGGTGCAGTCGTTGCCAAATGGTGGGAAAGCAGAACAGTCCGACCCCAGTACACCCGCACGCTCCTCTGGACCTCGTCGGGAAAGGAGCAGCGTCTCACGTCACCTGGTCGGCTGATACTCCTGTGTGTCGGCAACAACACTGATGTGGTCAACGTGTCAGTGCTGTGTCGCTGGAGTGTTCGATTGAGCGTTCCATCTCTTGAGACACCTGAAGAGACTACCGCTCCCATCATGACACAAGGTTCCCTGTACAACGATTCCCTTTCCACAACTGACTTCAAGTCCATCCTCCTAGGATCCACACCACTGGACATTGCCCCTGATGGAGCAGTTTTCCAGCTGGACCGTCCGCTGTCTATTGACTACAGCCTTGGAACTGGAGATGTTGACCGTGCTGTTTATTGGCACATCAAGAAGTTTGCTGGAAATGCTGGCACACCTGCAGGCTGGTTTCGCTGGGGCATCTGGGACAACTTCAACAAGACGTTCACAGATGGCGTTGCCTACTACTCTGATGAGCAGCCCCGTCAAATTCTGCTGCCTGTTGGCACTGTCTGCACCAGGGTTGACTCGGGAAACTAA

>China(S).....Pompano...............2009....Fish....GU339227

ATGGTACGCAAAGGTGAGAAGAAATTGGCAAAACCCGCGACCACCAAGGCCGCGAATCCGCAACCCCGCCGACGTGCTAACAATCGTCGGCGTAGTAATCGCACTGACGCACCTGTGTCTAAGGCCTCGACTGTGACTGGATTTGGACGTGGGACCAATGACGTCCATCTCTCAGGTATGTCGAGAATCTCCCAGGCCGTCCTCCCAGCCGGGACAGGAACTGACGGATACGTCGTTGTTGACGCAACCATCGTCCCCGACCTCCTGCCACGACTGGGACACGCTGCTAGAATCTTCCAGCGATACGCTGTTGAAACATTGGAGTTTGAAATTCAGCCAATGTGCCCCGCAAACACGGGCGGTGGTTACGTTGCTGGCTTCCTGCCTGATCCAACTGACAACGACCACACCTTCGACGCGCTTCAAGCAACTCGTGGTGCAGTCGTTGCCAAATGGTGGGAAAGCAGAACAGTCCGACCTCAGTACACCCGTACGCTCCTCTGGACCTCGTCGGGAAAGGAGCAGCGTCTCACGTCACCTGGTCGGCTGATACTCCTGTGTGTCGGCAACAACACTGATGTGGTCAACGTGTCGGTGCTGTGTCGCTGGAGTGTTCGACTGAGCGTTCCATCTCTTGAGACACCTGAAGAGACCACCGCTCCCATCATGACACAAGGTCCCCTGTACAACGATTCCCTTTCCACAAATGACTTTAAGTCCATCCTCCTAGGATCCACACCACTGGATATTGCCCCTGATGGAGCAGTCTTCCAGCTGGACCGTCCGCTGTCCATTGACTACAGCCTTGGAACTGGAGATGTTGACCGTGCTGTTTATTGGCACCTCAAGAAGTTTGCTGGAAATGCTGGCACACCTGCAGGCTGGTTTCGCTGGGGCATCTGGGACAACTTCAATAAGACGTTCACAGATGGCGTTGCCTACTACTCTGATGAGCAGCCCCGTCAAATCCTGCTGCCTGTTGGCACTGTCTGCACCAGGGTTGACTCGGAAAACTAA

>China(S).....Sea Perch.............2014....Fish....KP455642

ATGGTACGCAAAGGTGAGAAGAAATTGGCAAAACCCGCGACCACCAAGGCCGCGAATCCGCAACCCCGCCGACGTGCTAACAATCGTCGGCGTAGTAATCGCACTGACGCACCTGTGTCTAAGGCCTCGACTGTGACTGGATTTGGACGTGGGACCAATGACGTCCATCTCTCAGGTATGTCGAGAATCTCCCAGGCCGTCCTCCCAGCCGGGACAGGAACTGACGGATACGTTGTTGTTGACGCAACCATCGTCCCCGACCTCCTGCCACGACTGGGACACGCTGCTAGAATCTTCCAGCGATACGCTGTTGAAACACTGGAGTTTGAAATTCAGCCAATGTGCCCCGCAAACACGGGCGGTGGTTACGTTGCTGGCTTCCTGCCTGATCCAACTGACAACGACCACACCTTCGACGCGCTTCAAGCAACTCGTGGTGCAGTCGTTGCCAAATGGTGGGAAAGCAGAACAGTCCGACCTCAGTACACCCGCACGCTCCTCTGGACCTCGTCGGGAAAGGAGCAGCGTCTCACGTCACCTGGTCGGCTGATACTCCTGTGTGTCGGCAACAACACTGATGTGGTCAACGTGTCGGTGCTGTGTCGCTGGAGTGTTCGACTGAGCGTTCCATCTCTTGAGACACCTGAAGAGACCACCGCTCCCATCATGACGCAAGGTTCCCTGTACAACGATTCCCTTTCCACAAATGACTTCAAGTCCATCCTCCTAGGATCCACACCACTGGATATTGCCCCTGATGGAGCAGTCTTCCAGCTGGACCGTCCGCTGTCCATTGACTACAGCCTTGGAACTGGAGATGTTGATCGTGCTGTTTATTGGCACCTCAAGAAGTTTGCTGGAAATGCTGGCACACCTGCAGGCTGGTTTCGCTGGGGCATCTGGGACAACTTCAACAAGACGTTCACAGATGGCGTTGCCTACTACTCTGATGAGCAGCCCCGTCAAATCCTGCTGCCTGTTGGCACTGTCTGCACCAGGGTTGACTCGGAAAACTAA

>China(N).....Grouper (Giant-Tiger).2013....Unknown.KF668183

ATGGTACGCAAAGGTGAGAAGAAATTGGCAAAACCCGCGACCACCAAGGCCGCGAATCCGCAACCCCGCCGACGTGCTAACAATCGTCGGCGTAGTAATCGCACTGACGCACCTGTGTCTAAGGCCTCGACTGTGACTGGATTTGGACGTGGGACCAATGACGTCCATCTCTCAGGTATGTCGAGAATCTCCCAGGCCGTCCTCCCAGCCGGGACAGGAACTGACGGATACGTCGTTGTTGACGCAACCATCGTCCCCGACCTCCTGCCACGACTGGGACACGCTGCTAGAATCTTCCAGCGATACGCTGTTGAAACATTGGAGTTTGAAATTCAGCCAATGTGCCCCGCAAACACGGGCGGTGGTTACGTTGCTGGCTTCCTGCCTGATCCAACTGACAACGACCACACCTTCGACGCGCTTCAAGCAACTCGTGGTGCAGTCGTTGCCAAATGGTGGGAAAGCAGAACAGTCCGACCTCAGTACACCCGTACGCTCCTCTGGACCTCGTCGGGAAAGGAGCAGCGTCTCACGTCACCTGGTCGGCTGATACTCCTGTGTGTCGGCAACAACACTGATGTGGTCAACGTGTCGGTGCTGTGTCGCTGGAGTGTTCGACTGAGCGTTCCATCTCTTGAGACACCTGAAGAGACCACCGCTCCCATCATGACACAAGGTTCCCTGTACAACGATTCCCTTTCCACAAATGACTTCAAGTCCATCCTCCTAGGATCCACACCACTGGATATTGCCCCTGATGGAGCAGTCTTCCAGCTGGACCGTCCGCTGTCCATTGACTACAGCCTTGGAACTGGAGATGTTGATCGTGCTGTTTATTGGCACCTCAAGAAGTTTGCTGGAAATGCTGGCACACCTGCAGGCTGGTTTCGCTGGGGCATCTGGGACAACTTTAATAAGACGTTCACAGATGGCGTTGCCTACTACTCTGATGAGCAGCCCCGTCAAATCCTGCTGCCTGTTGGCACTGTCTGCACCAGGGTTGACTCGGAAAACTAA

>China(N).....Flounder(Olive).......2006....Unknown.KF841612

ATGGTACGCAAAGGTGAGAAGAAATTGGCAAAACCCGCGACCACCAAGGCCGCGAATCCGCAACCCCGCCGACGTGCTAACAATCGTCGGCGTAGTAATCGCACTGACGCACCTGTGTCTAAGGCCTCGACTGTGACTGGATTTGGACGTGGGACCAATGACGTCCATCTCTCAGGTATGTCGAGAATCTCCCAGGCCGTCCTCCCAGCCGGGACAGGAACTGACGGATACGTCGTTGTTGACGCAACCATCGTCCCCGACCTCCTGCCACGACTGGGACACGCTGCTAGAATCTTCCAGCGATACGCTGTTGAAACACTGGAGTTTGAAATTCAGCCAATGTGCCCCGCAAACACGGGCGGTGGTTACGTTGCTGGCTTCCTGCCTGATCCAACTGACAACGACCACACCTTCGACGCGCTTCAAGCAACTCGTGGTGCAGTCGTTGCCAAATGGTGGGAAAGCAGAACAGTCCGACCTCAGTACACCCGTACGCTCCTCTGGACCTCGTCGGGAAAGGAGCAGCGTCTCACGTCACCCGGTCGGCTGATACTCCTGTGTGTCGGCAACAACACTGATGTGGTCAACGTGTCGGTGCTGTGTCGCTGGAGTGTTCGACTGAGCGTTCCATCTCTTGAGACACCTGAAGAGACCACCGCTCCCATCATGACACAAGGTTCCCTGTACAACGATTCCCTTTCCACAAATGACTTCAAGTCCATCCTCCTAGGATCCACACCACTGGATATTGCCCCTGATGGAGCAGTCTTCCAGCTGGACCGTCCGCTGTCCATTGACTACAGCCTTGGAACTGGAGATGTTGACCGTGCTGTTTATTGGCACCTCAAGAAGTTTGCTGGAAATGCTGGCACACCTGCAGGCTGGTTTCGCTGGGGCATCTGGGACAACTTCAATAAGACGTTCACAGATGGCGTTGCCTACTACTCTGATGAGCAGCCCCGTCAAATCCTGCTGCCTGTTGGCACTGTCTGCACCAGGGTTGACTCGGAAAACTAA

>China(N).....Sole..................2012....Fish....KJ541748

ATGGTACGCAAAGGTGAGAAGAAATTGGCAAAACCCGCGACCACCAAGGCCGCGAATCCGCAACCCCGCCGACGTGCTAACAATCGTCGGCGTAGTAATCGCACTGACGCACCTGTGTCTAAGGCCTCGACTGTGACTGGATTTGGACGTGGGACCAATGACGTCCATCTCTCAGGTATGTCGAGAATCTCCCAGGCCGTCCTCCCAGCCGGGACAGGAACTGACGGATACGTCGTTGTTGACGCAACCATCGTCCCCGACCTCCTGCCACGACTGGGACACGCTGCTAGAATCTTCCAGCGATACGCTGTTGAAACATTGGAGTTTGAAATTCAGCCAATGTGCCCCGCAAACACGGGCGGTGGTTACGTTGCTGGCTTCCTGCCTGATCCAACTGACAACGACCACACCTTCGACGCGCTTCAAGCAACTCGTGGTGCAGTCGTTGCCAAATGGTGGGAAAGCAGAACAGTCCGACCTCAGTACACCCGTACGCTCCTCTGGACCTCGTCGGGAAAGGAGCAGCGTCTCACGTCACCTGGTCGGCTGATACTCCTGTGTGTCGGCAACAACACTGATGTGGTCAACGTGTCGGTGCTGTGTCGCTGGAGTGTTCGACTGAGCGTTCCATCTCTTGAGACACCTGAAGAGACCACCGCTCCCATCATGACACAAGGTTCCCTGTACAACGATTCCCTTTCCACAAATGACTTCAAGTCCATCCTCCTAGGATCCACACCACTGGATATTGCCCCTGATGGAGCAGTCTTCCAGCTGGACCGTCCGCTGTCCATTGACTACAGCCTTGGAACTGGAGATGTTGATCGTGCTGTTTATTGGCACCTCAAGAAGTTTGCTGGAAATGCTGGCACACCTGCAGGCTGGTTTCGCTGGGGCATCTGGGACAACTTTAATAAGACGTTCACAGATGGCGTTGCCTACTACTCTGATGAGCAGCCCCGTCAAATCCTGCTGCCTGTTGGCACTGTCTGCACCAGGGTTGACTCGGAAAACTAA

>Korea........Bream.................2005....Unknown.DQ116035

ATGGTACGCAAGGGTGAGAAGAAATTGGCAAAACCCGCGACCACCAAGGCCGCGAATCCGCAACCCCGCCGACGTGCTAACAATCGTCGGCGTAGTAATCGCACTGACGCACCTGTGTCTAAGGCCTCGACTGTAACTGGATTTGGACGTGGGACCAATGACGTCCATCTCTCAGGTATGTCGAGAATCTCCCAGGCCGTCCTCCCAGCCGGGACAGGAACAGACGGATACGTTGTTGTTGACGCAACCATCGTCCCCGACCTCCTGCCACGACTGGGACACGCTGCTAGAATCTTCCAGCGATACGCTGTTGAAACACTGGAGTTTGAAATTCAGCCAATGTGCCCCGCAAACACGGGCGGTGGTTACGTTGCTGGCTTCCTGCCTGATCCAACTGACAACGATCACACCTTCGACGCGCTTCAAGCAACTCGTGGTGCAGTCGTTGCCAAATGGTGGGAAAGCAGAACAGTCCGACCTCAGTACACCCGCACGCTCCTCTGGACCTCGTCGGGAAAGGAGCAGCGTCTCACGTCACCTGGTCGGCTGATACTCCTGTGTGTCGGCAACAACACTGATGTGGTCAACGTGTCAGTGCTGTGTCGCTGGAGTGTTCGACTGAGCGTTCCATCTCTTGAGACACCTGAAGAGACCACCGCTCCCATCATGACACAAGGTTCCCTGTACAACGATTCCCTTTCAACAAATGACTTCAAGTCCATCCTCCTAGGATCCACACCACTGGACATTGCCCCTGATGGAGCAGTCTTCCAGCTGGACCGTCCGCTGTCCATTGACTACAGCCTTGGAACTGGAGATGTTGACCGTGCTGTTTACTGGCACCTCAAGAAGTTTGCTGGAAATGCTGGCACACCTGCAGGCTGGTTTCGCTGGGGCATCTGGGACAACTTTAACAAGACGTTCGCAGATGGCGTTGCCTACTACTCTGATGAGCAGCCTCGTCAAATCCTGCTGCCTGTTGGCACTGTCTGCACCAGGGTCGACTCGGAAAACTAA

>Korea........Grouper (Sevenband)...2005....Unknown.DQ116036

ATGGTACGCAAGGGTGAGAAGAAATTGGCAAAACCCGCGACCACCAAGGCCGCGAATCCGCAACCCCGCCGACGTGCTAACAATCGTCGGCGTAGTAATCGCACTGACGCACCTGTGTCTAAGGCCTCGACTGTAACTGGATTTGGACGTGGGACCAATGACGTCCATCTCTCAGGTATGTCGAGAATCTCCCAGGCCGTCCTCCCAGCCGGGACAGGAACAGACGGATACGTTGTTGTTGACGCAACCATCGTCCCCGACCTCCTGCCACGACTGGGACACGCTGCTAGAATCTTCCAGCGATACGCTGTTGAAACACTGGAGTTTGAAATTCAGCCAATGTGCCCCGCAAACACGGGCGGTGGTTACGTTGCTGGCTTCCTGCCTGATCCAACTGACAACGATCACACCTTCGACGCGCTTCAAGCAACTCGTGGTGCAGTCGTTGCCAAATGGTGGGAAAGCAGAACAGTCCGACCTCAGTACACCCGCACGCTCCTCTGGACCTCGTCGGGAAAGGAGCAGCGTCTCACGTCACCTGGTCGGCTGATACTCCTGTGTGTCGGCAACAACACTGACGTGGTCAACGTGTCGGTGCTGTGTCGCTGGAGTGTTCGACTGAGCGTTCCATCTCTTGAGACACCTGAGGAGACCACCGCTCCCATCATGACACAAGGTTCCCTGTACAACGATTCCCTTTCCACAAATGACTTCAAGTCCATCCTCCTAGGATCCACACCACTGGACATTGCCCCTGATGGAGCAGTTTTCCAGCTGGACCGTCCGCTGTCCATTGACTACAGCCTTGGAACTGGAGATGTTGACCGTGCTGTTTATTGGCACATCAAGAAGTTTGCTGGAAATGCTGGCACACCTGCAGGCTGGTTTCGCTGGGGCATCTGGGACAACTTTAACAAGACGTTCACAGATGGCGTTGCTTACTACTCTGATGAGCAGCCTCGTCAAATCCTGCTGCCTGTTGGCACTGTCTGCACCAGGGTTGACTCGGAAAACTAA

>Korea........Flounder(Japanese)....2006....Unknown.DQ116037

ATGGTACGCAAGGGTGAGAAGAAATTGGCAAAACCCGCGACCACCAAGGCCGCGAATCCACAAACCCGCCGACGTGCTAACAATCGTCGGCGTAGTAATCGCACTGACGCACCTGTGTCTAAGGCCTCGACTGTAACTGGATTTGGACGTGGGACCAATGACGTCCATCTCTCAGGTATGTCGAGAATCTCCCAGGCCGTCCTCCCAGCCGGGACAGGAACAGACGGATACGTTGTTGTTGACGCAACCATCGTCCCCGACCTCCTGCCACGACTGGGACACGCTGCTAGAATCTTCCAGCGATACGCTGTTGAAACACTGGAGTTTGAAATTCAGCCAATGTGCCCCGCAAACACGGGCGGCGGTTACGTTGCTGGCTTCCTGCCTGATCCAACTGACAACGATCACACCTTCGACGCGCTTCAAGCAACTCGTGGTGCAGTCGTTGCCAAATGGTGGGAAAGCAGAACAGTCCGACCTCAGTACACCCGCACGCTCCTCTGGACCTCGTCGGGAAAGGAGCAGCGTCTCACGTCACCTGGTCGGCTGATACTCCTGTGTGTCGGCAACAACACTGATGTGGTCAACGTGTCGGTGCTGTGTCGCTGGAGTGTTCGACTGAGCGTTCCATCTCTTGAGACACCTGAAGAGACCACCGCTCCCATCATGACACAAGGTTCCCTGTACAACGATTCCCTTTCCACAAATGACTTCAAGTCCATCCTCCTAGGATCCACACCACTGGACATTGCCCCTGATGGAGCAGTCTTCCAGCTGGACCGTCCGCTGTCCATTGACTACAGCCTTGGAACTGGAGATGTTGACCGTGCTGTGTATTGGCACCTCAAGAAGTTTGCTGGAAATGCTGGCACACCTGCAGGCTGGTTTCGCTGGGGCATCTGGGACAACTTCAACAAGACGTTCGCAGATGGCGTTGCCTACTACTCTGATGAGCAGCCTCGTCAAATCCTGCTGCCTGTTGGCACTGTCTGCACCAGGGTTGACTCGGAAAACTAA

>Korea........Mullet................2005....Unknown.DQ116038

ATGGTACGCAAGGGTGAGAAGAAATTGGCAAAACCCGCGACCACCAAGGCCGCGAATCCGCAACCCCGCCGACGTGCTAACAATCGTCGGCGTAGTAATCGCACTGACGCACCTGTGTCTAAGGCCTCGACTGTAACTGGATTTGGGCGTGGGACCAATGACGTCCATCTCTCAGGTATGTCGAGAATCTCCCAGGCCGTCCTCCCAGCCGGGACAGGAACAGACGGATACGTTGTTGTTGACGCAACCATCGTCCCCGACCTCCTGCCACGACTGGGACACGCTGCTAGAATCTTCCAGCGATACGCTGTTGAAACACTGGAGTTTGAAATTCAGCCAATGTGCCCCGCAAACACGGGCGGTGGTTACGTTGCTGGCTTCCTGCCTGATCCAACTGACAACGATCACACCTTCGACGCGCTTCAAGCAACTCGTGGTGCAGTCGTTGCCAAATGGTGGGAAAGCAGAACAGTCCGACCTCAGTACACCCGCACACTCCTCTGGACCTCGTCGGGAAAGGAGCAGCGTCTCACGTCACCTGGTCGGCTGATACTCCTGTGTGTCGGCAACAACACTGATGTGGTCAACGTGTCGGTGCTGTGTCGCTGGAGTGTTCGACTGAGCGTTCCATCTCTTGAGACACCTGAGGAGACTACCGCTCCCATCATGACACAAGGTTCCCTGTACAACGATTCCCTTTCCACAAATGACTTCAAGTCCATCCTCCTAGGATCCACACCACTGGACATTGCCCCTGATGGAGCAGTCTTCCAGCTGGACCGTCCGCTGTCCATTGACTACAGCCTTGGAACTGGAGATGTGGACCGTGCTGTTTATTGGCACCTCAAGAAGTTTGCTGGAAATGCTGGCACACCTGCAGGCTGGTTTCGCTGGGGCATCTGGGACAACTTCAACAAGACGTTCACAGATGGCGTTGCTTACTACTCTGATGAGCAGCCTCGTCAAATCCTGCTGCCTGTTGGCACTGTCTGCACCAGGGTTGACTCGGAAAACTAA

>Korea........Flounder(Japanese)....2006....Unknown.DQ864760

ATGGTACGCAAGGGTGAGAAGAAATTGGCAAAACCCGCGACCACCAAGGCCGCGAATCCGCAACCCCGCCGACGTGCTAACAATCGTCGGCGTAGTAATCGCACTGACGCACCTGTGTCTAAGGCCTCGACTGTAACTGGATTTGGGCGTGGGACCAATGACGTCCATCTCTCAGGTATGTCGAGAATCTCCCAGGCCGTCCTCCCAGCCGGGACAGGAACGGACGGATACATTGTTGTTGACGCAACCATCGTCCCCGACCTCCTGCCACGACTGGGACACGCTGCTAGAATCTTCCAGCGATACGCTGTTGAAACACTGGAGTTTGAAATCCAGCCAATGTGCCCCGCAAACACGGGCGGTGGTTACGTTGCTGGCTTCCTGCCTGATCCAACTGACAACGATCACACCTTCGACGCGCTTCAAGCAACTCGTGGTGCAGTCGTTGCCAAATGGTGGGAAAGCAGAACAGTCCGACCTCAGTACACCCGCACACTCCTCTGGACCTCGTCGGGAAAGGAGCAGCGTCTCACGTCACCTGGTCGGCTGATACTCCTGTGTGTCGGCAACAACACTGATGTGGTCAACGTGTCGGTGCTGTGTCGCTGGAGTGTTCGACTGAGCGTTCCATCTCTTGAGACACCTGAGGAGACTACCGCTCCCATCATGACACAAGGTTCCCTGTACAACGATTCCCTTTCCACAAATGACTTCAAGTCCATCCTCCTAGGATCCACACCACTGGACATTGCCCCTGATGGAGCAGTCTTCCAGCTGGACCGTCCGCTGTCCATTGACTACAGCCTTGGAACTGGAGATGTGGACCGTGCTGTTTATTGGCACCTCAAGAAGTTTGCTGGAAATGCTGGCACACCTGCAGGCTGGTTTCGCTGGGGCATCTGGGACAACTTCAATAAGACGTTCACAGATGGCGTTGCTTACTACTCTGATGAGCAGCCTCATCAAATCCTGCTGCCTGTTGGCACTGTCTGTACCAGGGTTGACTCGGAAAACTAA

>Korea........Grouper (Sevenband)...2008....Cell....KM095959

ATGGTACGCAAAGGTGAGAAGAAATTGGCAAAACCCGCGACCACCAAGGCCGCGAATCCGCAACCCCGCCGACGTGCTAACAATCGTCGGCGTAGTAATCGCACTGACGCACCTGTGTCTAAGGCCTCGACTGTGACTGGATTTGGACGTGGGACCAATGACGTCCATCTCTCAGGTATGTCGAGAATCTCCCAGGCCGTCCTCCCAGCCGGGACAGGAACTGACGGATACGTCGTTGTTGACGCAACCATCGTCCCCGACCTCCTGCCACGACTGGGACACGCTGCTAGAATCTTCCAGCGATACGCTGTTGAAACACTGGAGTTTGAAATTCAGCCAATGTGCCCCGCAAACACGGGCGGTGGTTACGTTGCTGGCTTCCTGCCTGATCCAACTGACAACGACCACACCTTCGACGCGCTTCAAGCAACTCGTGGTGCAGTCGTTGCCAAATGGTGGGAAAGCAGAACAGTCCGACCTCAGTACACCCGTACGCTCCTCTGGACCTCGTCGGGAAAGGAGCAGCGTCTCACGTCACCTGGTCGGCTGATACTCCTGTGTGTCGGCAACAACACTGATGTGGTCAACGTGTCGGTGCTGTGTCGCTGGAGTGTTCGACTGAGCGTTCCATCTCTTGAGACACCTGAAGAGACCACCGCTCCCATCATGACGCAAGGTTCCCTGTACAACGATTCCCTTTCCACAAATGACTTCAAGTCCATCCTCCTAGGATCCACACCACTAGATATTGCCCCTGATGGAGCAGTCTTCCAGCTGGACCGTCCGCTGTCCATTGACTACAGCCTTGGAACTGGAGATGTTGACCGTGCTGTTTATTGGCACCTCAAGAAGTTTGCTGGAAATGCTGGCACACCTGCAGGCTGGTTTCGCTGGGGCATCTGGGACAACTTCAATAAGACGTTCACAGATGGCGTTGCCTACTACTCTGATGAGCAGCCCCGTCAAATCCTGCTGCCTGTTGGCACTGTCTGCACCAGGGTTGACTCGGAAAACTAA

>Japan........Grouper (Sevenband)...2001....Cell....AB373029

ATGGTACGCAAAGGTGAGAAGAAATTGGCAAAACCCGCGACCACCAAGGCCGCGAATCCGCAACCCCGCCGACGTGCTAACAATCGTCGGCGTAGTAATCGCACTGACGCACCTGTGTCTAAGGCCTCGACTGTAACTGGATTTGGACGTGGGACCAATGACGTCCATCTTTCAGGTATGTCGAGAATCTCCCAGGCCGTCCTCCCAGCCGGGACAGGAACAGACGGATACGTTGTTGTTGATGCAACCATCGTCCCCGACCTCCTGCCACGACTGGGACACGCTGCTAGAATCTTCCAGCGATACGCTGTTGAAACACTGGAGTTTGAAATTCAGCCAATGTGCCCCGCAAACACGGGCGGTGGTTACGTTGCTGGCTTCCTGCCTGATCCAACTGACAACGATCACACCTTCGACGCGCTTCAAGCAACTCGTGGTGCAGTCGTTGCCAAATGGTGGGAAAGCAGAACAGTCCGACCTCAGTACACCCGCACGCTCCTCTGGACCTCGTCGGGAAAGGAGCAGCGTCTCACGTCACCTGGTCGGCTGATACTCCTGTGTGTCGGCAACAACACTGATGTGGTCAACGTGTCAGTACTGTGTCGCTGGAGTGTTCGACTGAGCGTTCCATCTCTTGAGACACCTGAAGAGACCACCGCTCCCATCATGACACAAGGTTCCCTGTACAACGATTCCCTTTCCACAAATGACTTCAAGTCCATCCTCCTAGGATCCACACCACTGGACATTGCCCCTGATGGAGCAGTCTTCCAGCTGGACCGTCCGCTGTCCATTGACTACAGCCTTGGAACTGGAGATGTTGACCGTGCTGTTTACTGGCACCTCAAGAAGTTTGCTGGAAATGCTGGCACACCTGCAGGCTGGTTTCGCTGGGGCATCTGGGACAACTTCAACAAGACGTTCACAGATGGCGTTGCCTACTACTCTGATGAGCAGCCCCGTCAAATCCTGCTGCCTGTTGGCACTGTCTGCACCAGGGTTGACTCGGAAAACTAA

>Japan........Grouper (Sevenband)...2004....Cell....AY324870

ATGGTACGCAAAGGTGAGAAGAAATTGGCAAAACCCGCGACCACCAAGGCCGCGAATCCGCAACCCCGCCGACGTGCTAACAATCGTCGGCGTAGTAATCGCACTGACGCACCTGTGTCTAAGGCCTCGACTGTAACTGGATTTGGACGTGGGACCAATGACGTCCATCTCTCAGGTATGTCGAGAATCTCCCAGGCCGTCCTCCCAGCCGGGACAGGAACAGACGGATACGTTGTTGTTGACGCAACCATCGTCCCCGACCTCCTGCCACGACTGGGACACGCTGCTAGAATCTTCCAGCGATACGCTGTTGAAACACTGGAGTTTGAAATTCAGCCAATGTGCCCCGCAAACACGGGCGGTGGTTACGTTGCTGGCTTCCTGCCTGATCCAACTGACAACGATCACACCTTCGACGCGCTTCAAGCAACTCGTGGTGCAGTCGTTGCCAAATGGTGGGAAAGCAGAACAGTCCGACCTCAGTACACCCGCACGCTCCTCTGGACCTCGTCGGGAAAGGAGCAGCGTCTCACGTCACCTGGTCGGCTGATACTCCTGTGTGTCGGCAACAACACTGATGTGGTCAACGTGTCAGTGCTGTGTCGCTGGAGTGTTCGACTGAGCGTTCCATCTCTTGAGACACCTGAAGAGACCACCGCTCCCATCATGACACAAGGTTCCCTGTACAACGATTCCCTTTCCACAAATGACTTCAAGTCCATCCTCCTAGGATCCACACCACTGGACATTGCCCCTGATGGAGCAGTCTTCCAGCTGGACCGTCCGCTGTCCATTGACTACAGCCTTGGAACTGGAGATGTTGACCGTGCTGTTTATTGGCACCTCAAGAAGTTTGCTGGAAATGCTGGCACACCTGCAGGCTGGTTTCGCTGGGGCATCTGGGACAACTTCAACAAGACGTTCGCAGATGGCGTTGCCTACTACTCTGATGAGCAGCCTCGTCAAATCCTGCTGCCTGTTGGCACTGTCTGCACTAGGGTTGACTCGGAAAACTAA

# Supplementary Figure S3. Similarity matrix for cds RNA 2 NNV DNA

# Supplementary Table S4. Polymorphic nucleotides from RNA 2 NNV sequences for 82 samples.

>India........Barramundi............2006....Fish....FR669249

GAGATTGCGAACAGTGACCCTTTGTTCCATTTAGTAATATACCCTATCCGCGAGTTCCCTACTTAGGTTCCTCTCCGCTATATGAAGTCCTCCCGTTGGT

CGATAGCCATGAGTTACTATCTGACTCTATGTTCAATCGACTAACACACGCTCAGGCCAGCCTGCCTCGTGATATCTTTTTCCATAATGCTGGGACCCGC

GGTCCACCTATGTCGCTGCTGTGTCCCTTA

>India........Barramundi............2006....Unknown.HM485328

GAGATTGCGAACAGTGACCCTTTGTTCCATTTAGTAATATACCCTATCCGCGAGTTCCCTACTTAGGTTCCTCTCCGCTATATGAAGTCCTCCCGTTGGT

CGATAGCCATGAGTTACTATCTGACTCTATGTTCAATCGACTAACACACGCTCAGGCCAGCCTGCCTCGTGATATCTTTTTCCATAATGCTGGGACCCGC

GGTCCACCTATGTCGCTGCTGTGTCCCTTA

>India........Barramundi............2008....Unknown.GU826692

GAGATTGCGAACAGTGACCCTTTGTTCCATTTAGTAATATACCCTATCCGCGAGTTCCCTACTTAGGTTCCTCTCCGCTATATGAAGTCCTCTCGTTGGT

CGATAGCCATGAGTTACTATCTGACTCTATGTTCAATCGACTAACACACGCTCAGGCCAGCCTGCCTCGTGATATCTTTTTCCATAATGCTGGGACCCGC

GGTCCACCTATGTCGCTGCTGTGTCCCTTA

>India........Barramundi............2008....Unknown.GU826693

GAGATTGTGAACAGTGACCCTTTGTTCCATTTAGTAATATGCCCTATCCGCGAGTTCCCTACTTAGGTTCCTCTCCGCTATATGAAGTCCTCCCGTTGGT

CGATAGCCATGAGTTACTACCTGACTCTATGTTCAATCGACTAACACACGCTCAGGCCAGCCTGCCTCGTGATATCTTTTTCCATAATGCTGGGACCCGC

GGTCCACCTATGTCGCTGCTGTGTCCCTTA

>India........Barramundi............2009....Unknown.GU953669

GAGATTGCGAACAGTGACCCTTTGTTCCATTAAGTGATATACCCTATCCGCGAGTTCCCTACTTAGATTTCTCCCTGCTATATGAAGTCCTCCCGTTGGT

CGATAGCCATGAATTACTATCTGGATCTATGTTCAATCGACTAACACACGCTCAGGCCAGCCTGCCTCGTGATATTATCTTCCATAATGCCGGGACCCGC

AGTCCACCTACGTCGCTGCTGTGTCCCCTA

>India........Barramundi............2010....Unknown.GU592791

G-GATTGCGAACAGTGACCCTTTGTTCCATTTAGTAATATACCCTATCCGCGAGTTCCCTACTTAGGTTCCTCTCCGCTATATGAAGTCCTCCCGTTGGT

CGATAGCCATGAGTTACTATCTGACTCTATGTTCAATCGACTAACACACGCTCAGGCCAGCCTGCCTCGTGATATCTTTTTCCATAATGCTGGG-CCCGC

GCTGCACCTCTGTCGCTGTGTGCTCCCTTA

>India........Barramundi............2013....Fish(W).JF412257

GAGATTGCGAACAGTGACCCTTTATTCCATTAAGTGATATACCCTATCCGCGAGTTCCCTACTTAGGTTCCTCCCTGCTATATGAAGTCCTCCCGTTGGT

CGATAGCCATGAATTACTATCTGGATCTATGTTCAATCGACTAACACACGCTCAGGCCAGCCTGCCTCGTGATATTATCTTCCATAATGCTGGGACCCGC

AGTCCACCTACGTCGCTGCTGTGTCCCCTA

>India........Barramundi............2013....Fish(Y).JF412258

GAGATTGCGAACAGTGACCCTTTGTTCCATTAAGTGATATACCCTATCCGCGAGTTCCCTACTTAGATTTCTCCCTGCTATATGAAGTCCTCCCGTTGGT

CGATAGCCATGAATTACTATCTGGATCTATGTTCAATCGACTAACACACGCTCAGGCCAGCCTGCCTCGTGATATTATCTGCCATAATGCCGGCACCCGC

AGTCCACCTACGTCGCTGCTGTGTCCCCTA

>India........Barramundi............2013....Fish(W).JF412259

GAGATTGCGAGCAGTGACCCTTTGTTCCATTAAGTGATATACCCTATCCGAGAGTTCCCTACTTAGGTTCCTCCCTGCTATATGAAGTCCTCCCGTTGGT

CGATAGCCATGAATTACTATCTGGATCTATGTTCAATCGACTAACACACGCTCAGGCCAGCCTGCCTCGTGATATTATCTGCCATAATGCTGGCACCCGC

AGTCCACCTACGTCGCTGCTGTGTCCCCTA

>India........Barramundi............2013....Fish....JF412260

GAGATTGCGAACGGTGACCCTTTGTTCCATTAAGTGATATACCCTATCCGCGAGTTCCCTACTTAGGTTCCTCCCTGCTATATGAAGTCCTCCCGTTGGT

CGATAGCCATGAATTACTATCTGGATCTATGTTCAATCGACTAACACACGCTCAGGCCAGCCTGCCTCGTGATATTATCTTCCATAATGCTGGGACCCGC

AGTCCACCTACGTCGCTGCTGTGTCCCCTA

>India........Barramundi............2013....Fish(W).JF412261

GAGATTGCGAACAGTGACCCTTTGTTCCATTAAGTGATATACCCTATCAGCGAGTTCCCTACTTAGGTTCCTCCCTGCTATATGAAGTCCTCCCGCTGGT

CGATAGCCATGAATTACTATCTGGATCTATGTTCAATCGACTAACACACGCCCAGGCCAGCCTGCCTCGTGATATTATCTTCCATAATGCTGGGACCCGC

AGTCCACCTACGTCGCTGCTGTGTCCCCTA

>India........Barramundi............2013....Fish....JF412262

GAGATTGCGAACAGTGACCCTTTGTTTCATTAAGTGATATACCCTATCCACGAGTTCCCTACTTAGGTTCCCCCCTGCTATATGAAGTCCTCCCGTTGGT

CGATAGCCATGAATTACTATCTGGATCTATGTTCAATCGACTAACACACGCTCAGGCCAGCCTGCCTCGTGATATTATCTGCCATAATGCTGGGACCCGC

AGTCCACCTACGTCGCTGCTGTGTCCCCTA

>India........Barramundi............2013....Fish....JF412263

GAGATTGCGAACAGTGACCCATTGTTCCATTAGGTGATATACCCTACCCGCGAGTTCCCCACTCAGGTTCCTCCCTGCTATATGAAGTCCTCCCGTTGGT

CGATAGCCATGAATTACTATCTGGATCTATGTTCAATCGACTAACACACGCTCAGGCCAGCCTGCCTCGTGATATTATCTGCCATAATGCTGGGACCCGC

AGTCCACCTACGTCGCTGCTGTGTCCCCTA

>India........Barramundi............2013....Fish....JF412264

GAGATTGCGAACAGTGACCCTTTGTTCCATTAAGTGATATACCCTATCCGCGAGTTCCCTACTTAGGTTCCTCCCTGCGATATTAAGTCCTCCCGTTGGT

CGATAGCCATGAATTACTATCTGGATCTATGTTCAATCGACTAACACACGCTCAGGCCAGCCTGCCTCGTGATATTATCTGCCATAATGCTGGGACCCGC

AGTCCACCTACGTCGCTGCTGTGTCCCCTA

>India........Barramundi............2013....Fish(W).JF412265

GAGATTGCGAACAGTGACCCTTTGTTCCATTAAGTGATATACCCTATCCGCGAGTTCCCTACTTGGGTTCCTCCCTGCTATATGAAGTCCTCCCGTTGGT

CGATAGCCATGAATTACTATCTGGATCTATGTTCAATCGACTAACACACGCTCAGGCCAGCCTGCCTCGTGATATTATCTTCCATAATGCTGGGACCCGC

AGTCCACCTACGTCGCTGCTGTGTCCCCTA

>India........Barramundi............2013....Fish....JF412267

GAGATTGCGAGCAGTGACCCTTTGTTCCATTAAGTGATATACCCTATCCGAGAGTTCCCTACTTAGGTTCCTCCCTGCTATATGAAGTCGTCCCGTTAAT

CGATAGCCATGAATTACTATCTGGATCTATGTTCAATCGACTAACACACGCTCAGGCCTGCCTGCCTCGTGATATTATCTTCCATAATGCTGGGACCCGC

AGTCCACCTACGTCGCTGCTGTGTCCCCTA

>India........Barramundi............2013....Fish(W).JF412268

GAGATTGCGAACGGTGACCCTTTGTTCCATTAAGTGATATACCCTATCCGCGAGTTCCCTACTTAGGTTCCTCCCTGCTATATGAAGTCCTCCCGTTGGT

CGATAGCCATGAATTACTATCTGGATCTATGTTCAATCGACTAACACACGCTCAGGCCAGCCTGCCTCGTGATATTGTCTTCCGTAATGCTGGGACCCGC

AGTCCACCTACGTCGCTGCTGTGTCCCCTA

>India........Barramundi............2013....Fish(W).JF412269

GAGGTTGCGAACAGTCACCCTTTGTTCCATTAAGTGATATACCCTATCCGCGAGTTCCCTACTTAGGTTCCTCCCTGCTATATGAAGTCCTCCCGTTGGT

CGATAGCCATGAATTACTATCTGGATCTATGTTCAATCGACTAACACACGCTCAGGCCAGCCTGCCTCGTGATATTATCTTCCATAATGCTGGGACCCGC

AGTCCACCTACGTCGCTGCTGTGTCCCCTA

>India........Barramundi............2013....Fish(W).JF412270

AAGATTGCGAACAGTGACCCTTTGTTCCATTAAGTGATATACCCTATCCGCGAGTTCCCTACTTAGGTTCCTCCCTGCTATATGAAGTCCTCCCGTTGGT

CGATTGCCATGAATTACTATCTGGATCTATGTTCTATCGACCAACACACGCTCAGGCCAGCCTGCCTCGTGATATTATCTTCCATAATGCTGGGACCCGC

AGTCCACCTACGTCGCTGCTGTGTCCCCTA

>India........Barramundi............2013....Fish....JF412271

GAGAAAGCGAACAGTGACCCTTTGTTCCATTAAGTGATATACCCTATCCGCGAGTTCCCTACTTAGGTTCCTCCCTGCTATATGAAGTCCTCCCGTTGGT

CGATAGCCATGAATTACTATCTGGATCTATGTTCAATCGACTAACACACGCTCAGGCCAGCCTGCCTCGTGATATTATCTTCCATAATGCTGGGACCCGC

AGTCCACCTACGTCGCTGCTGTGTCCCCTA

>India........Barramundi............2013....Fish(W).JF412272

GAGATTGCGAACAGTGACCCTTTGTTCCACTAAGTGATATACCCTATCCGCGAGTTCCCTACTTAGGTTCCTCCCTGCTATATGAAGTCCTCCCGTTGGT

CGATAGCCATGAATTACTATCTGGATCTATGTTCAATCGACTAACACACGCTCAGGCCAGCCTGCCTCGTGATATTATCTTCCATAATGCTGGGACCCGC

AGTCCTCCTACGTCGCTGCTGTGTCCCCTA

>India........Barramundi............2013....Fish(W).JF412273

GAGAATACGAACAGTGATCCTTTGTTCCATTAAGTGATATACCCTATCCGCGAGTTCCCTACTTAGGTTCCTCCTTGCTATATGAAGTCCTCCCGTTGGT

CGATAGCCATGAATAACTATCTGGATCTATGTTCAATCGACTAACACACGCTCAGGCCAGCCTGCCTCGTGATATTATCTTCCATAATGCTGGGACCCGC

AGTCCACCTACGTCGCTGCTGTGTCCCCTA

>Australia(N).Barramundi............2007....Cell....GQ402011

GAGATTGCGAACAGTGACCCTTTGTTCCATTTAGTAATATACCCTATCCGCGAGTTCCCTACTTAGGTTCCTCCCCGCTATATGAAGTCCTCCTGTTGGT

CGATAGCTATTAGTTACTATCTGACTCTACGTTCAATCGACTAACGCACGCTCAGGCCAGCCTGCCTCGTTATATCTTTCTCCATAATGCTGGGACCCGC

AGTCCACCTACGTCGCTGCTGTGTCCCCTG

>Australia(E).Bass..................2006....Cell....GQ402013

GGGATTGCGAACAACAACGTTCCGTTCTGTTTAGTGATACATCCTATCCGCGTGCTTTCTACCTAGGTTCCTCCCCACTGGGTGAAGTCCACCCGTAGGC

CAATAGCCACGATTTGCTGTCTAAGTCTACTCGCCGCCGACTGGCGAGTGCTTGGACTAATCAATCTTGAGATTTCTCCCTCTACCCATCTGCGACCCAC

AGGCTACCCCTGTTCCTGCCGTGTCCCCTA

>Indonesia....Grouper (Tiger).......2008(J).Fish....HQ859927

GAGATTGCGAACAGTGACCCTTTGTCCCATTTAGTGATATACCCTATCCGCGAGTTCCCTACTTAGGTTCCTCTCCGCTATATGAAGTCCTCCCGTTGGT

CGATAGCCACGGGTTACTATCTGACTCTATGTTCAATCGACTAACACACGTTCAGACCAGCCTGCTTCGTGATATCTTTTTCCATAATGCTGGGACCCGC

AGTCTATCTACGTTGCTGCTGTGTCCCCTA

>Indonesia....Grouper (Tiger).......2008(J).Fish....HQ859928

GAGATTGCGAACAGTGACCCTTTGTCCCATTTAGTGATATACCCTATCCGCGAGTTCCCTACTTAGGTTCCTCTCCGCTATATGAAGTCCTCCCGTTGGT

CGATAGCCACGGGTTACTATCTGACTCTATGTTCAATCGACTAACACACGTTCAGACCAGCCTGCTTCGTGATATCTTTTTCCATAATGCTGGGACCCGC

AGTCTATCTACGTTGCTGCTGTGTCCCCTA

>Indonesia....Grouper (Tiger).......2008(J).Fish....HQ859929

GAGATTGCGAACAGTGACCCTTTGTCCCATTTAGTGATATACCCTATCCGCGAGTTCCCTACTTAGGTTCCTCTCCGCTATATGAAGTCCTCCCGTTGGT

CGATAGCCACGGGTTACTATCTGACTCTATGTTCAATCGACTAACACACGTTCAGACCAGCCTGCTTCGTGATATCTTTTTCCATAATGCTGGGACCCGC

AGTCTATCTACGTTGCTGCTGTGTCCCCTA

>Indonesia....Grouper (Humpback)....2008(J).Fish....HQ859924

GAGATTGCGAACAGTGACCCTTTGTCCCATTTAGTGATATACCCTATCCGCGAGTTCCCTACTTAGGTTCCTCTCCGCTATATGAAGTCCTCCCGTTGGT

CGATAGCCACGGGTTACTATCTGACTCTATGTTCAATCGACTAACACGCGCTCAGGCCAGCCTGCCTCGTGATATCTTTCTCCATAATGCTGGGACCCGC

AGTCTACCTATGTCGCTGCTGTGTCCCCTA

>Indonesia....Grouper (Humpback)....2008(J).Fish....HQ859925

GAGATTGCGAACAGTGACCCTTTGTCCCATTTAGTGATATACCCTATCCGCGAGTTCCCTACTTAGGTTCCTCTCCGCTATATGAAGTCCTCCCGTTGGT

CGATAGCCACGGGTTACTATCTGACTCTATGTTCAATCGACTAACACGCGCTCAGGCCAGCCTGCCTCGTGATATCTTTCTCCATAATGCTGGGACCCGC

AGTCTACCTATGTCGCTGCTGTGTCCCCTA

>Indonesia....Grouper (Humpback)....2008(J).Fish....HQ859926

GAGATTGCGAACAGTGACCCTTTGTCCCATTTAGTGATATACCCTATCCGCGAGTTCCCTACTTAGGTTCCTCTCCGCTATATGAAGTCCTCCCGTTGGT

CGATAGCCACGGGTTACTATCTGACTCTATGTTCAATCGACTAACACGCGCTCAGGCCAGCCTGCCTCGTGATATCTTTCTCCATAATGCTGGGACCCGC

AGTCTACCTATGTCGCTGCTGTGTCCCCTA

>Malaysia 2...Grouper (Tiger).......2009(J).Fish....HQ859940

GATATTGCGAACAGTGACCCTTTGTCCCATTAAGTGATATACCCTATCCGCGAGTTCCCTACTTAGGTTCCTCCCCGCTATATGAAGTCCTCCCGTTGGT

CGATAGCCATGAATTACTATCTGGATCTATGTTCAATCGACTAACACACGCTCAGGCCAGCCTGCCTCGTGATATTATCTTCCATAATGCTGGGACCCGC

AGTCCACCTACGTCGCTGCTGTGTCCCCTA

>Malaysia 2...Grouper (Tiger).......2009(J).Fish....HQ859941

GATATTGCGAACAGTGACCCTTTGTCCCATTAAGTGATATACCCTATCCGCGAGTTCCCTACTTAGGTTCCTCCCCGCTATATGAAGTCCTCCCGTTGGT

CGATAGCCATGAATTACTATCTGGATCTATGTTCAATCGACTAACACACGCTCAGGCCAGCCTGCCTCGTGATATTATCTTCCATAATGCTGGGACCCGC

AGTCCACCTACGTCGCTGCTGTGTCCCCTA

>Malaysia 2...Grouper (Tiger).......2009(J).Fish....HQ859942

GATATTGCGAACAGTGACCCTTTGTCCCATTAAGTGATATACCCTATCCGCGAGTTCCCTACTTAGGTTCCTCCCCGCTATATGAAGTCCTCCCGTTGGT

CGATAGCCATGAATTACTATCTGGATCTATGTTCAATCGACTAACACACGCTCAGGCCAGCCTGCCTCGTGATATTATCTTCCATAATGCTGGGACCCGC

AGTCCACCTACGTCGCTGCTGTGTCCCCTA

>Malaysia 2...Pompano...............2006....Cell....GQ904199

GAGATTGCGAACAATGACCCTTTGTTCCATTTAGTGATATACCCTATCCGCGTGTTCCCTACTTAGGTTCCTCCCCGCTATATGAAGTCCCCCTGTTGGT

CGATAGCCATGGGTTATTATCTGACTCTATGTTCAATCAACTAACACACGTTCAGGCCAGCCTGCCTCGTGATATCTTTTTCCATAATGCTGGGACCTGC

AGTCCACCTACGCCGCTGCTGTGTCCCCTA

>Malaysia 2...Pompano...............2008....Fish....HQ859932

GAGATTGCGAACAGTGACCCTTTGTCCCATTTAGTGATATACCCTATCCGCGTGTCCCCTACTTAGGTTCCTCCCCGCTATATGAAGTCCTCCCGTTGGT

CGATAGCCATGAGTTACTATCTGAGTCTATGTTCAATCGACTAACACACGTTCAGGCCAGCCTGCCTCGTGATATCTTTTTCCATAATGCTGGGACCCGC

AGTCCACCTACGTCGCTGCTGTGTCCCCTA

>Malaysia 1a..Barramundi............2007(F).Fish....HQ859919

GAGATTGCGAACAGTGACCCTTTGTCCCATTTAGTGATATACCCTATCCGCGAGTTCCCTACTTAGGTTCCTCTCCGCTATAAGAAGTCCTCCCGTTGGT

CGATAGCCACGGGTTACTATCTGGCTCTATGTTCAATCGACTAACACACGCTCAGGCCAGCCTGCCTCGTGGTATCTTTCTCCATAATGCTGGGACCCGC

AGTCTACCTATGTCGCTGCTGTGTCCCCTA

>Malaysia 1a..Barramundi............2007(J).Fish....HQ859922

GAGATTGCGAACAGTGACCCTTTGTCCCATTTAGTGATATACCCTATCCGCAAGTTCCCTGCTTAGGTTCCTCTCCGCTATATGAAGTCCTCCCGTTGGT

CGATAGCCACGGGTTACTATCTGGCTCTATGTTCAATCGACTAACACACGCTCAGGCCAGCCTGCCTCGTGATATCTTTCTCCATAATGCTGGGACCCGC

AGTCTACGTATGTCGGGACTGTGGACCCTA

>Malaysia 1d..Barramundi............2008....Fish....GQ120525

GGGATTGCGAAAAGTGACCTTTTGTCCCATTAAGAAATATACCCTATCCGCGAGTTCCCTACTTAGGTTCCTCTCCGTTATATGAAGTCCACCCGTTGGT

CGATAGCCGCGGGTTACCGTCTGATTTTATGTGTAATCGACTAACACACGCTCAGGCCAGCCTGCTTCGTGATGTCTTTCTCCATAATGTTACGACCCAC

AGTCTACCTATGTTGCTGCTGTGTCCCCTA

>Malaysia 1a..Barramundi............2008(A).Fish....HQ859930

GAGATTGCGAACAGTGACCTTTTGTCCCATTAAGAAATATACCCTATCCGCGAGTTCCCTACTTAGGTTCCTCTCCGCTATATGACAGTCACCCGTTGGT

CGATAGCCACGGGTTACCGTCTGATTTTATGTGTAATCGACTAACACACGCTCAGGCCAGCCTGCTTCGTGATATCTTCCTCCATAATGTTACGACCCAC

AGTCTACCTATGTTGCTGCTGTGTCCCCTA

>Malaysia 1a..Barramundi............2008(N).Fish....HQ859935

GAGATTGCGCACAGTGACCTTTTGTCCCATCAAGAAATATACCCTATCCGCGAGTTCCCTACTTAGGTTCCTCTCCGCTATATGAAGTCCACCCGTTGGT

CGATAGCCACGGGTTACCGTCTGATTTTATGTGTAATCGACTAACACACGCTCAGGCCAGCCTGCTTCGTGATATCTTCCTCCATAATGTTACGACCCAC

AGTCTACCTATGTTGCTGCTGTGTCCCCTA

>Malaysia 1a..Barramundi............2009(J).Fish....HQ859943

GAGATTGCGAACAATGACCCTTTGTCCCATTTAGTGATATACCCCATCCGCGAGTTCCCTACTTAGGTTCCTCTCCGCTATATGAAGTCCCCCCGTTGGT

CGATAGCCACGGGTTACTATCTGGCGCCATGTTCAATCGACTAACACACGCTCGGGCCAGCCTGCCTCGTGATATCTTTTTCCATAATGCTGGGACCCGC

AGTCTACCTATGTCGCTGCTGTGCCCCCTA

>Malaysia 1c..Barramundi............2009(S).Fish....HQ859948

GAGATTGCGAACAATGACCCTTTGTCCCATTTAGTGATATACCCTATCCGCGAGTTCCCTACTTAGGTTCCTCTCCGCTATATGAAGTCCCCCCGTTGGT

CGATAGCCACGGGCTACTATCTGGCTCTATGTTCAATCGACTAACACACGCTCAGGCCAGCCTGCCTCGTGACATCTTTCTCCATAATGCTGGGACCCGC

AGTCTACCTATGTCGCTGCTGTGTCCCCTA

>Malaysia 1b..Grouper (Tiger).......2008(D).Fish....HQ859938

GAGATTGCGAACAGTGACCTTTTGTCCCATTAAGAAATATACCCTATCCGCGAGTTCCCTACTTAGGTTCCTCTCCGCTATATGAAGTCCACCCGTTGGT

CGATAGCCACGGGTTACCGTCTGATTTTATGTGTAATCGACTAACACACGCTCAGGCCAGCCTGCTTCGTGATATCTTCCTCCATAATGTTACGACCCAC

AGTCTACCTATGTTGCTGCTGTGTCCCCTA

>Malaysia 1b..Grouper (Tiger).......2008(D).Fish....HQ859939

GAGATTGCGAACAGTGACCTTTTGTCCCATTAAGAAATATACCCTATCCGCGAGTTCCCTACTTAGGTTCCTCTCCGCTATATGAAGTCCACCCGTTGGT

CGATAGCCACGGGTTACCGTCTGATTTTATGTGTAATCGACTAACACACGCTCAGGCCAGCCTGCTTCGTGATATCTTCCTCCATAATGTTACGACCCAC

AGTCTACCTATGTTGCTGCTGTGTCCCCTA

>Malaysia 1b..Grouper (Tiger).......2009(A).Fish....HQ859945

GAGATTGCGAACAGTGACCCTTTGTCCCATTTAGTCATATACCCTATCCGCGAGTTCCCTACTTAGGTTCCTCTCCGCTATATGAAGTCCTCCCGTTGGT

CGATAGCCACGGGTTACTATCTGGCTCTATGTTCAATCGACTAACACACGCTCAGGCCAGCCTGCCTCGTGATATCTTTCTCCATAATGCTGGGACCCGC

AGTCTACCTATGTCGCTGCTGTGTCCCCTA

>Malaysia 1b..Grouper (Tiger).......2009(A).Fish....HQ859946

GAGATTGCGAACAGTGACCCTTTGTCCCATTTAGTCATATACCCTATCCGCGAGTTCCCTACTTAGGTTCCTCTCCGCTATATGAAGTCCTCCCGTTGGT

CGATAGCCACGGGTTACTATCTGGCTCTATGTTCAATCGACTAACACACGCTCAGGCCAGCCTGCCTCGTGATATCTTTCTCCATAATGCTGGGACCCGC

AGTCTACCTATGTCGCTGCTGTGTCCCCTA

>Malaysia 1b..Grouper (Tiger).......2009(A).Fish....HQ859947

GAGATTGCGAACAGTGACCCTTTGTCCCATTTAGTCATATACCCTATCCGCGAGTTCCCTACTTAGGTTCCTCTCCGCTATATGAAGTCCTCCCGTTGGT

CGATAGCCACGGGTTACTATCTGGCTCTATGTTCAATCGACTAACACACGCTCAGGCCAGCCTGCCTCGTGATATCTTTCTCCATAATGCTGGGACCCGC

AGTCTACCTATGTCGCTGCTGTGTCCCCTA

>Singapore....Grouper (Greasy)......1991....Cell....AF281657

GAGATTGCGAACAGTGACCCTTTGTTCCATTTAGTAACATACCCTATCCGCGAGTTCCCTACTTAGGTTCCTCTCCGCTATATGAAGTCCTTTCGTTGGT

CGGTAGCCATGAGTTACTATCTGACTCTATGTTCACTCGACTAACACACGCTCAGGCCAGCCTGCCTCGTGATATCTTTTTCCATAATGCTGGGACCCGC

AGTCAACCTACGTCGCTGCTGTGTCCCCTA

>Singapore....Grouper (Greasy)......2001....Cell....AF318942

GAGATTGCGAACAGTGACCCTTTGTTCCATTTAGTAACATACCCTATCCGCGAGTTCCCTACTTAGGTTCCTTTCCGCTATATGAAGTCCTCCCGTTGGT

CGATAGCCATGAGTTACTATCGGACTCTATGTTCAATCGACTAACACACGCTCAGGCCAGCCTGCCTCTTGATATCTTTTTCTATAATGCTGGGACTTGC

AGTCTACCTACGTCGCTGCTGTGTCCCCTG

>Singapore....Guppy.................2000....Cell....AF499774

GATATTGCGAACAGTGTCCCTTTGTTCCATTTAGTAACATAATCTATCCGCGAGTTCCCTACTTAGGTTCCTCTCCGCTATATGAAGTCCTCCCGTTGGT

TGATAGCCATGAGTTACTATCTGACTCTATGTTCACTCGATTAACACACGCTCAGGCCAGCCTGCCTCCTGATATCTTTTTCCATAATGCTAGGACCCGC

AGTCCACCTACGTCGCTGCTGTGTCCTCTA

>Vietnam......Grouper (Humpback)....2015....Fish....USC Vie1

GAGATTGCGAACAGTGACCCTTTGTTCCATTTAGTGATATACCCTATCCGCGTGCTCCCTACTTAAGTTCCTCCCCGCTATATGAAGTCCTCCTGTTGGT

CGATAGCCATGAGTTACTATCTGACTCTATGTTCAATCGACTAACGCACGTTCAGGCCAGCCTGCCCCGTGATATTTTTTTCCATAATGCTGGGACCTGC

AGTCCACCTACGTCGCTGCTGTGTCCCCTA

>Vietnam......Grouper (Humpback)....2015....Fish....USC Vie2

GAGATTGCGAACAGTGACCCTTTGTTCCATTTAGTGATATACCCTATCCGCGTGCTCCCTACTTAAGTTCCTCCCCGCTATATGAAGTCCTCCTGTTGGT

CGATAGCCATGAGTTACTATCTGACTCTATGTTCAATCGACTAACGCACGTTCAGGCCAGCCTGCCCCGTGATATTTTTTTCCATAATGCTGGGACCTGC

AGTCCACCTACGTCGCTGCTGTGTCCCCTA

>Vietnam......Grouper (Humpback)....2015....Fish....USC Vie3

GAGATTGCGAACAGTGACCCTTTGTTCCATTTAGTGATATACCCTATCCGCGTGCTCCCTACTTAAGTTCCTCCCCGCTATATGAAGTCCTCCTGTTGGT

CGATAGCCATGAGTTACTATCTGACTCTATGTTCAATCGACTAACGCACGTTCAGGCCAGCCTGCCCCGTGATATTTTTTTCCATAATGCTGGGACCTGC

AGTCCACCTACGTCGCTGCTGTGTCCCCTA

>Vietnam......Grouper (Humpback)....2015....Fish....USC Vie4

GAGATTGCGAACAGTGACCCTTTGTTCCATTTAGTGATATACCCTATCCGCGTGCTCCCTACTTAAGTTCCTCCCCGCTATATGAAGTCCTCCTGTTGGT

CGATAGCCATGAGTTACTATCTGACTCTATGTTCAATCGACTAACGCACGTTCAGGCCAGCCTGCCCCGTGATATTTTTTTCCATAATGCTGGGACCTGC

AGTCCACCTACGTCGCTGCTGTGTCCCCTA

>Vietnam......Grouper (Orange spot).2007....Fish....EF492143

GAGATTGCGAACAGTGACCCTTTGTTCCATTTAGTGATATACCCTATCCGCGTGCTCCCTACTTAGGTTCCTCCCCGCTATATGGAGTCCTCCTGTTGGT

CGATAGCCATG-GTTACTATCTGACTCTATGTTCAATCGACTAACACACGTTCAGGCCAGCCTGCCTCGTGATATCTTTTTCCATAATGCTGGGACCTGC

AGTCCACCTACGTCGCTGCTGTGTCCCCTA

>Taiwan.......Grouper (Giant-Tiger).2015....Fish....USC Tai1

GAGATTGCGAACAGTCACCCTTTGTTCCATTTAGTGATATACCCTATTCGCGTGCTCCCTACTTAGGTTCCTCCCCGCTATATGAAGTCCTCCTGTTGGT

CGATAGTCATGAGTTACTATCTGACTCTATGTTCAATCGGCTAACACACGTTCAGGCCAGCCTGCCTCGTGATATTTTTTTCCATAATGCTGGGACCTGC

AGTCCACCTACGTCGCTGCTGTGTCCCCTA

>Taiwan.......Grouper (Giant-Tiger).2015....Fish....USC Tai2

GAGATTGCGAACAGTCACCCTTTGTTCCATTTAGTGATATACCCTATTCGCGTGCTCCCTACTTAGGTTCCTCCCCGCTATATGAAGTCCTCCTGTTGGT

CGATAGTCATGAGTTACTATCTGACTCTATGTTCAATCGGCTAACACACGTTCAGGCCAGCCTGCCTCGTGATATTTTTTTCCATAATGCTGGGACCTGC

AGTCCACCTACGTCGCTGCTGTGTCCCCTA

>Taiwan.......Grouper (Giant-Tiger).2015....Fish....USC Tai3

GAGATTGCGAACAGTCACCCTTTGTTCCATTTAGTGATATACCCTATTCGCGTGCTCCCTACTTAGGTTCCTCCCCGCTATATGAAGTCCTCCTGTTGGT

CGATAGTCATGAGTTACTATCTGACTCTATGTTCAATCGGCTAACACACGTTCAGGCCAGCCTGCCTCGTGATATTTTTTTCCATAATGCTGGGACCTGC

AGTCCACCTACGTCGCTGCTGTGTCCCCTA

>Taiwan.......Grouper (Giant).......2000....Cell....AF245004

GAAATTGCCAACAGTGACCCTTTGTTCCGTTTAGTGATATACCCTATCCGCGAGTTCCCTACTTAGGTTCCTCCCCGCTATATGAAGTCCTCCCGTTGGT

CGATAGCCATGAGTTACTATCTGACTCTATGTTCAATTGACTAACACACGCTCAGGCCAGCTAGCCTCGTGATATCTTTCTCCATAATGCTGGGACCCGC

AGTCCACCTACGTCGCTGCTGTGTCCCCTA

>Taiwan.......Grouper (Giant).......2013....Cell....KM588181

GAGATTGCGAACAGTGACCCTTTGTTCCATTTAGTGATATACCCTATCCGCGTGCTCCCTACTTAGGTTCCTCCCCGCTATATGAAGTCCTCCTGTTGGT

CGATAGCCATGAGTTACTATATGACACTATGTTCACTCGACTAACACACGTTCAGGCCAGCCTGCCTCGTGATATTTTTTTCCATAATGCTGGGACCTGC

AGTCCACCTACGTCGCTGCTGTGTCCCCTA

>Taiwan.......Grouper (Greasy)......2000....Cell....AF245003

GAAATTGCGAACAGTGACCCTCTGCTCCATTTAGTAATATACCCTATCCGCGAGTTTCCTGCTTAGGTTCCTCTCCGCTATATGAAGTCCTCCCGTAGGT

CGATAGCCATGAGTTACTATCTGACTCTATGTTCAATCAACTAACACACGCTCAGGCCAGCCTGCCTCGTGATATCTTTCTCCATAATGCTGGGACCCGC

AGTCCACCTACGTCGCTGCTGTGTCCCCTA

>Taiwan.......Grouper (Red spot)....2003....Cell....EU391590

GAGATTGCGAACAGTGACCCTTTGTTCCATTTAATAGTATACCCTATCCGCGAGTTCCCTACTTAGGTCCTTCTCCGCTATATGAAGTCCTCCTGTTGGT

CGATAGCCATGAGTTACTATCTGACTCTATGTTCAATCGACTAATACACGCTCATGCCAGCCCGCCTCGTGATATCTTTCTCCATAATGCTAGGATCCGT

AGTCTACCTATGTCGCTGCTGTGTCCCCTG

>Taiwan.......Grouper (Orange spot).2011....Cell....KT071606

GAGATTGAGAACAGTGACCCTTTGTTCCATTTAGTGATATACCCTATCCGCGTGCTCGTTACTTAGGTTCCTCCCCGCTATATGAAGTCCTCCTGTTGGT

CGATAACCATGGGTTACTATCTGACTCTATGTTCAATCGACTAACACGCGTTCAGGCCAGCCTGCCTCGTGATATCTTTCTCCATAATGCTGGGACCTGC

AGTCCACCTACGTCGCTGCTGTGTCCCCTA

>Taiwan.......Grouper species.......1995....Cell....AY690596

GAGATTGCGAACAGTGACCCTTTGTTCCATTTAGTAATATACCCTATCCGCGAGTTCCTTACTTAGGTTCCTCCCCGCTATATGAAGTCCTCCCGTTGGT

CGATAGCCATGAGCTACTATCTGACTCTATGTTCAATCGACTAACACACGCTCAGGCCAGCCTGCCTCGTGATATCTTCTTCCATAATGCTGGGACCCGC

GGTCCACCTATGTCGCTGCTGTGTCCCCTA

>Taiwan.......Lutjanus..............2004....Unknown.AY835642

GAGATTGCGAACAATGACCCTTTGTTCCATTTAGTGATATACCCTATCCGCGTGTTCCCTACTTAGGTTCCTCACCGCTATATGAAGTCCCCCTGTTGGT

CGATAGCCATGGGTTATTATCTGACTCTATGTTCAATCGACTAACACACGTTCAGGCCAGCCTGCCTCGTGATATCTTTTTCCATAATGCTGGGACCTGC

AGTCCACCTACGCCGCTGCTGTGTCCCCTA

>China(C).....Grouper (Giant).......2004....Unknown.AY721615

GAGATTGCGAACAGTGACCCTTTGTCCCATTTAGTGATATACCCTATCCGCGAGTTCCCTACTTAGGTTCCTCTCCGCTATATGAAGTCCTCCCGTTGGT

CGATAGCCACGGGTTACTATCTGGCTCTATGTTCAATCGACTAACACACGCTCAGGCCAGCCTGCCTTGTGATATCTTTCTCCATGATGCTGGGACCCGC

AGTCTACCTATGTCGCTGCTGTGTCCCCTA

>China(S).....Grouper (Red spot)....2000....Fish....AY744705

GAGATTGCGAATAGTGACCCTTTGTTCCATTTAGTAATATACCCTGTCCGCGAGTTTCCTACTTAGGTTCCTCTCCGCTATATGAAGTCCTCCCGTTGGT

CGATAGCCATGAGTTACTATCTGACTCTATGTTCAATCGACTAACACACGCTCAGGCCAGCCTGCCTCGTGATATCTTTCTCCATAATGCTGGGACCCGC

AGTCCACCTACGTCGCTGCTGTGTCCCCTA

>China(S).....Grouper (Red spot)....2006....Cell....EF558369

GAGATTGCGAACAGTGACCCTTTGTTCCATTTAGTAATATACCCTATCCGCGAGTTCCCTACTTAGGTTCCTCTCCGCTATATGAAGTCCCCCCGTTGGT

CGATAGCCATGAGTTATTATCTGATTCTATGTTCACTCGACTAACACACGCTCAGGTCAGCCTGCTTCGTGATATCTTTTTACATAATGCTGGGACCCGC

AGTCCACCTACGTTGCTGCTGTGTCCCCTG

>China(S).....Grouper (Orange spot).2003....Unknown.AF534998

GAGATTGCGAACAGTGACCCTTTGTTCCATTTAGTGATATACCCTATCCGCGTGTCCCCTACTTAGGTTCCTCCCCGCTATATGAAGTCCTCCCGTTGGT

CGATAGCCATGAGTTACTATCTGACTCTATGTTCAATCGACTAACACACGTTCAGGCCAGCCTGCCTCGTGATATCTTTTTCCATAATGCTGGGACCCGC

AGTCCACCTACGTCGCTGCTGTGTCCCCTA

>China(S).....Pompano...............2009....Fish....GU339227

GAGATTGCGAACAGTGACCCTTTGTTCCATTTAGTGATATACCCTATCCGCGTGCTCCCTATTTAGGTTCCTCCCCGCTATATGAAGTCCTCCTGTTGGT

CGATAGCCATGGGTTACTATCTGACTCTACGTTCAATTGACTAACACACGTTCAGGCCAGCCTGCCTCGTGATATCTTTTTCCATAATGCTGGGACCTGC

AGTCCACCTACGTCGCTGCTGTGTCCCCTA

>China(S).....Sea Perch.............2014....Fish....KP455642

GAGATTGCGAACAGTGACCCTTTGTTCCATTTAGTGATATACCCTATCCGCGTGTTCCCTACTTAGGTTCCTCCCCGCTATATGAAGTCCTCCCGTTGGT

CGATAGCCATGGGTTACTATCTGACTCTGTGTTCAATCGACTAACACACGTTCAGGCCAGCCTGCCTCGTGATATTTTTTTCCATAATGCTGGGACCCGC

AGTCCACCTACGTCGCTGCTGTGTCCCCTA

>China(N).....Grouper (Giant-Tiger).2013....Unknown.KF668183

GAGATTGCGAACAGTGACCCTTTGTTCCATTTAGTGATATACCCTATCCGCGTGCTCCCTATTTAGGTTCCTCCCCGCTATATGAAGTCCTCCTGTTGGT

CGATAGCCATGGGTTACTATCTGACTCTATGTTCAATCGACTAACACACGTTCAGGCCAGCCTGCCTCGTGATATTTTTTTCCATAATGCTGGGACTTGC

AGTCCACCTACGTCGCTGCTGTGTCCCCTA

>China(N).....Flounder(Olive).......2006....Unknown.KF841612

GAGATTGCGAACAGTGACCCTTTGTTCCATTTAGTGATATACCCTATCCGCGTGCTCCCTACTTAGGTTCCTCCCCGCTATATGAAGTCCTCCTGTTGGT

CGACAGCCATGGGTTACTATCTGACTCTATGTTCAATCGACTAACACACGTTCAGGCCAGCCTGCCTCGTGATATCTTTTTCCATAATGCTGGGACCTGC

AGTCCACCTACGTCGCTGCTGTGTCCCCTA

>China(N).....Sole..................2012....Fish....KJ541748

GAGATTGCGAACAGTGACCCTTTGTTCCATTTAGTGATATACCCTATCCGCGTGCTCCCTATTTAGGTTCCTCCCCGCTATATGAAGTCCTCCTGTTGGT

CGATAGCCATGGGTTACTATCTGACTCTATGTTCAATCGACTAACACACGTTCAGGCCAGCCTGCCTCGTGATATTTTTTTCCATAATGCTGGGACTTGC

AGTCCACCTACGTCGCTGCTGTGTCCCCTA

>Korea........Grouper (Sevenband)...2005....Unknown.DQ116036

GGGATTGCGAACAGTGACCCTTTGTTCCATTTAGTAATATACCCTATCCGCGAGTTCCCTACTTAGGTTCCTCTCCGCTATATGAAGTCCTCCCGTTGGT

CGATAGCCACGGGTTACTATCTGGCTCTATGTTCAATCGACTAACACACGCTCAGGTCAGCCTGCCTCGTGATATCTTTTTACATAATGCTGGGACTCGC

AGTCTACCTATGTCGCTGCTGTGTCCCCTA

>Korea........Grouper (Sevenband)...2008....Cell....KM095959

GAGATTGCGAACAGTGACCCTTTGTTCCATTTAGTGATATACCCTATCCGCGTGCTCCCTACTTAGGTTCCTCCCCGCTATATGAAGTCCTCCTGTTGGT

CGATAGCCATGGGTTACTATCTGACTCTGTGTTCAATCGACTAACACACATTCAGGCCAGCCTGCCTCGTGATATCTTTTTCCATAATGCTGGGACCTGC

AGTCCACCTACGTCGCTGCTGTGTCCCCTA

>Korea........Flounder(Japanese)....2006....Unknown.DQ116037

GGGATTGCGAACAGTAAACCTTTGTTCCATTTAGTAATATACCCTATCCGCGAGTTCCCTACTTAGGCTCCTCTCCGCTATATGAAGTCCTCCCGTTGGT

CGATAGCCATGGGTTACTATCTGACTCTATGTTCAATCGACTAACACACGCTCAGGCCAGCCTGCCTCGTGATATCTTGTTCCATAATGCTGGGACCCGC

GGTCCACCTATGTCGCTGCTGTGTCCCCTA

>Korea........Flounder(Japanese)....2006....Unknown.DQ864760

GGGATTGCGAACAGTGACCCTTTGTTCCATTTAGTAATGTACCCTATCCGCGGATTCCCTACTCAGGTTCCTCTCCGCTATATGAAGTCCTCCCATTGGT

CGATAGCCATGGGTTACTATCTGGTTCTATGTTCAATCGACTAACACACGCTCAGGCCAGCCTGCCTCGTGATAGCTTTTTCCATAATGCTGGGACCTGC

AGTCTACCTATATCGCTGCTGTGTCTCCTA

>Korea........Bream.................2005....Unknown.DQ116035

GGGATTGCGAACAGTGACCCTTTGTTCCATTTAGTAATATACCCTATCCGCGAGTTCCCTACTTAGGTTCCTCTCCGCTATATGAAGTCCTCCCGTTGGT

CGATAGCCATGAGTTACTATCTGACTCTATGTTAAATCGACTAACACACGCTCAGGCCAGCCTGCCTCGTGATATCTTTCTCCATAATGCTGGGACTCGC

GGTCCACCTATGTCGCTGCTGTGTCCCCCA

>Korea........Mullet................2005....Unknown.DQ116038

GGGATTGCGAACAGTGACCCTTTGTTCCATTTAGTAATGTACCCTATCCGCGAGTTCCCTACTTAGGTTCCTCTCCGCTATATGAAGTCCTCCCATTGGT

CGATAGCCATGGGTTACTATCTGGTTCTATGTTCAATCGACTAACACACGCTCAGGCCAGCCTGCCTCGTGATAGCTTTTTCCATAATGCTGGGACCCGC

AGTCTACCTATGTCGCTGCTGTGTCCCCTA

>Japan........Grouper (Sevenband)...2001....Cell....AB373029

GAGATTGCGAACAGTGACCCTTTGTTCCATTTAGTAATATACCTTATCCGCGAGTTTCCTACTTAGGTTCCTCTCCGCTATATGAAGTCCTCCCGTTGGT

CGATAGCCATGAATTACTATCTGACTCTATGTTCAATCGACTAACACACGCTCAGGCCAGCCTGCCTCGTGATATCTTTCTCCATAATGCTGGGACCCGC

AGTCCACCTACGTCGCTGCTGTGTCCCCTA

>Japan........Grouper (Sevenband)...2004....Cell....AY324870

GAGATTGCGAACAGTGACCCTTTGTTCCATTTAGTAATATACCCTATCCGCGAGTTCCCTACTTAGGTTCCTCTCCGCTATATGAAGTCCTCCCGTTGGT

CGATAGCCATGAGTTACTATCTGACTCTATGTTCAATCGACTAACACACGCTCAGGCCAGCCTGCCTCGTGATATCTTTTTCCATAATGCTGGGACCCGC

GGTCCACCTATGTCGCTGCTGTGTCCCTTA

>Consensus

GAGATTGCGAACAGTGACCCTTTGTTCCATTTAGTGATATACCCTATCCGCGAGTTCCCTACTTAGGTTCCTCCCCGCTATATGAAGTCCTCCCGTTGGT

CGATAGCCATGAGTTACTATCTGACTCTATGTTCAATCGACTAACACACGCTCAGGCCAGCCTGCCTCGTGATATCTTTTTCCATAATGCTGGGACCCGC

AGTCCACCTACGTCGCTGCTGTGTCCCCTA

# Supplementary Figure S5. Significance levels for characteristic attributes.

* P<0.05; ** P<0.01

# Supplementary Figure S6. Similarity matrix for only polymorphic nucleotide positions RNA 2 NNV for 82 samples.

# Supplementary Figure S7. Similarity matrix for translated protein sequences from the RNA 2 NNV for 82 samples.
